# Comparative safety profiles of spironolactone, eplerenone, and finerenone: a pharmacovigilance study based on FAERS data from 2004 to 2024

**DOI:** 10.3389/fphar.2025.1702257

**Published:** 2026-01-07

**Authors:** Kaiyun Ji, Xingjun Huang, Fang Wang, Lei Zhang, Yifan Zheng, Ligang Ji, Ying Ju, Xiaodong Zhuang, Jia Li

**Affiliations:** 1 Department of Pharmacy, The First Affiliated Hospital of Sun Yat-sen University, Guangzhou, China; 2 Department of Pharmacy, Jincheng General Hospital, Jincheng, China; 3 Department of Pharmacy, Huizhou Central People’s Hospital, HuiZhou, China; 4 Department of Pharmacy, The Affiliated Hospital of Guizhou Medical University (Guizhou Hospital, The First Affiliated Hospital of Sun Yat-sen University), Guiyang, China; 5 Department of Pharmacy, Shanxi Provincial Integrated TCM And WM Hospital, Taiyuan, China; 6 Department of Clinical Pharmacy Translational Science, University of Michigan College of Pharmacy, Ann Arbor, MI, United States; 7 Department of Cardiology, The First Affiliated Hospital, Sun Yat-sen University, Guangzhou, China; 8 Department of Pharmacy, Guangxi Hospital Division of The First Affiliated Hospital, Sun Yat-sen University, Nanning, China

**Keywords:** FAERS database, mineralocorticoid receptor antagonists (MRAs), adverse event signals, safety analysis, disproportionality analysis, data mining

## Abstract

**Background:**

Spironolactone, eplerenone, and finerenone are three commonly used mineralocorticoid receptor antagonists (MRAs) with cardioprotective and renoprotective effects. However, comparative real-world safety evaluations remain limited. This study aimed to assess and compare the adverse event (AE) profiles of the three MRAs using data from the U.S. Food and Drug Administration Adverse Event Reporting System (FAERS).

**Method:**

We conducted a retrospective descriptive analysis using FAERS data from Q1 2004 to Q4 2024. Four disproportionality methods were employed to detect AE signals, including Reporting Odds Ratio (ROR), Proportional Reporting Ratio (PRR), Bayesian Confidence Propagation Neural Network (BCPNN), and Multi-item Gamma Poisson Shrinker (MGPS). Key safety concerns, such as hyperkalaemia, renal impairment, sexual side effects, and congenital anomalies related to MRAs, were examined.

**Results:**

A total of 8,625 AE reports were identified for spironolactone, 2,045 for eplerenone, and 1,391 for finerenone. The most commonly affected system organ classes were metabolism and nutrition disorders, renal and urinary disorders, and investigations. Hyperkalaemia (1,333 cases, ROR 93.73) and acute kidney injury (1,110 cases, ROR 13.11) were the most frequently reported AEs for spironolactone, while eplerenone showed notable signals for acute kidney injury (263 cases, ROR 14.58) and hyperkalaemia (154 cases, ROR 47.79). Finerenone exhibited particularly strong signals for decreased glomerular filtration rate (179 cases, ROR 434.80) and hyperkalaemia (152 cases, ROR 116.85). Spironolactone was uniquely associated with rare but severe AEs such as male endometriosis (ROR 13,978.20) and 5-α-reductase deficiency (ROR 1,663.95), suggesting significant hormonal effects. Eplerenone showed signals for decreased jugular venous pressure and drug–disease interactions. Finerenone was associated with renal biomarkers but lacked significant sex hormone–related AEs. In terms of AEs involving potassium imbalance or renal impairment, finerenone presented the strongest signals, followed by eplerenone and spironolactone. However, spironolactone showed the highest incidence of sexual and congenital abnormalities.

**Conclusion:**

The three MRAs exhibit distinct AE profiles. Hyperkalaemia and renal impairment are shared concerns, while spironolactone requires special attention to hormonal and developmental side effects. Eplerenone shows fewer androgen-related AEs, and finerenone appears safer in this regard. Further clinical studies are needed to validate these findings and inform safer prescribing practices.

## 1 Introduction

Cardiovascular-kidney-metabolic (CKM) syndrome is a clinical condition defined by the coexistence of cardiovascular disease (CVD), chronic kidney disease (CKD), and metabolic disorders. Its global prevalence is rising due to aging populations and sedentary lifestyles (Ndumele et al., 2023a). The co-occurrence of CVD and CKD results in a significantly higher mortality risk than either condition alone, largely due to shared risk factors such as inflammation, oxidative stress, and endothelial dysfunction (Ndumele et al., 2023b; Mazzieri et al., 2024; Yang et al., 2019). The renin-angiotensin-aldosterone system (RAAS) plays a central role in the pathogenesis of CKD and CVD. However, aldosterone escape remains a frequent limitation (Mazzieri et al., 2024; Yang et al., 2019), occurring in up to 50% of patients within the first year (Bomback and Klemmer, 2007). This has highlighted the value of mineralocorticoid receptor antagonists (MRAs), which suppress aldosterone-induced inflammation and fibrosis, thereby improving renal and cardiovascular outcomes (Zannad et al., 2000). However, at the same time, they are limited in clinical use due to possible safety issues such as early renal impairment (Zannad et al., 2000; Marzolla et al., 2022). This reminds us of the importance of early understanding of their adverse effects and preventing them.

Spironolactone, the first MRA introduced in 1960, remains widely used due to its mortality benefits (Pitt et al., 1999). However, its nonspecific binding to sex hormone receptors often results in adverse effects such as gynaecomastia, limiting its clinical acceptance. Eplerenone, approved by FDA in 2002, emerged as an alternative to spironolactone due to its high selectivity for the mineralocorticoid receptor (MR). It has since become the preferred MRA in international clinical practice. Both spironolactone and eplerenone are steroidal MRAs and form part of the standard, evidence-based treatment for patients with heart failure with reduced ejection fraction (HFrEF), left ventricular dysfunction following a myocardial infarction, and resistant hypertension (Byrne et al., 2023; McEvoy et al., 2024). However, their use in the management of CKD remains limited, largely due to concerns about cross-reactivity with sex hormone receptors and the risk of hyperkalaemia (Marzolla et al., 2022). In recent years, the introduction of the third-generation MRAs has expanded therapeutic options for patients with kidney diseases (Agarwal et al., 2022). Finerenone, a non-steroidal third-generation MRA, was approved in the United States in 2021. It exhibits the strongest inhibitory activity against MR among MRAs, with a more balanced tissue distribution (Di Lullo et al., 2023; Bakris et al., 2020). Unlike steroidal MRAs, finerenone has minimal affinity for glucocorticoid, androgen, and progesterone receptors, thereby reducing the risk of hormone-related adverse effects. These pharmacological characteristics confer significant renoprotective benefits in diabetic patients with CKD and help prevent cardiovascular complications associated with diabetic nephropathy. The FINEARTS-HF trial demonstrated that finerenone significantly reduced the composite incidence of heart failure worsening and cardiovascular death in patients with mildly reduced or preserved ejection fraction. It was also associated with improved health-related quality of life compared to placebo (Vaduganathan et al., 2024; Ostrominski et al., 2025). On July 11, 2025, Eastern Time, the FDA officially approved finerenone for adult patients with heart failure ≥40% of LVEF to reduce the risk of cardiovascular death, heart failure hospitalization, and heart failure emergency department visits. These findings suggest that MRAs could benefit all heart failure subtypes, thereby expanding their use in heart failure treatment.

As the clinical use of the three MRAs has expanded, reports of AEs have also increased. Common AEs associated with spironolactone include hyperkalaemia, hypotension, renal function deterioration, electrolyte and metabolic abnormalities, and gynaecomastia (Byrne et al., 2023). Reported AEs for eplerenone include hyperkalaemia, hyperuricaemia, headaches, dizziness and muscle cramps (Struthers et al., 2008; Pitt et al., 2008; Provenzano et al., 2022). Finerenone is primarily associated with hyperkalaemia, hyponatraemia and hypotension (Bakris et al., 2020). However, direct comparisons of the safety profiles of these three agents based on real-world data remain limited.

This study investigates the AE profiles of spironolactone, eplerenone, and finerenone, with a particular focus on hyperkalaemia, renal impairment, congenital anomalies, and sex hormone-related issues. The analysis is based on the most recent data from the FAERS database. By systematically evaluating these AEs, the study aims to characterize the safety profiles of each drug, offering valuable guidance for clinical risk management and promoting safer, more rational use of MRAs in practice.

## 2 Materials and methods

### 2.1 Data source and collection

This retrospective pharmacovigilance study utilized data from the FAERS database (https://fis.fda.gov/extensions/FPD-QDE-FAERS/FPD-QDE-FAERS.html), a publicly accessible platform that collects information on spontaneously reported AEs. Data were extracted by querying for the generic drug names “spironolactone”, “eplerenone”, and “finerenone” across 84 quarters from Q1 2004 to Q4 2024. Only reports where the target drug was identified as the primary suspect (PS) were included. Reports have been deduplicated following the FDA guidelines (Hu et al., 2020), duplicates with identical CASEID values were resolved by retaining the most recent FDA_DT. When both CASEID and FDA_DT were identical, the record with the higher PRIMARYID was prioritized. We used system organ classes (SOCs) and preferred terms (PTs) from the Medical Dictionary for Regulatory Activities (MedDRA version 27.1), retrieving AE reports at the PT level.

### 2.2 Safety signals identification and disproportionality analysis

Currently, there is no universally accepted gold standard methodology for detecting safety signals (Bate and Evans, 2009). In this study, we applied four well-established disproportionality methods to identify AE signals, including the Reporting Odds Ratio (ROR), Proportional Reporting Ratio (PRR), Bayesian Confidence Propagation Neural Network (BCPNN), and Multi-item Gamma Poisson shrinker (MGPS) (Noguchi et al., 2021). The thresholds and calculation methods for the four algorithms are detailed in Supplementary Tables S1, S2. Using a single method alone may increase the likelihood of false positives. Each method has its advantages and limitations, and combining them enhances the reliability of signal detection. While PRR is useful for estimating relative risk, it is highly sensitive and may generate false-positive signals, especially when the number of reports is low. ROR assesses the strength of correlation between drug exposure and specific outcomes. BCPNN excels in identifying variants and extracting key information from complex, multi-variable data systems. MGPS is effective in detecting hidden signals by analyzing the relationship between drugs and AEs, which traditional methods might overlook. These metrics were calculated using two-by-two contingency tables, comparing the reported event counts for a specific drug against all other drugs. For ROR (Van Puijenbroek et al., 2003), a signal was considered positive if the number of reports (α value) was ≥3 and the lower limit of 95% Confidence Interval (*CI*) value >1. For PRR (Evans et al., 2001), a signal was detected if the number of reports (α value) ≥ 3, *PRR* value >2, and variance (*χ* ^*2*^) > 4. For BCPNN (Norén et al., 2006), a signal was identified if the number of reports (α value) ≥ 3 and the lower limit of *IC025* value >0. For MGPS (Norén et al., 2008), a signal was detected if *EBGM05* value >1. To ensure the reliability of our findings, only drugs that yield positive signals at the PT level across all four methods were included in subsequent analysis. Drugs with positive signals were categorized according to the Anatomical Therapeutic Chemical (ATC) classification system and their clinical applications. Generally, higher *PRR, ROR, IC* or *EBGM* values indicate stronger signal strength. We have also conducted a brief analysis of Drug-drug interaction (DDI) to clarify their impact on AEs outcomes. This study employed the Ω shrinkage measure method to identify DDIs and screen out drug-drug combinations that increased the risk of target AE. A positive DDI signal was indicated when the lower limit of the 95%CI for Ω exceeded 0.

### 2.3 Sensitivity analysis

The gender and age, as well as the qualifications and reporters, may introduce potential bias into this study. To enhance data quality and minimise erroneous reporting, cases submitted by non-professional reporters (such as consumers) were excluded. For AEs of MRAs cases reported by healthcare professionals (such as physicians and pharmacists), a specific analysis of disproportionate distribution was again conducted. Additionally, we performed separate gender and age subgroup analyses for signals identified within the FAERS database.

### 2.4 External validation

In order to identify the false positives in the FAERS database, we extracted relevant data from the WHO VigiAccess database and conducted a concurrent disproportionality analysis. We then compared positive signals from the FAERS database with those from the WHO VigiAccess database to validate their reliability. WHO VigiAccess, a freely accessible portal for the WHO Programme for International Drug Monitoring database, provides access to drug safety reports collected by the Uppsala Monitoring Centre. The database relies on the System Organ Classification and PTs from the MedDRA Dictionary (Sultana et al., 2020). We extracted the data from the WHO VigiAccess database (https://www.vigiaccess.org) on December 29, 2024. To manage the extensive dataset, Python 3.10 was employed to call the requests package, retrieve the BASENAME of all drugs from the WHO drug dictionary, and obtain JSON data from the front-end interface. The JSON data were processed and visualized using the Pandas package and subsequently exported to Excel for comprehensive data download. Statistical analysis was performed using SAS 9.4 software.

In addition, we conduct clinical studies to validate the signals and consult with clinical experts to interpret the findings.

### 2.5 Statistical analysis

This study was performed according to the reporting of a disproportionality analysis for drug safety signal detection using individual case safety reports in pharmacovigilance (READUS-PV) (Fusaroli et al., 2024). Descriptive analysis was conducted to summarize the clinical characteristics, including patients’ demographics, outcomes, occupation of reporters and time to onset. All analyses were performed using SAS 9.4 software.

## 3 Result

### 3.1 Clinical characteristics

**FIGURE 1:**
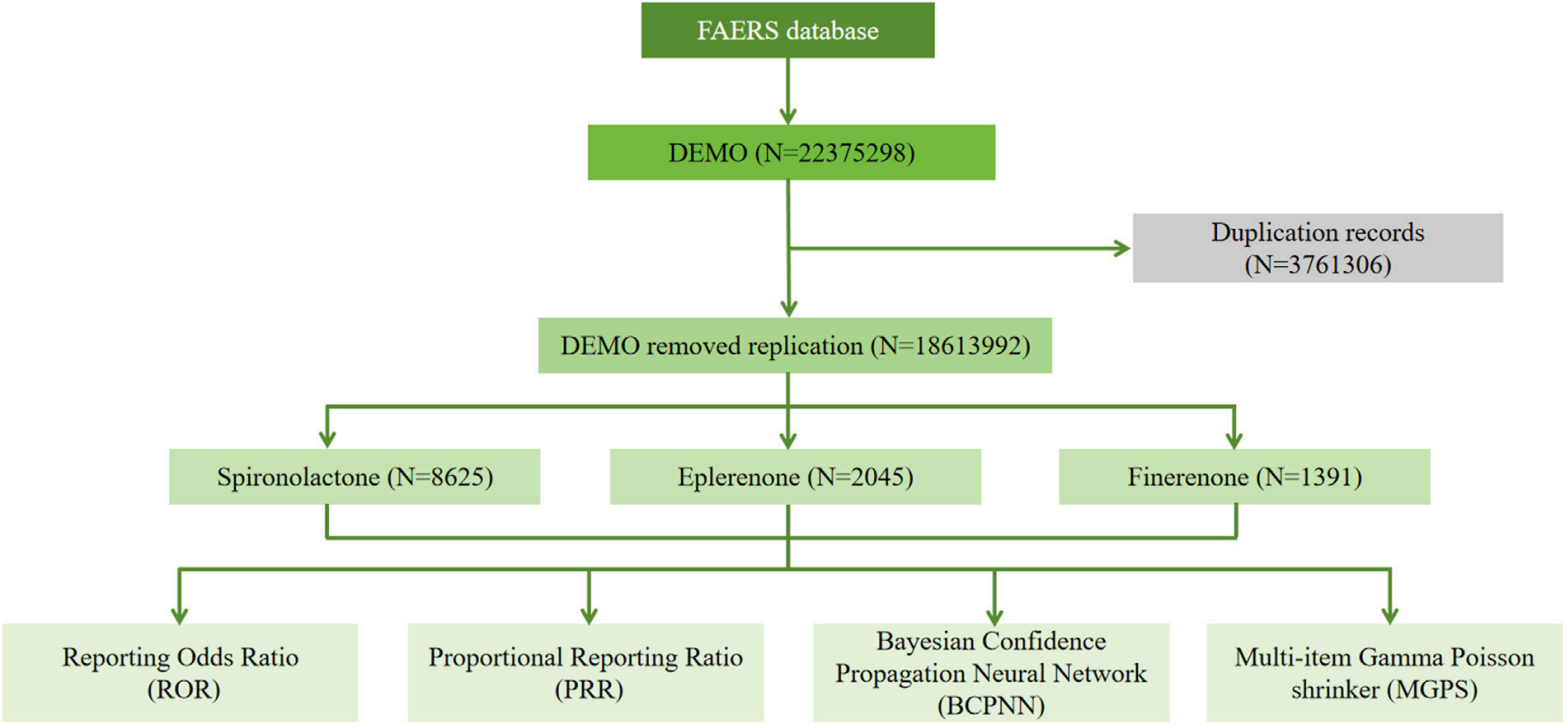
Flow diagram of this study in FAERS database. DEMO indicates patient demographic and administrative information.

From Q1 2004 to Q4 2024, the FAERS database recorded a total of 22,375,298 AEs. As shown in Supplementary Figure S1, the highest number of cases was reported were spironolactone in 2019 (968 cases), eplerenone in 2019 (218 cases), and finerenone in 2024 (509 cases). After excluding duplicates and incomplete data, 18,613,992 reports involving PS drugs were retained. Of these, 8,625 AEs were reported for spironolactone, 2,045 for eplerenone, and 1,391 for finerenone. The detailed process of data collection, interpretation, and analysis is shown in Figure 1. The demographic and clinical characteristics of these cases are summarized in Table 1. Most reports were submitted by healthcare professionals.

**TABLE 1:** Demographic characteristics and AE reports in FAERS database.

Characteristics	Spironolactone	Eplerenone	Finerenone
Total	8625	2045	1391
Gender, n (%)			
Female	4296 (49.81)	507 (24.79)	476 (34.22)
Male	3588 (41.60)	1247 (60.98)	653 (46.94)
Missing	741 (8.59)	291 (14.23)	262 (18.84)
Age (years)			
Median (IQR)	70 (58,80)	72 (61,79)	71 (64,78)
<18 (%)	131 (1.52)	3 (0.15)	0 (0.00)
18–44 (%)	695 (8.06)	84 (4.11)	15 (1.08)
45–64 (%)	1677 (19.44)	383 (18.73)	165 (11.86)
>65 (%)	4355 (50.49)	1045 (51.10)	533 (38.32)
Missing (%)	1767 (20.49)	530 (25.92)	678 (48.74)
Top 5 reported Countries			
	US [3035 (35.19)]	JP [415 (20.29)]	US [1008 (72.47)]
	FR [1709 (19.81)]	US [372 (18.19)]	JP [118 (8.48)]
	UK [827 (9.59)]	FR [276 (13.50)]	CN [47 (3.38)]
	JP [527 (6.11)]	UK [254 (12.42)]	GE [22 (1.58)]
	ES [459 (5.32)]	GE [254 (12.42)]	IL [19 (1.37)]
Reporter			
Consumer (%)	2079 (24.10)	363 (17.75)	382 (27.46)
Lawyer (%)	7 (0.08)	0	0
Not Specified (%)	256 (2.97)	59 (2.89)	1 (0.07)
Other health-professional (%)	1317 (15.27)	252 (12.32)	0
Pharmacist (%)	2329 (27.00)	547 (26.75)	467 (33.57)
Physician (%)	2637 (30.57)	824 (40.29)	541 (38.89)
Outcome			
Death (%)	491 (5.69)	129 (6.31)	109 (7.84)
Life-threatening (%)	690 (8.00)	92 (4.50)	20 (1.44)
Hospitalization (%)	3951 (45.81)	893 (43.67)	186 (13.37)
Disability (%)	202 (2.34)	53 (2.59)	4 (0.29)
Congenital anomaly (%)	42 (0.49)	4 (0.20)	0 (0.00)
Other (%)	3614 (41.90)	1099 (53.74)	630 (45.30)
Serious reports			
Serious (%)	6972 (80.83)	1795 (87.78)	881 (63.34)
Non-serious (%)	1653 (19.17)	250 (12.22)	510 (36.66)

For spironolactone, the proportion of female patients (49.81%) slightly exceeded that of male patients (41.60%). In contrast, male patients represented a higher proportion for both eplerenone (60.98%) and finerenone (46.94%) compared to females (24.79% and 34.22%, respectively). Most of the cases with age information involved patients aged 65 or older. The median age was 70 years for spironolactone, 72 years for eplerenone, and 71 years for finerenone.

**FIGURE 2:**
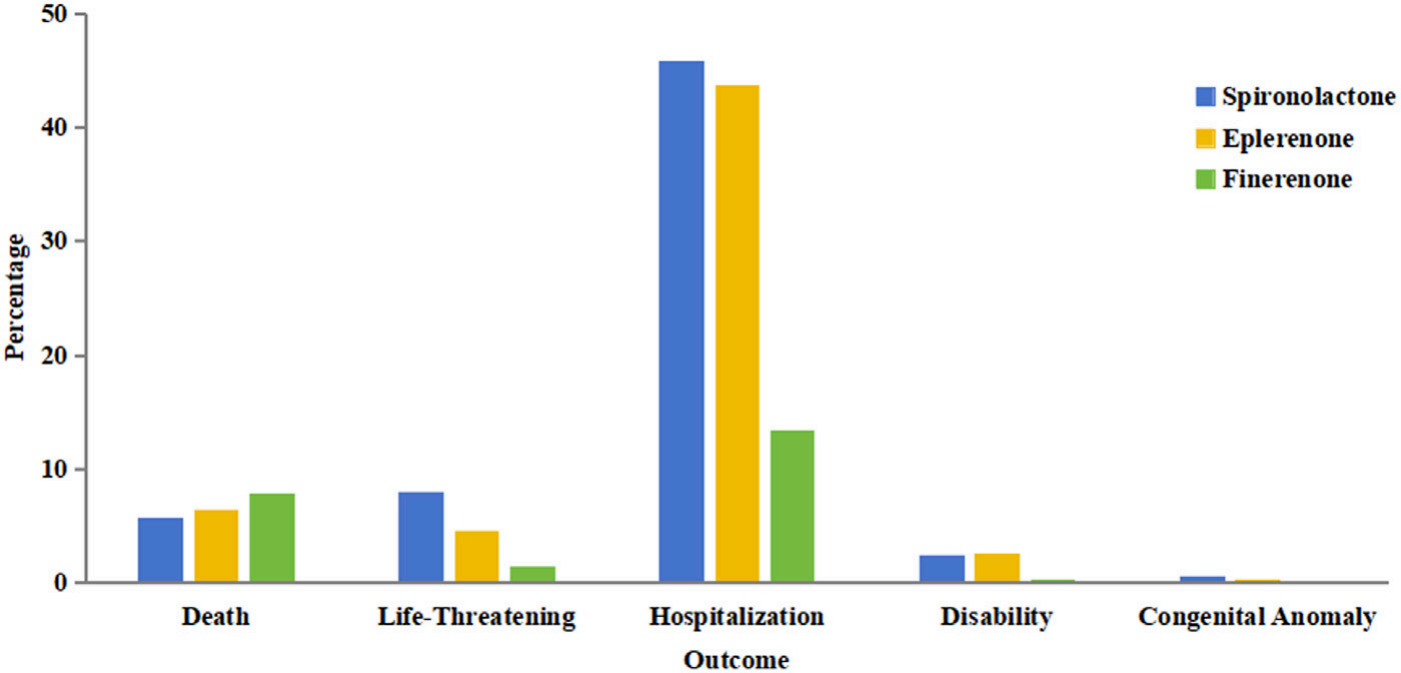
The overall outcome for the three MRAs in FAERS database.

The proportion of serious AEs was lower for finerenone (63.34%) compared to spironolactone (80.28%) and eplerenone (87.78%). Additionally, overall outcome metrics are visualized in Figure 2. Hospitalization rates were highest for all three drugs: 45.81% for spironolactone, 43.67% for eplerenone, and 13.37% for finerenone. The death rate was slightly higher for finerenone (7.84%) compared to spironolactone (5.69%) and eplerenone (6.31%). However, the life-threatening rate for finerenone (1.44%) was considerably lower than for the other two (8.00% for spironolactone and 4.50% for eplerenone). Notably, no congenital anomalies were reported for finerenone, whereas 0.49% of cases for spironolactone and 0.20% for eplerenone involved congenital anomalies.

The time to event onset, as shown in Supplementary Figure S2, revealed a median onset of 27 days (4–139) for spironolactone, 38 days (7–217) for eplerenone, and 21 days (2–60) for finerenone. For all three drugs, the majority of AEs occurred within the first 30 days of exposure.

### 3.2 Analysis of AE signals at the SOC level

**FIGURE 3:**
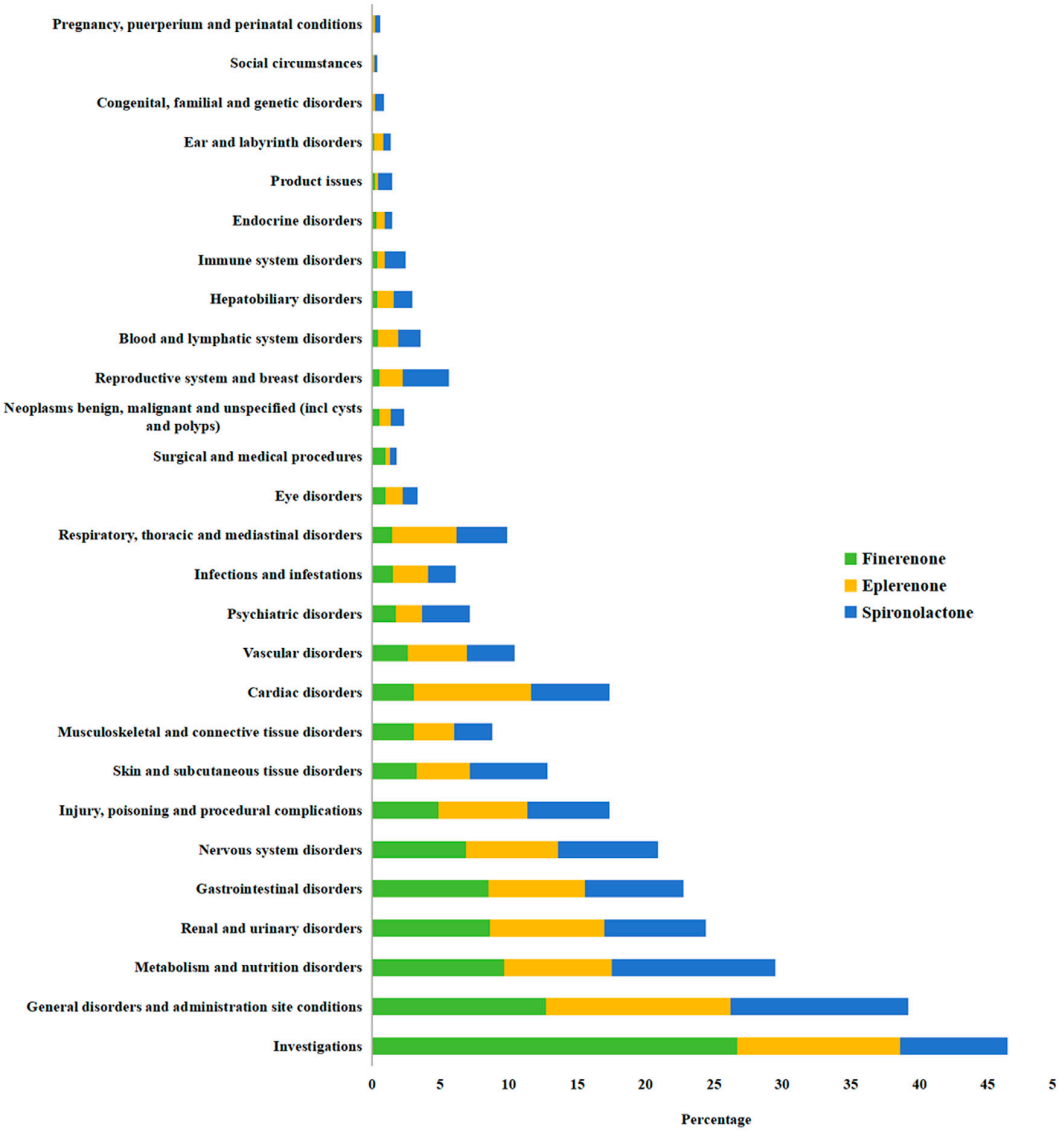
The percentage of AEs cases of the three MRAs at the SOC level in the FAERS database.

When AE signals for the three drugs were categorized by SOC based on the percentage of reported events, significant signals for spironolactone were observed in the following SOCs: metabolism and nutrition disorders (3,293 cases, 96 PTs, ROR 6.10, PRR 5.49, IC025 2.40, EBGM05 5.28), renal and urinary disorders (2,045 cases, 78 PTs, ROR 4.11, PRR 3.88, IC025 1.89, EBGM05 3.71), and reproductive system and breast disorders (927 cases, 90 PTs, ROR 3.87, PRR 3.77, IC025 1.81, EBGM05 3.53). For eplerenone, significant signals were found in renal and urinary disorders (493 cases, 42 PTs, ROR 4.70, PRR 4.39, IC025 1.99, EBGM05 4.00), metabolism and nutrition disorders (465 cases, 96 PTs, ROR 3.86, PRR 3.63, IC025 1.71, EBGM05 3.30), and cardiac disorders (507 cases, 79 PTs, ROR 3.48, PRR 3.27, IC025 1.57, EBGM05 2.98). For finerenone, the top three SOCs were investigations (661 cases, 77 PTs, ROR 5.58, PRR 4.35, IC025 1.99, EBGM05 3.98), renal and urinary disorders (214 cases, 38 PTs, ROR 4.88, PRR 4.55, IC025 1.96, EBGM05 3.95), and metabolism and nutrition disorders (239 cases, 25 PTs, ROR 4.83, PRR 4.46, IC025 1.94, EBGM05 3.90). Metabolism and nutrition disorders, and renal and urinary disorders were common to all three drugs, as shown in Table 2; Figure 3.

**TABLE 2:** Significant safety signals at the SOC level in FAERS database.

SOC	N (cases)	N (PTs)	ROR (95%CI)	PRR (χ^2^)	IC (IC025)	EBGM (EBGM05)
Spironolactone						
Metabolism and nutrition disorders	3293	96	6.10 (5.88,6.32)	5.49 (12333.9)	2.45 (2.40)	5.48 (5.28)
Renal and urinary disorders	2045	78	4.11 (3.93,4.30)	3.88 (4456.89)	1.96 (1.89)	3.88 (3.71)
Reproductive system and breast disorders	927	90	3.87 (3.63,4.13)	3.77 (1904.13)	1.91 (1.81)	3.77 (3.53)
Cardiac disorders	1562	112	2.22 (2.11,2.33)	2.15 (982.25)	1.10 (1.03)	2.15 (2.04)
Endocrine disorders	143	41	2.03 (1.72,2.39)	2.03 (74.43)	1.02 (0.77)	2.03 (1.72)
Eplerenone						
Renal and urinary disorders	493	42	4.70 (4.28,5.15)	4.39 (1314.97)	2.13 (1.99)	4.39 (4.00)
Metabolism and nutrition disorders	465	96	3.86 (3.51,4.24)	3.63 (906.35)	1.86 (1.71)	3.63 (3.30)
Cardiac disorders	507	79	3.48 (3.18,3.81)	3.27 (820.00)	1.71 (1.57)	3.27 (2.98)
Endocrine disorders	35	12	2.33 (1.67,3.25)	2.33 (26.53)	1.22 (0.68)	2.33 (1.67)
Vascular disorders	256	41	2.08 (1.84,2.36)	2.03 (137.37)	1.02 (0.83)	2.03 (1.79)
Finerenone						
Investigations	661	77	5.58 (5.10,6.10)	4.35 (1819.38)	2.12 (1.99)	4.35 (3.98)
Renal and urinary disorders	214	38	4.88 (4.25,5.62)	4.55 (603.72)	2.19 (1.96)	4.55 (3.95)
Metabolism and nutrition disorders	239	25	4.83 (4.22,5.51)	4.46 (654.74)	2.16 (1.94)	4.46 (3.90)
Endocrine disorders	8	7	1.27 (0.63,2.54)	1.27 (0.46)	0.34 (-0.66)	1.27 (0.63)
Vascular disorders	65	12	1.24 (0.97,1.58)	1.23 (2.91)	0.30 (-0.06)	1.23 (0.96)

*N(PT)*, the number of PTs included under the SOC; *N(cases)*, the number of cases resulted about the SOC; *ROR*, reporting odds ratio; *PRR*, proportional reporting ratio; *IC*, information components; *EBGM*, empirical bayesian geometric mean; *CI*, confidence interval; *χ* ^*2*^, Chi-square test; *IC025*,the lower limit of 95% CI for IC; *EBGM05*, the lower limit of 95%CI for EBGM value.

### 3.3 Analysis of AE signals at the PT level

We conducted a detailed disproportionality analysis at the PT level and ranked the results by both reporting frequency and ROR in descending order. The top 30 PTs with the highest frequency and signal intensity were further analyzed, as presented in Tables 3, 4. IC025 and EBGM05 values are provided in Supplementary Tables S3, S4.

**TABLE 3:** The top 30 PTs with the highest percentage of signal detection in FAERS database.

Spironolactone (250/2480)				Eplerenone (122/1105)				Finerenone (35/520)			
PT	N	ROR (95% Cl lower limit)	PRR (χ^2^)	PT	N	ROR (95% Cl lower limit)	PRR (χ^2^)	PT	N	ROR (95% Cl lower limit)	PRR (χ^2^)
Hyperkalaemia	1333	93.73 (88.61)	89.27 (111427)	Acute kidney injury	263	14.58 (12.88)	13.97 (3172.65)	Glomerular filtration rate decreased	179	434.80 (372.97)	403.41 (70593.5)
Acute kidney injury	1110	13.11 (12.34)	12.62 (11840.5)	Hyperkalaemia	154	47.79 (40.71)	46.57 (6837.46)	Hyperkalaemia	152	116.85 (99.13)	109.74 (16306.6)
Drug interaction	671	9.63 (8.92)	9.42 (5042.05)	Hypotension	103	5.44 (4.47)	5.36 (366.09)	Blood creatinine increased	133	52.61 (44.17)	49.84 (6357.75)
Hyponatraemia	486	19.60 (17.91)	19.28 (8349.43)	Cardiac failure*	95	12.50 (10.20)	12.31 (987.47)	Blood potassium increased	99	158.22 (129.32)	151.93 (14747.2)
Hypotension	428	4.81 (4.37)	4.75 (1268.75)	Drug interaction	81	5.38 (4.32)	5.32 (284.61)	Death*	96	2.88 (2.35)	2.81 (113.23)
Dehydration	397	6.64 (6.01)	6.56 (1867.00)	Hyponatraemia	72	13.45 (10.66)	13.30 (818.48)	Renal impairment*	73	22.79 (18.05)	22.14 (1474.29)
Drug hypersensitivity	301	3.43 (3.06)	3.40 (511.83)	Renal impairment*	59	7.56 (5.85)	7.50 (332.26)	Dizziness*	58	2.95 (2.27)	2.90 (72.76)
Gynaecomastia	295	19.90 (17.74)	19.70 (5188.53)	Renal failure*	57	4.31 (3.32)	4.28 (143.45)	Acute kidney injury*	45	5.79 (4.31)	5.70 (174.91)
Bradycardia	208	8.58 (7.48)	8.52 (1376.81)	Dehydration	52	4.05 (3.08)	4.02 (118.15)	Hypotension	38	4.78 (3.47)	4.72 (111.66)
Confusional state	188	2.58 (2.23)	2.57 (180.32)	Blood potassium increased	47	30.34 (22.76)	30.10 (1318.59)	Urine albumin/creatinine ratio increased*	35	2343.21 (1650.84)	2310.07 (73223.2)
Blood creatinine increased	184	6.19 (5.35)	6.16 (793.21)	Oedema peripheral	45	3.74 (2.79)	3.72 (89.57)	Hyponatraemia	27	12.02 (8.22)	11.89 (269.54)
Renal failure	181	2.91 (2.51)	2.90 (225.00)	Blood creatinine increased	43	6.78 (5.02)	6.74 (210.23)	Renal failure*	18	3.24 (2.04)	3.22 (27.68)
Hypokalaemia*	177	8.72 (7.52)	8.67 (1196.79)	Hypokalaemia*	43	9.92 (7.35)	9.86 (342.07)	Product prescribing issue	13	22.12 (12.82)	22.01 (260.56)
Renal impairment	175	4.77 (4.11)	4.74 (516.38)	General physical health deterioration	39	3.75 (2.74)	3.73 (78.16)	Blood pressure decreased	12	4.50 (2.55)	4.48 (32.51)
Blood potassium increased	163	22.54 (19.31)	22.42 (3299.14)	Atrial fibrillation*	38	4.06 (2.95)	4.04 (87.13)	Blood creatine increased*	10	56.16 (30.16)	55.94 (538.24)
General physical health deterioration*	146	2.99 (2.54)	2.98 (192.19)	Bradycardia*	38	7.33 (5.32)	7.29 (206.09)	Urine albumin/creatinine ratio decreased*	9	9186.70 (4225.82)	9153.28 (58450.2)
Syncope	143	3.13 (2.65)	3.12 (205.43)	Blood potassium decreased*	29	9.98 (6.93)	9.94 (232.94)	Albuminuria*	8	393.81 (195.53)	392.54 (3070.49)
Cardiac failure*	142	3.94 (3.34)	3.92 (309.07)	Syncope	29	2.97 (2.06)	2.96 (37.75)	Proteinuria*	8	10.98 (5.48)	10.95 (72.32)
Orthostatic hypotension	120	15.48 (12.93)	15.42 (1606.01)	Glomerular filtration rate decreased*	28	26.16 (18.03)	26.04 (672.34)	Blood potassium decreased*	7	5.73 (2.73)	5.72 (27.28)
Breast pain	110	22.60 (18.72)	22.52 (2237.11)	Gynaecomastia	26	8.11 (5.52)	8.08 (161.22)	Glomerular filtration rate increased*	7	232.35 (110.23)	231.69 (1591.41)
Hepatic encephalopathy	96	22.63 (18.50)	22.56 (1955.78)	Arrhythmia	25	5.38 (3.63)	5.37 (88.82)	Albumin urine present*	6	410.29 (182.81)	409.29 (2399.89)
Metabolic acidosis	93	6.70 (5.47)	6.69 (448.41)	Blood pressure decreased	25	3.93 (2.65)	3.91 (54.27)	Blood potassium abnormal	5	48.63 (20.20)	48.54 (232.28)
Product substitution issue*	92	3.55 (2.89)	3.54 (167.92)	Cardiac failure chronic*	22	55.42 (36.42)	55.22 (1164.42)	Blood pressure systolic increased*	5	6.38 (2.65)	6.37 (22.64)
Blood pressure decreased	77	2.57 (2.06)	2.57 (73.84)	Contraindicated product administered	21	7.98 (5.20)	7.95 (127.58)	Blood urea increased*	5	7.06 (2.94)	7.05 (25.95)
Pemphigoid	74	24.56 (19.52)	24.50 (1647.65)	Orthostatic hypotension	20	12.02 (7.75)	11.99 (201.19)	Glomerular filtration rate abnormal	4	74.89 (28.04)	74.77 (290.17)
Product odour abnormal*	70	15.95 (12.60)	15.91 (970.47)	Vertigo	20	3.39 (2.19)	3.39 (33.65)	Renal pain*	4	9.43 (3.54)	9.42 (30.09)
Arrhythmia	66	3.02 (2.38)	3.02 (89.12)	Alanine aminotransferase increased	19	3.19 (2.03)	3.18 (28.49)	Thirst*	4	5.63 (2.11)	5.63 (15.22)
Electrolyte imbalance	63	12.68 (9.89)	12.65 (671.75)	Pulmonary oedema*	18	4.14 (2.61)	4.13 (42.75)	Protein urine present*	4	20.67 (7.75)	20.64 (74.68)
Lactic acidosis*	63	4.55 (3.56)	4.55 (173.95)	Product prescribing error*	16	3.85 (2.35)	3.84 (33.59)	Labelled drug-drug interaction medication error	4	10.67 (4.00)	10.66 (34.99)
Breast tenderness	62	17.49 (13.62)	17.45 (953.27)	Circulatory collapse	15	8.91 (5.37)	8.89 (104.96)	Acidosis*	3	9.66 (3.11)	9.65 (23.26)

*N*, number of AEs reported; *ROR*, reporting odds ratio; *PRR*, proportional reporting ratio; *IC*, information components; *EBGM*, empirical bayesian geometric mean; *CI*, confidence interval; *χ* ^*2*^, Chi-square test; *IC025*, the lower limit of 95% CI for IC; *EBGM05*, the lower limit of 95%CI for EBGM value; *, The instruction does not mention.

**TABLE 4:** The top 30 PTs with the highest signal intensity in FAERS database.

Spironolactone (250/2480)				Eplerenone (122/1105)				Finerenone (35/520)			
PT	N	ROR (95% Cl lower limit)	PRR (χ^2^)	PT	N	ROR (95% Cl lower limit)	PRR (χ^2^)	PT	N	ROR (95% Cl lower limit)	PRR (χ^2^)
Endometriosis male*	7	13978.2 (1719.68)	13974.7 (12226.1)	Venous pressure jugular decreased*	3	879.08 (269.11)	878.63 (2404.48)	Urine albumin/creatinine ratio decreased*	9	9186.70 (4225.82)	9153.28 (58450.2)
5-Alpha-reductase deficiency*	5	1663.95 (507.78)	1663.65 (4531.78)	Labelled drug-disease interaction issue*	3	461.16 (144.66)	460.92 (1312.24)	Urine albumin/creatinine ratio increased*	35	2343.21 (1650.84)	2310.07 (73223.2)
Secondary sexual characteristics absence*	3	748.72 (198.62)	748.64 (1629.04)	Hyperaldosteronism	5	162.28 (67.01)	162.15 (787.15)	Glomerular filtration rate decreased	179	434.80 (372.97,)	403.41 (70593.5)
Blood aldosterone abnormal	3	399.32 (115.60)	399.28 (993.20)	Giardiasis*	4	128.03 (47.72)	127.95 (497.03)	Albumin urine present*	6	410.29 (182.81)	409.29 (2399.89)
Bulbospinal muscular atrophy congenital*	6	374.40 (156.54)	374.32 (1881.22)	Hyperadrenocorticism*	4	119.09 (44.41)	119.01 (462.21)	Albuminuria*	8	393.81 (195.53)	392.54 (3070.49)
Double hit lymphoma*	5	249.59 (98.50)	249.55 (1100.23)	Therapeutic drug monitoring analysis not performed*	3	101.19 (32.43)	101.14 (294.27)	Glomerular filtration rate increased*	7	232.35 (110.23)	231.69 (1591.41)
Hypocalvaria*	10	212.46 (110.69)	212.38 (1901.57)	Globulins increased*	3	87.91 (28.19)	87.86 (255.23)	Blood potassium increased	99	158.22 (129.32)	151.93 (14747.2)
Female sexual arousal disorder	8	210.21 (101.44)	210.15 (1506.61)	N-terminal prohormone brain natriuretic peptide increased*	10	84.73 (45.44)	84.59 (818.60)	Hyperkalaemia	152	116.85 (99.13)	109.74 (16306.6)
Genitalia external ambiguous*	14	182.77 (105.72)	182.68 (2317.48)	Venous pressure jugular increased*	3	81.54 (26.16)	81.50 (236.47)	Glomerular filtration rate abnormal	4	74.89 (28.04)	74.77 (290.17)
Gender dysphoria*	8	166.41 (80.89)	166.37 (1213.82)	BRASH syndrome*	7	66.75 (31.72)	66.67 (449.61)	Blood creatine increased*	10	56.16 (30.16)	55.94 (538.24)
Asthenospermia	4	135.37 (49.17)	135.35 (499.55)	Cardiac failure chronic*	22	55.42 (36.42)	55.22 (1164.42)	Blood creatinine increased	133	52.61 (44.17)	49.84 (6357.75)
Blood aldosterone increased	8	104.42 (51.29)	104.39 (778.46)	Jugular vein distension*	3	50.41 (16.20)	50.39 (144.45)	Blood potassium abnormal	5	48.63 (20.20)	48.54 (232.28)
Spur cell anaemia*	3	99.83 (31.31)	99.82 (279.51)	Hyperkalaemia	154	47.79 (40.71)	46.57 (6837.46)	Renal impairment	73	22.79 (18.05)	22.14 (1474.29)
Hyperkalaemia	1,333	93.73 (88.61)	89.27 (111427)	Scrotal oedema*	3	42.88 (13.79)	42.86 (122.09)	Product prescribing issue*	13	22.12 (12.82,)	22.01 (260.56)
Electrocardiogram T wave peaked*	16	92.10 (55.79)	92.05 (1377.50)	Cardiac amyloidosis*	4	40.21 (15.05)	40.18 (152.17)	Protein urine present*	4	20.67 (7.75)	20.64 (74.68)
Electrocardiogram T wave amplitude increased*	4	91.80 (33.69)	91.79 (343.41)	Waist circumference increased*	4	38.71 (14.50)	38.69 (146.25)	Renal function test abnormal	3	16.74 (5.39)	16.72 (44.32)
Oligospermia	4	87.77 (32.24)	87.75 (328.61)	Product monitoring error*	6	36.11 (16.19)	36.07 (203.81)	Blood pressure systolic decreased	3	16.55 (5.33)	16.53 (43.74)
Urticaria cholinergic	3	85.57 (26.94)	85.56 (240.41)	Labelled drug-drug interaction issue	3	30.64 (9.86)	30.63 (85.70)	Blood pressure diastolic increased*	3	16.35 (5.27)	16.33 (43.14)
Acute cutaneous lupus erythematosus	5	82.51 (33.73)	82.50 (386.56)	Blood potassium increased	47	30.34 (22.76)	30.10 (1318.59)	Hyponatraemia	27	12.02 (8.22)	11.89 (269.54)
Therapeutic drug monitoring analysis not performed*	11	81.37 (44.52)	81.33 (838.65)	Creatinine renal clearance decreased	11	28.73 (15.89)	28.68 (292.95)	Proteinuria*	8	10.98 (5.48)	10.95 (72.32)
Spermatogenesis abnormal	3	76.79 (24.24)	76.78 (216.08)	Arrhythmia supraventricular	3	26.97 (8.68)	26.96 (74.77)	Labelled drug-drug interaction medication error*	4	10.67 (4.00)	10.66 (34.99)
Hypoosmolar state	4	68.85 (25.41)	68.84 (258.51)	Glomerular filtration rate decreased	28	26.16 (18.03)	26.04 (672.34)	Feeling drunk*	3	9.78 (3.15)	9.77 (23.60)
Angiotensin converting enzyme inhibitor foetopathy*	3	68.07 (21.54)	68.06 (191.69)	Pulmonary arterial pressure increased*	4	24.89 (9.33)	24.88 (91.43)	Acidosis*	3	9.66 (3.11)	9.65 (23.26)
Renin increased	8	66.29 (32.77)	66.27 (497.76)	Dilatation atrial*	3	24.40 (7.85)	24.39 (67.11)	Renal pain*	4	9.43 (3.54)	9.42 (30.09)
Neonatal hyponatraemia	3	53.48 (16.99)	53.47 (150.45)	Sleep deficit	4	24.31 (9.11)	24.30 (89.12)	Flank pain*	3	8.01 (2.58)	8.00 (18.38)
Blood pressure orthostatic	4	53.24 (19.72)	53.24 (199.70)	Marasmus*	3	22.29 (7.18)	22.28 (60.83)	Blood urea increased*	5	7.06 (2.94)	7.05 (25.95)
Gastrointestinal malformation*	5	52.00 (21.40)	51.99 (243.70)	Blood pressure inadequately controlled	13	21.16 (12.27)	21.11 (248.54)	Blood pressure systolic increased*	5	6.38 (2.65)	6.37 (22.64)
Nipple pain	44	49.37 (36.60)	49.29 (2031.71)	Brain natriuretic peptide increased*	6	20.40 (9.15)	20.38 (110.35)	Acute kidney injury	45	5.79 (4.31)	5.70 (174.91)
Sinoatrial block*	13	46.28 (26.70)	46.26 (562.65)	Renal tubular acidosis*	4	20.27 (7.60)	20.25 (73.05)	Blood potassium decreased*	7	5.73 (2.73)	5.72 (27.28)
Nipple swelling	5	44.37 (18.29)	44.36 (207.33)	Ventricular arrhythmia	7	19.91 (9.48)	19.89 (125.34)	Thirst*	4	5.63 (2.11)	5.63 (15.22)

*N*, number of AEs reported; *ROR*, reporting odds ratio; *PRR*, proportional reporting ratio; *IC*, information components; *EBGM*, empirical bayesian geometric mean; *CI*, confidence interval.; *χ* ^*2*^, Chi-square test; *IC025*, the lower limit of 95% CI for IC; *EBGM05*, the lower limit of 95%CI for EBGM value; *, The instruction does not mention.

For spironolactone, the most common adverse safety signals were hyperkalaemia (1,333 cases, ROR 93.73, PRR 89.27, IC025 6.25, EBGM05 80.82), acute kidney injury (1,110 cases, ROR 13.11, PRR 12.62, IC025 3.55, EBGM05 11.81), and drug interactions (671 cases, ROR 9.63, PRR 9.42, IC025 3.10, EBGM05 8.69). The AEs with the highest ROR values included endometriosis males (7 cases, ROR 13,978.20, PRR 13,974.70, IC025 1.59, EBGM05 215.01), 5-alpha-reductase deficiency (5 cases, ROR 1,663.95, PRR 1663.65, IC025 1.13, EBGM05 277.06), and secondary sexual characteristics absence (3 cases, ROR 748.72, PRR 748.64, IC025 0.33, EBGM05 144.51). These three AEs were not listed in the drug inserts. Additional new signals not mentioned in the inserts included hypokalaemia, general physical health deterioration, cardiac failure, product substitution issues, product odour abnormalities, and lactic acidosis.

For eplerenone, the most common AE signals were acute kidney injury (263 cases, ROR 14.58, PRR 13.97, IC025 3.55, EBGM05 12.33), hyperkalaemia (154 cases, ROR 47.79, PRR 46.57, IC025 4.93, EBGM05 39.48), hypotension (103 cases, ROR 5.44, PRR 5.36, IC025 2.08, EBGM05 4.41). The AEs with the highest ROR values included venous pressure jugular decreased (3 cases, ROR 879.08, PRR 878.63, IC025 0.47, EBGM05 245.95), labelled drug-disease interaction (ROR 461.16, PRR 460.92, IC025 8.78, EBGM05 439.36), and hyperaldosteronism (5 cases, ROR 162.28, PRR 162.15, IC025 1.35, EBGM05 65.82). New signals not mentioned in the drug inserts included cardiac failure, renal impairment, renal failure, hypokalaemia, atrial fibrillation, giardiasis, bradycardia, decreased blood potassium, decreased glomerular filtration rate, cardiac failure chronic, pulmonary oedema, and product prescribing error.

For finerenone, the most common AE signals were glomerular filtration rate decreased (179 cases, ROR 434.80, PRR 403.41, IC025 6.73, EBGM05 399.94), hyperkalaemia (152 cases, ROR 116.85, PRR 109.74, IC025 5.76, EBGM05 92.64), and blood creatinine increased (133 cases, ROR 52.61, PRR 49.84, IC025 4.93, EBGM05 41.75). The highest ROR values were found for urine albumin/creatinine ratio decreased (9 cases, ROR 9,186.70, PRR 9,153.28, IC025 2.27, EBGM05 2,988.19) and urine albumin/creatinine ratio increased (35 cases, ROR 2343.21, PRR 2310.07, IC025 4.64, EBGM05 1,475.25), both of which were not mentioned in the inserts. Other notable signals included glomerular filtration rate decreased (179 cases, ROR 434.80, PRR 403.41, IC025 6.73, EBGM05 339.94). Additional new adverse signals not mentioned in the drug inserts included death, renal impairment, dizziness, acute kidney injury, urine albumin/creatinine ratio increased, renal failure, blood creatine increased, urine albumin/creatinine ratio decreased, albuminuria, proteinuria, blood potassium decreased, glomerular filtration rate increased, albumin urine present, blood pressure systolic increased, blood urea increased, renal pain, thirst, protein urine present, acidosis, blood pressure diastolic increased and feeling drunk.

Detailed data on DDIs for the three MRAs are listed in Supplementary Table S5.

### 3.4 Sensitivity analysis

Reports from healthcare professionals reports (Spironolactone = 181, Eplerenone = 101, Finerenone = 31) are included within the total FAERS reports (Spironolactone = 250, Eplerenone = 122, Finerenone = 35). Detailed signals are presented in Supplementary Table S6. Analysis indicates that the abnormal signals reported by healthcare professionals in the FAERS database largely correspond to the drugs identified in the overall reports submitted by all reporters.

To validate the reliability of the FAERS database findings, we employed the WHO VigiAccess database for comparison, with the relevant results presented Supplementary Table S7, Supplementary Table S8 and Supplementary Table S9. Its positive signals were broadly consistent with those in the FAERS database.

### 3.5 Comparison of principal characteristic of AEs

**FIGURE 4:**
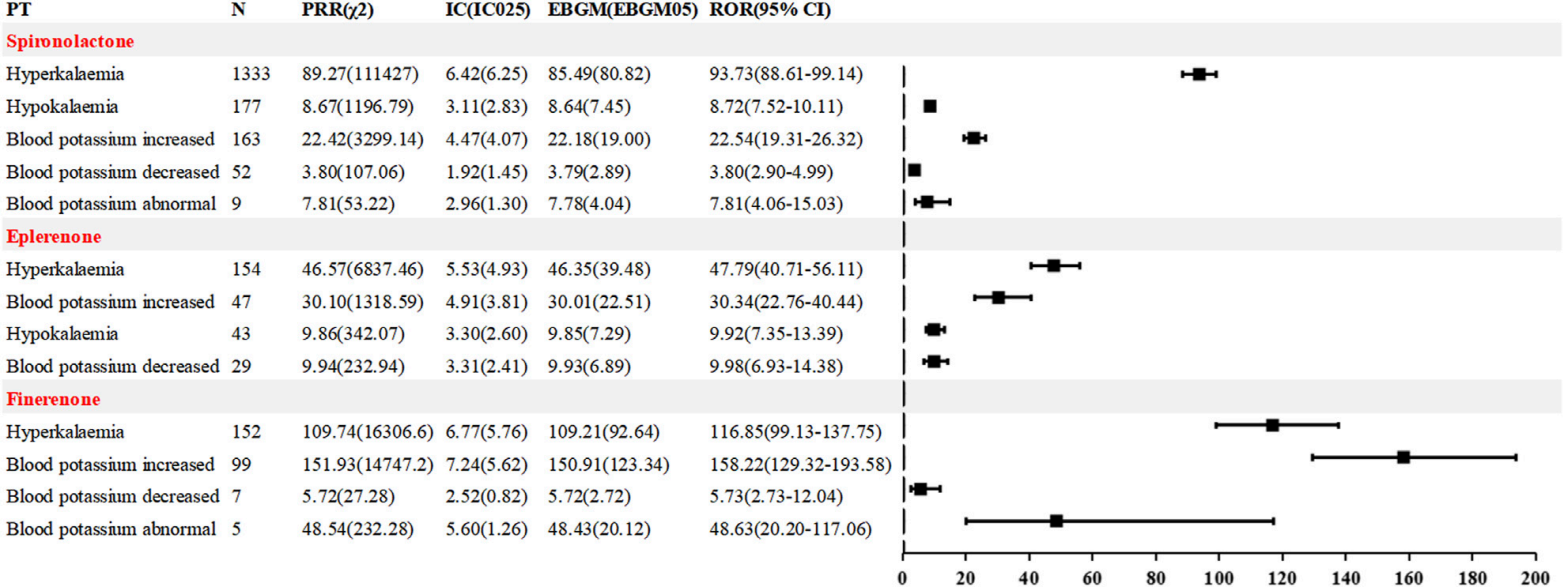
Comparison of AE signals related to blood potassium among the three MRAs in FAERS database.

Blood potassium-related AEs. Hyperkalaemia is one of the most common AEs associated with MRAs. A detailed comparative analysis of potassium-related safety signals is shown in Figure 4. Spironolactone was linked to 5 PT-level signals, involving 1,734 cases (16.60%). The most common and strongly associated PT was hyperkalaemia [1,333 cases; ROR (95% CI) 93.73 (88.61–99.14)]. Both eplerenone and finerenone were associated with 4 PTs, with 273 (13.07%) and 252 (23.20%) cases, respectively. For eplerenone, the most frequently and strongly associated PT was hyperkalaemia [154 cases, ROR (95% CI) 47.79 (40.71–56.11)]. For finerenone, hyperkalaemia was also the most common PT [152 cases, ROR (95% CI) 116.85 (99.13–137.75)], while the strongest signal was blood potassium increased [99 cases, ROR (95% CI) 158.22 (129.32–193.58)].

**FIGURE 5:**
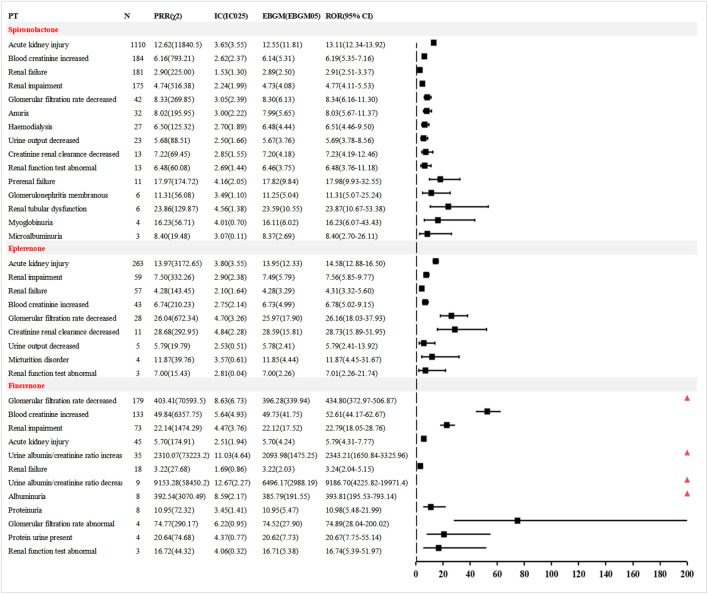
Comparison of AE signals related to renal function among the three MRAs in FAERS database.

Renal function-related AEs. Renal impairment is a serious AE frequently associated with MRAs. A comparative analysis of PT-level signals related to renal function abnormalities is presented in Figure 5. For spironolactone, 15 PTs were identified, involving 1,830 cases (17.52%). The most common event was acute kidney injury [1,110 cases; ROR (95% CI) 13.11 (12.34–13.92)], while the strongest signal was renal tubular dysfunction [6 cases; ROR (95% CI) 23.87 (10.67–53.38)]. Eplerenone was linked to 9 PTs, accounting for 473 cases (22.64%). The most frequent event was acute kidney injury [263 cases; ROR (95% CI) 14.58 (12.88–16.50)], and the strongest signal was decreased creatinine clearance [11 cases; ROR (95% CI) 28.73 (15.89–51.95)]. Finerenone was associated with 12 PTs and 519 cases (47.79%). The most common signal was decreased glomerular filtration rate [179 cases; ROR (95% CI) 434.80 (372.97–506.87)], while the strongest signal was the urine albumin/creatinine ratio decreased [9 cases, ROR (95% CI) 9,186.70 (4,225.82–19,971.4)].

**FIGURE 6:**
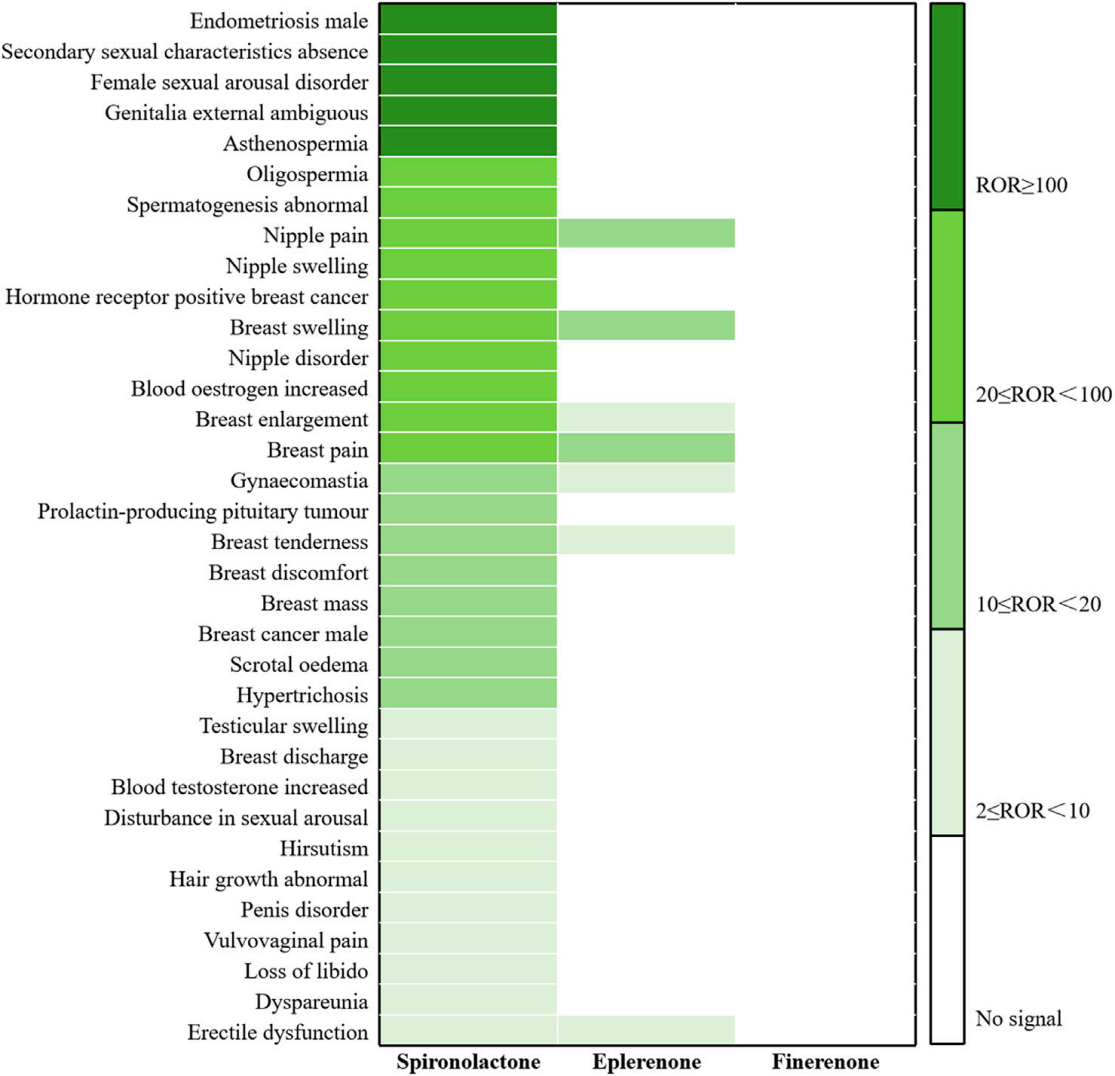
Comparison of AE signals related to sex among the three MRAs in FAERS database.

Sex hormone-related AEs. Spironolactone, a steroidal MRA, demonstrates a notable proportion of antiandrogenic effects due to its structural similarity to sex hormones. As shown in Figure 6 and Supplementary Table S10, 837 cases (8.01%) involving 34 PTs exhibited positive signals. The most frequently reported events were gynaecomastia [295 cases, ROR (95% CI) 19.90 (17.74–22.33)], breast pain [110 cases, ROR (95% CI) 22.60 (18.72–27.00)], and breast tenderness [62 cases, ROR (95% CI) 17.49 (13.62, 22.46)]. The top three PTs with the strongest signal intensity were endometriosis males (ROR 13,978.2), secondary sexual characteristics absence (ROR 748.72), and female sexual arousal disorder (ROR 210.21). Eplerenone showed fewer sex-related AEs, with 67cases (3.21%) and 7 PTs identified. The most common events were gynaecomastia [26 cases; ROR (95%CI) 8.11 (5.52–11.92)], erectile dysfunction [13 cases; ROR (95% CI) 5.36 (3.11–9.24)], and breast pain [11 cases; ROR (95% CI) 10.48 (5.80–18.95)]. The notable associations were breast swelling (ROR 18.73), nipple pain (ROR 15.43), and breast pain (ROR 10.48). No sex hormone-related AEs were reported for finerenone.

**FIGURE 7:**
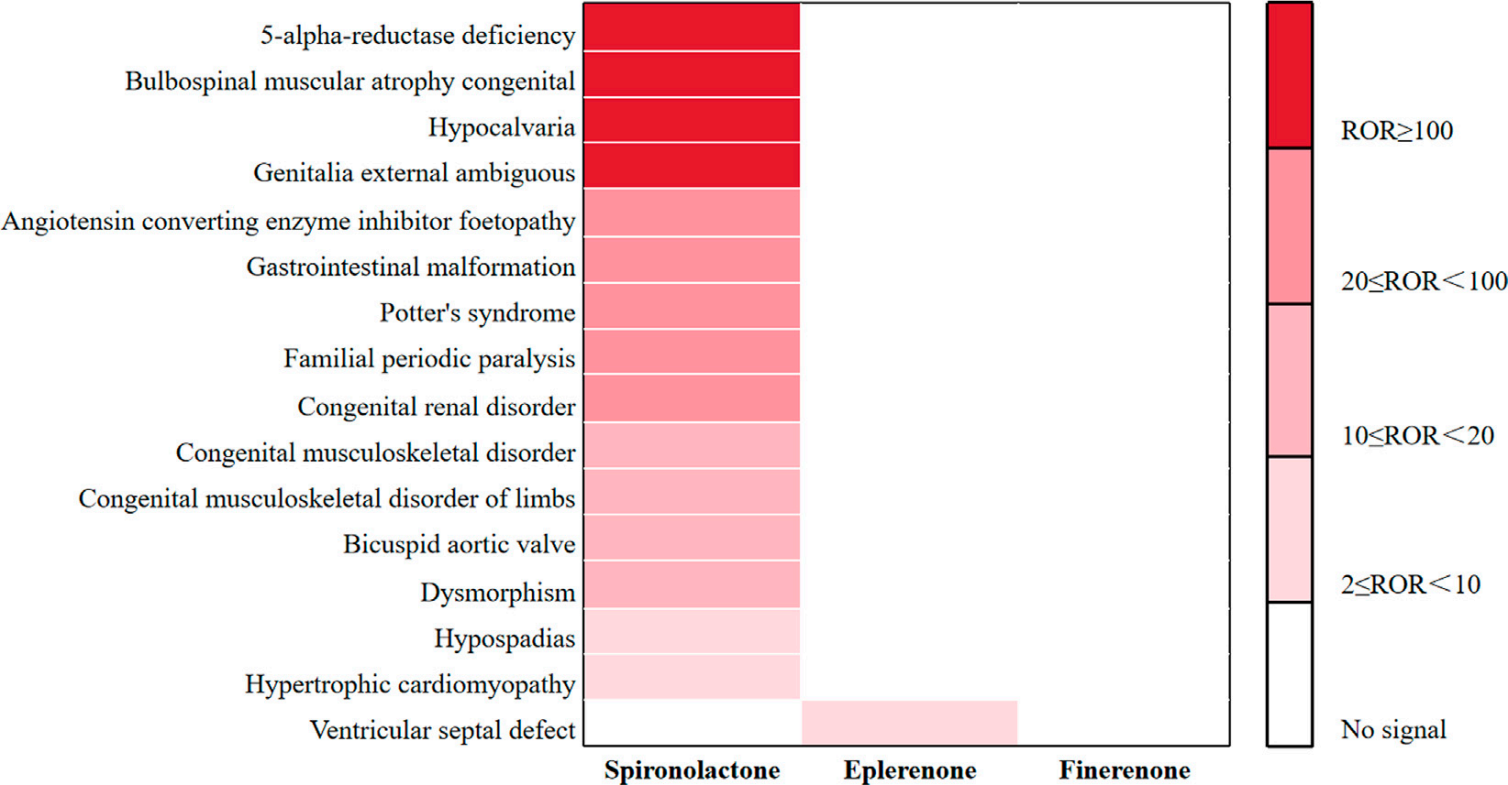
Comparison of AE signals related to congenital anomaly among the three MRAs in FAERS database.

Congenital anomaly-related AEs. We further analyzed and compared congenital anomaly-related AEs associated with the three drugs, as presented in Figure 7 and Supplementary Table S11. Spironolactone showed positive signals for 15 PTs, involving 91 cases (0.81%). The most frequently reported were genitalia externally ambiguous [14 cases; ROR (95% CI) 182.77 (105.72–315.96)], dysmorphism [13 cases; ROR (95% CI) 10.45 (6.06–18.03)], and cranial hypoplasia [10 cases; ROR (95% CI) 212.46 (110.69–407.81)]. The strongest signals were observed for 5-alpha-reductase deficiency (ROR 1,663.95), bulbospinal muscular atrophy congenital (ROR 374.40), hypocalvaria (ROR 212.46), and genitalia externally ambiguous (ROR 182.77). Eplerenone was associated with only one PT showing a signal: ventricular septal defect [4 cases, 0.19%; ROR (95% CI) 6.91 (2.59–18.42)]. No congenital anomaly-related AEs were reported for finerenone.

### 3.6 Subgroup analysis

**FIGURE 8:**
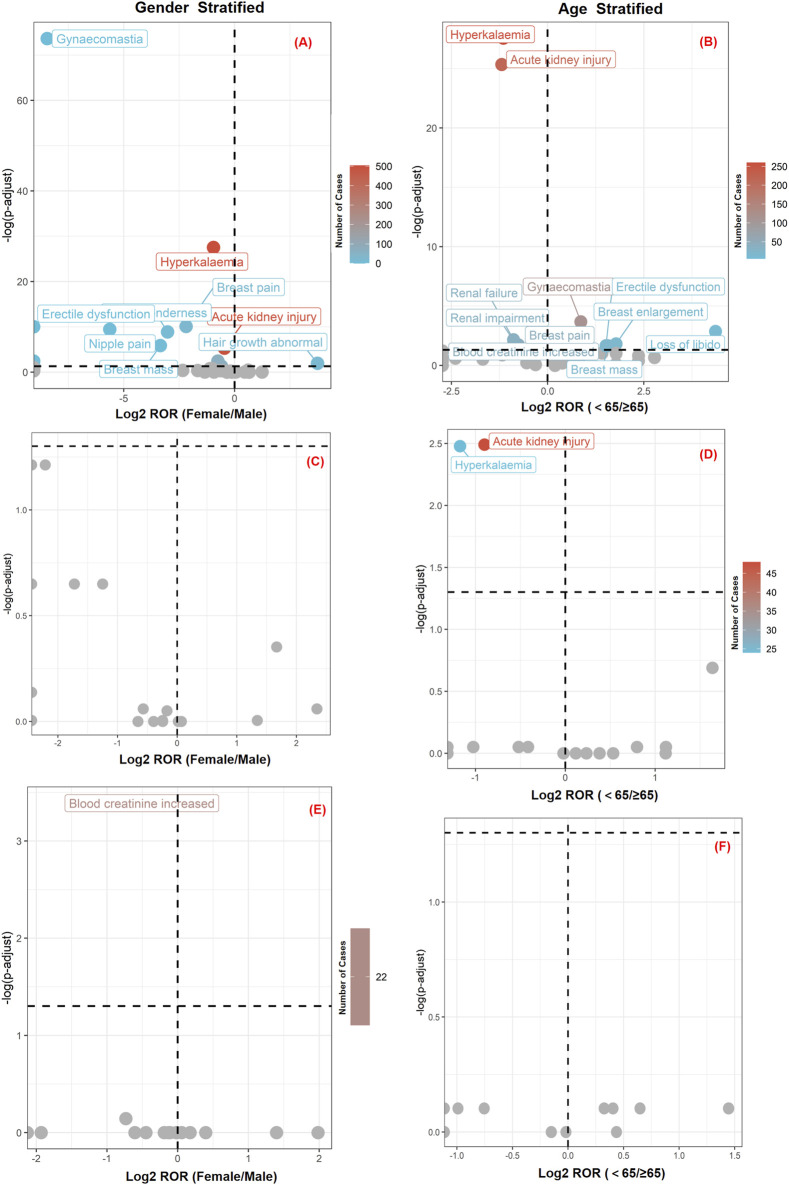
Volcano plots of sex- and age-stratified adverse event signals for three MRAs in FAERS database. Panels **(A)** and **(B)** spironolactone; **(C)** and **(D)** eplerenone; **(E)** and **(F)** finerenone. The x-axis represents log_2_ ROR and the y-axis shows −log_10_ P values from chi-square or Fisher’s exact tests. The color intensity of each data point indicates the number of reports, where warmer colors (e.g., reddish hues) correspond to a higher number of reports. Drugs positioned in the upper right and upper left quadrant exhibit both greater signal intensity and statistically significant differences. And grey dots indicate non-significant events.

To determine the relationship between patient characteristics and signals, we conducted a subgroup analysis of the FAERS database based on Gender and Age. The results are shown in Figure 8, detailed data is listed in Supplementary Table S12 and Supplementary Table S13. Spironolactone exhibited greater differential signals across both gender and age subgroups. Within the gender subgroup, gynaecomastia, hyperkalaemia, and breast pain demonstrated more pronounced differences in male patients (*p* < 0.0001). In the age subgroup, hyperkalaemia and acute kidney injury showed significant differences in elderly patients (*p* < 0.0001). However, the differences observed between eplerenone and furosemide across the various stratified analyses were not significant.

## 4 Discussion

This study performed a comprehensive pharmacovigilance analysis of spironolactone, eplerenone, and finerenone using the FAERS database from Q1 2004 to Q4 2024. The ROR and PRR were applied as primary signal detection methods, while IC and MGPS were used for signal validation. All identified AE signals were derived from real-world data and serve as valuable references for improving the safe and rational use of these drugs in clinical settings. To the best of our knowledge, this is the first study to provide a comparative real-world safety profile of all three MRAs.

### 4.1 Clinical characteristics of AE reports

The analysis showed that individuals aged over 65 years accounted for the highest proportion of AE reports for all three drugs. This aligns with the known epidemiological pattern of conditions such as CVD, CKD, and CKM (Virani et al., 2020; GBD, 2021 Diabetes Collaborators, 2023; Jacobs et al., 2014). Regarding gender distribution, finerenone and eplerenone were more frequently reported in male patients, whereas spironolactone was associated with a higher proportion of female reports. In terms of primary outcomes, spironolactone was linked to the most adverse outcomes, while finerenone was the fewest, except for death, where finerenone showed the highest rate. Several factors may explain these findings. As a steroidal MRA, spironolactone has low selectivity for the MR and can bind to other steroid hormone receptors, leading to anti-androgenic and progestogenic effects (Agarwal et al., 2021; McMullen and Van Herle, 1993). These effects are more prominent in male patients and may result in a higher rate of discontinuation or reporting. Eplerenone, in contrast, has greater receptor selectivity and a shorter half-life, which contributes to a lower incidence of AEs (Kobayashi et al., 2024). However, as a steroidal compound, it may still induce sexual side effects similar to spironolactone (Lainscak et al., 2015). Spironolactone is also commonly prescribed for acne and hormonal disorders in women, potentially explaining its higher use and reporting among female patients (Wang and Lipner, 2020). The predominance of male patients in finerenone reports may reflect the sex-specific epidemiology of type 2 diabetic nephropathy (Pitt et al., 2021; Agarwal et al., 2022), a condition for which the mortality risk is notably higher than for heart failure or hypertension alone. This may partly account for the paradox of finerenone having fewer overall AEs, yet the highest rate of reported death. Additionally, most AEs associated with the three MRAs occurred within the first month of treatment, highlighting the importance of close monitoring during the initial treatment phase.

### 4.2 SOC level analysis

At the SOC level, all three MRAs were associated with metabolism and nutritional disorders, particularly hyperkalaemia. Spironolactone and eplerenone showed a higher frequency of renal and urinary disorders. Spironolactone was more frequently linked to reproductive system and breast disorders, while eplerenone was more commonly associated with cardiac disorders. Finerenone was primarily associated with abnormal laboratory findings and showed significantly fewer renal and urinary disorders compared to the other two agents, reflecting its higher selectivity for the MR. These findings underscore the need for individualized pharmacovigilance strategies in clinical practice. In particular, enhanced monitoring may be warranted for patients receiving spironolactone due to its broader spectrum of adverse effects.

### 4.3 Signal strength and safety profile analysis

This study presents a comprehensive evaluation of the frequency and signal strength of AEs associated with MRAs. Among all observed events, hyperkalaemia emerged as a common AE across all three agents, consistent with previous literature and product labels (Pitt et al., 1999; Pitt et al., 2008; Bakris et al., 2020). Our in-depth analysis of potassium-related AEs revealed that finerenone exhibited the strongest signal; however, the wide 95% CI limited the precision of this estimate. The most prominent PT for finerenone was “blood potassium increased.” Spironolactone showed the highest number of potassium-related cases, followed by eplerenone. Three potential explanations may account for these differences. Firstly, the kidneys play a central role in potassium excretion (Morales and Palmer, 2024). Finerenone is primarily indicated for patients with type 2 diabetic nephropathy, a population characterized by impaired renal function and reduced potassium excretion, making hyperkalaemia more likely to result from the underlying disease rather than the drug itself. In contrast, spironolactone and eplerenone are more commonly used in patients with heart failure and hypertension, where renal impairment is generally less severe (Crespo-Leiro et al., 2016). Therefore, in these cases, potassium abnormalities are more likely attributable to the drugs themselves. Secondly, all three MRAs are frequently co-administered with ACE inhibitors or ARBs, which further increases the risk of hyperkalaemia. The RALES trial also demonstrated that while spironolactone is associated with hyperkalaemia, the risk of severe events is low when used at low doses (12.5–25 mg) (Pitt et al., 1999). The higher frequency of hyperkalaemia caused by spironolactone compared to eplerenone may be due to its active metabolites and longer half-life (Manolis et al., 2019). Although previous studies suggest that the overall risk of hyperkalaemia is lower with finerenone compared to spironolactone (Buonafine et al., 2018; Bakris et al., 2015; Pitt et al., 2013), the frequency remains higher than with eplerenone. The risk between eplerenone and finerenone appears similar, but the incidence is notably lower with the latter (Filippatos et al., 2016). Given the potential for hyperkalaemia to impair cardiovascular, renal, and musculoskeletal function, close monitoring of serum potassium is essential during MRA therapy. Where needed, potassium-lowering agents may be used for intervention (Gregg and Navaneethan, 2023). Notably, co-administration of MRAs and SGLT-2 inhibitors has been shown to reduce the risk of hyperkalaemia while offering additional therapeutic benefits for patients with heart failure or type 2 diabetic nephropathy (Provenzano et al., 2022).

Spironolactone and eplerenone were frequently associated with acute kidney injury, and it occurred more readily among the elderly. Finerenone, on the other hand, was primarily linked to a decreased glomerular filtration rate and increased blood creatinine. These AEs are frequently mentioned in the instruction manual. We conducted a literature search and identified two meta-analysis whose conclusions validated the reliability of the kidney injury signal (Pamporis et al., 2024; Elshahat et al., 2024). Both studies reported renal toxicity and homogenisation analysis indicated no significant differences among them. Our analysis of renal function–related AEs again identified finerenone as having the strongest signal intensity. However, similar to the findings on hyperkalaemia, this may reflect underlying disease progression rather than direct drug-induced toxicity. Notably, renal function decline associated with finerenone often occurred within the first month of treatment initiation (Huang et al., 2024), followed by stabilization or improvement, suggesting a potential renoprotective effect over time. This pattern mirrors the early renal adverse signals observed with SGLT2 inhibitors, which are now understood to confer long-term renal benefits despite transient initial declines in estimated glomerular filtration rate (eGFR) (Heerspink et al., 2020). Clinical trials such as RALES, EPHESUS, EMPHASIS-HF, and TOPCAT have similarly reported early reductions in renal function following initiation of spironolactone or eplerenone. These changes are typically non-progressive and rarely result in significant renal injury. Importantly, patients with heart failure and advanced CKD are particularly susceptible to so-called “pseudo-worsening renal function” (WRF), which may complicate continued use of steroidal MRAs (Mewton et al., 2020). Evidence from *post hoc* analyses of the HOMAGE trial suggested that early eGFR reductions induced by steroidal MRAs may in fact reflect therapeutic responses, as they were accompanied by decreases in natriuretic peptide levels, markers of renal injury, and blood pressure (Ferreira et al., 2023). Moreover, finerenone has been shown to outperform eplerenone in attenuating renal hypertrophy, reducing proteinuria, and downregulating the expression of genes associated with renal inflammation and fibrosis (Kolkhof et al., 2014).

Spironolactone and eplerenone are commonly associated with AEs such as drug-drug interactions (DDIs) and hypotension, whereas finerenone showed no prominent signals in these categories. Among the two steroidal MRAs, spironolactone exhibited a higher signal intensity for DDIs than eplerenone. Interestingly, previous studies have reported that eplerenone may pose a greater risk of DDIs than spironolactone due to its metabolism primarily via the cytochrome P450 enzyme CYP3A4 (Busti, 2015). This renders it more susceptible to interactions with CYP3A4 substrates or inhibitors, including ketoconazole, erythromycin, verapamil and fluconazole. However, our findings contradict this, suggesting a higher DDI signal for spironolactone, and this is primarily manifested in abnormal blood potassium levels caused by drug interactions. whereas the other two agents, particularly finerenone, demonstrate significantly fewer. One possible explanation is that spironolactone is frequently co-administered with renin–angiotensin system inhibitors such as ACEIs or ARBs, which collectively increase the risk of hyperkalaemia and may contribute to the higher observed incidence of DDIs. As for hypotension, both spironolactone and eplerenone demonstrated comparable signal intensities. These findings underscore the need for clinicians to carefully assess potential interactions and monitor blood pressure when prescribing these agents, particularly in combination with other antihypertensive or potassium-sparing medications.

One of the main AEs limiting the clinical use of spironolactone is its propensity to induce sex hormone–related side effects, due to its affinity for androgen and progesterone receptors. In our analysis, spironolactone was associated with positive signals for 34 sex-related AEs, several of which were novel and exhibited notably high signal intensities, raising substantial concern among both clinicians and patients. In contrast, eplerenone showed 7 positive signals in this category, most of which were of moderate or low intensity, with the strongest signal observed for breast swelling. The most frequently reported sex-related AEs for both drugs included gynaecomastia, breast pain and tenderness, and erectile dysfunction, which were more pronounced in male. Notably, no sex hormone-related AEs were reported for finerenone, reinforcing its superior receptor selectivity in real-world settings. These results are consistent with previous literature, including systematic reviews, case reports, and FDA pharmacovigilance studies (Tofte et al., 2020; Elgaafary et al., 2020; Lin et al., 2025; Mitchell et al., 2019; Elshahat et al., 2024). In the RALES trial, a significant proportion of patients in the spironolactone group (10%) discontinued treatment due to gynaecomastia and breast pain. Although eplerenone is structurally similar to spironolactone, it exhibits greater selectivity for MRs and considerably lower affinity for androgen and progesterone receptors, which helps reduce the incidence of such AEs (Moore et al., 2003; de Gasparo et al., 1987). Finerenone, as a non-steroidal MRA, has a chemical structure that differs substantially from endogenous sex hormones such as androgens and progesterone. This fundamental structural distinction likely explains the absence of sex hormone–related AEs, further confirming its favorable safety profile in this domain.

The safety signals associated with congenital anomalies were also analyzed. We found that, similar to previous results, no congenital malformations were reported for finerenone. However, Spironolactone showed positive signals for 15 PTs, with the notable associations observed for unknown external genital differentiation anomalies and cranial hypodensity, alongside corresponding signals for gastrointestinal, cardiac, and renal anomalies. Eplerenone displayed only one positive signal, a ventricular septal defect. There have been no reports of congenital malformations caused by MRAs. However, drawing from previous studies that have reported drugs with anti-androgenic properties, such as vinclozolin and flutamide, causing penile deformities in male rats (Amato et al., 2018; McIntyre et al., 2001), it is plausible that the anti-androgenic and progestogenic effects of steroidal MRAs could be involved in similar mechanisms. Nonetheless, these associations should not be interpreted as causal, and caution is advised when drawing conclusions from these findings. Recent studies highlight the importance of understanding the physiological and biochemical changes during pregnancy and the complex mechanisms underlying pregnancy-related conditions (Al-Kuraishy et al., 2023; Albogami et al., 2022).

In terms of ROR values, spironolactone was most strongly associated with endometriosis male, 5-α-reductase deficiency, and secondary sexual characteristics absence, none of which are currently listed in the drug inserts. Endometriosis male is extremely rare, typically occurring in men receiving high-dose estrogen therapy for prostate cancer (Pinkert et al., 1979; Fukunaga, 2012). Two cases were also reported in the WHO Vigiaccess, though there was no strong ROR signal. The relevant case reports of rare complications were also reported on, and it was indicated that the use of spironolactone may induce a hormonal state that could lead to endometriosis in this 52-year-old male (González et al., 2014). Lin et al. also mentioned these AEs in FDA pharmacovigilance studies (Lin et al., 2025). 5-α-reductase deficiency is an autosomal recessive disorder that impairs the conversion of testosterone to dihydrotestosterone (Okeigwe and Kuohung, 2014). The absence of secondary sexual characteristics is also indicative of sex hormone deficiency. These AEs are closely linked to hormonal imbalances and should be carefully considered when prescribing spironolactone, particularly in patients at risk of endocrine disturbance. To date, no similar signals have been reported in the literature, highlighting the need for further clinical research to elucidate the mechanisms involved and to establish appropriate prevention and management strategies. For eplerenone, the strongest signals were decreased jugular venous pressure and drug-disease interactions, both of which are noted in the drug’s specification, along with aldosteronism. These signals were primarily related to the drug’s binding to aldosterone receptors, underscoring the need for vigilant monitoring, particularly when eplerenone is co-administered with antihypertensive agents that may interact. The marked disproportionality for finerenone included decreased urine albumin/creatinine ratio, increased urine albumin/creatinine ratio, and decreased glomerular filtration rate. These effects are likely related to the underlying disease rather than the drug itself, tend to be transient, and commonly occur within the first month of treatment initiation. Nevertheless, these AEs warrant regular monitoring, although treatment discontinuation is generally not recommended unless clinically justified.

Our study placed particular emphasis on identifying newly reported AEs associated with MRAs. For spironolactone, the most frequently reported novel AEs were hypokalaemia, deterioration of systemic condition, and heart failure. In the case of eplerenone, heart failure, renal failure and hypokalaemia were most commonly observed. For finerenone, death, renal impairment and dizziness were the most frequently reported new AEs. These findings underscore the need for close monitoring of serum potassium and creatinine levels, along with careful clinical observation, to mitigate the risk of adverse outcomes potentially associated with disease progression. Additionally, our analysis revealed that eplerenone may be associated with an increased risk of atrial fibrillation and giardiasis. Although previous studies have suggested that eplerenone may reduce the burden of atrial fibrillation by remodeling upstream cardiac structures and preventing disease persistence, our findings contradict this. We hypothesise that the induction of atrial fibrillation by eplerenone is linked to its effects on AEs, including cardiac failure and electrolyte abnormalities. Cardiac failure and atrial fibrillation have a bidirectional causal relationship, and electrolyte disturbances can precipitate arrhythmias. Further validation of these hypotheses is required in clinical and mechanistic studies. Moreover, in one animal study, eplerenone treatment was found to promote the polarization of macrophages towards the M2-like phenotype (Van der Heijden and Deinum, 2018). Dysregulation of gut microbiota and M2 macrophage balance may impair intestinal homeostasis and weaken host defenses against Giardia infection (Xaplanteri et al., 2023), potentially explaining the observed signal for giardiasis.

### 4.4 Limitations

Several limitations should be considered when interpreting the findings of this study. Firstly, Although the FAERS database is an important pharmacovigilance resource, it is subject to inherent constraints, including underreporting and voluntary reporting bias. Such biases may lead to incomplete documentation of AEs, potentially impacting the accuracy and generalizability of the results. The paucity of key data in the FAERS database may introduce potential biases into our research. Secondly, as this analysis is based on retrospective observational data, and methodological limitations preclude us from establishing a causal relationship between drugs and the observed positive signals or reported cases. Thirstly, data variability and potential inaccuracies may arise due to the heterogeneous sources of reporting, such as healthcare professionals, patients, and caregivers. The deduplication process, while essential, may not fully eliminate duplicate or erroneous entries. Furthermore, prior to conducting the disproportionality analysis, this study did not exclude AE signals related to the indication, which may have led to an overestimation of these signals. Finally, there was a substantial difference in the post-marketing surveillance periods: 3-4 years for finerenone versus 20+ years for spironolactone and eplerenone. This temporal disparity introduces systematic bias, thereby potentially undermining compromises direct comparisons. Therefore, the findings should be interpreted with caution and further validated through prospective studies and clinical trials.

## 5 Conclusion

This study systematically analyzed the AEs associated with spironolactone, eplerenone, and finerenone using the FAERS data. Hyperkalaemia and renal impairment were the most frequently reported AEs for all three MRAs, particularly during the early phase of treatment, highlighting the need for early and ongoing monitoring. Older adults are particularly susceptible to acute kidney injury, which warrants particular vigilance. Spironolactone, a steroidal agent, was linked to a higher incidence of sex hormone-related AEs and congenital anomalies, warranting cautious use, especially in male and populations sensitive to hormonal imbalance. Eplerenone showed fewer hormonal AEs but was associated with unexpected signals such as atrial fibrillation and giardiasis. Finerenone exhibited the most favorable safety profile, with no significant sex hormone- or congenital anomaly-related events observed. These findings emphasize the need for personalized MRA selection and close monitoring to minimize risks and improve therapeutic outcomes.

## Data Availability

The original contributions presented in the study are included in the article/Supplementary Material, further inquiries can be directed to the corresponding author.
