# Supplementary material for: Comparative safety profiles of spironolactone, eplerenone, and finerenone: a pharmacovigilance study based on FAERS data from 2004 to 2024
Source: Front Pharmacol. 2026 Jan 7;16:1702257. doi: 10.3389/fphar.2025.1702257 (PMC12819647; doi:10.3389/fphar.2025.1702257)
Supplement: Supplementary file 1 [file Supplementaryfile1.docx]

**Supplementary material**

# Methods

Disproportionation analysis is a data mining method, which is mainly used to evaluate the correlation between drugs and adverse reactions. The core principle is to use a 2 × 2 contingency table to compare the frequency of adverse events observed in the exposed group and the non-exposed group, so as to quantify the association between drugs and adverse events. When the proportion of AEs in the exposed group exceeded that in the unexposed group, it was inferred that there was an association between drugs and specific AEs, indicating the presence of a disproportionation signal. After exceeding the threshold, the larger the signal value, the stronger the signal. In this study, we used four disproportional analysis methods: Reporting odds ratio (ROR), proportional reporting ratio (PRR), bayesian confidence propagation neural network (BCPNN), and Multi-item Gamma Poisson shrinker (MGPS).

Specific data analysis methods are listed below:

Table S1. Two-by-two contingency table for disproportionality analysis.

| **Item** | **Target adverse events reported** | **Other adverse events reported** | **Total** |
| --- | --- | --- | --- |
| **Target drugs** | a | b | a + b |
| **Other drugs** | c | d | c + d |
| **Total** | a + c | b + d | a + b + c + d |

Table S2. The principles of disproportionate measurement and the criteria for signal detection.

| Method | Calculation formula | ﻿Criteria |
| --- | --- | --- |
| ROR | $ROR=\frac{a / c}{b / d}$ | a ≥ 3  ROR ≥ 1  95%CI (lower limit) > 1 |
|  | $SE(lnROR)=\sqrt{\frac{1}{a}+\frac{1}{b}+\frac{1}{c}+\frac{1}{d}}$ |  |
|  | $95\%CI= e^{\ln\left( ROR \right)\pm1.96se}$ |  |
| PRR | $PRR=\frac{a / (a+b)}{c / (c+d)}$ | a ≥ 3  PRR ≥ 2  95%CI (lower limit) > 1 |
|  | $SE(lnPRR)=\sqrt{\frac{1}{a}-\frac{1}{a+b}+\frac{1}{c}-\frac{1}{c+d}}$ |  |
|  | $95\%CI= e^{\ln\left( PRR \right)\pm1.96se}$ |  |
|  | $\chi2 =\frac{{(ad-bc)}^{2}(a+b+c+d)}{( a+b)(a+c)(c+d)(b+d)}$ | a ≥ 3  PRR ≥ 2  $\chi2\geq4$ |
| BCPNN | IC=${log}_{2}\frac{p(x,y)}{p(x)p(y)}={log}_{2}\frac{a(a+b+c+d)}{(a+b)(a+c)}$ | IC025>0 |
|  | E(IC)=${log}_{2}\frac{(a+\gamma11)(a+b+c+d+\alpha)(a+b+c+d+\beta)}{（a+b+c+d+\gamma）(a+b+\alpha1)(a+c+\beta1)}$ |  |
|  | $V\left( IC \right)=\frac{1}{{(ln2)}^{2}}\{\left[ \frac{\left( a+b+c+d \right)-a+\gamma-\gamma11}{\left( a+\gamma11 \right)\left( 1+a+b+c+d+\gamma\right)} \right]+\left[ \frac{\left( a+b+c+d \right)-\left( a+b \right)+\alpha-\alpha1}{\left( a+b+\alpha1 \right)\left( 1+a+b+c+d+\alpha\right)} \right]+\left[ \frac{\left( a+b+c+d \right)-\left( a+c \right)+\beta-\beta1}{\left( a+c+\beta1 \right)\left( 1+a+b+c+d+\beta\right)} \right]\}$ |  |
|  | $\gamma=\gamma11\frac{(a+b+c+d+\alpha)(a+b+c+d+\beta)}{(a+b+\alpha1)(a+c+\beta1)}$ |  |
|  | *IC-2SD=E(IC)-2*$\sqrt{V(IC)}$  $\alpha1=\beta1=1；\alpha=\beta=2；\gamma11=1$ |  |
| MGPS | $EBGM=\frac{a(a+b+c+d)}{\left( a+c \right)(a+b)}$ | EBGM05>2 |
|  | $SE(lnEBGM)=\sqrt{\frac{1}{a}+\frac{1}{b}+\frac{1}{c}+\frac{1}{d}}$ |  |
|  | $95\%CI= e^{\ln\left( EBGM \right)\pm1.96se}$ |  |

**Calculatio definition of the disproportionality approach Bayesian information component:**

$$\begin{aligned} IC=\log_{2}\left( \frac{N_{\mathrm{observed}}+ 0.5}{N_{\mathrm{expected}}+ 0.5} \right)\#\left( 1 \right) \end{aligned}$$

$$\begin{aligned} N_{\mathrm{expected}}=\frac{\left( N_{\mathrm{drug}}*N_{\mathrm{effect}} \right)}{N_{\mathrm{total}}}\#\left( 2 \right) \end{aligned}$$

$$\begin{aligned} \mathrm{IC}_{025}=\log_{2}\left( \frac{N_{\mathrm{observed}}+ 0.5}{N_{\mathrm{expected}}+ 0.5} \right)-3.3*\left( N_{\mathrm{observed}}+0.5 \right)^{-\frac{1}{2}}-2*\left( N_{\mathrm{observed}}+0.5 \right)^{-\frac{3}{2}}\#\left( 3 \right) \end{aligned}$$

$$\begin{aligned} \mathrm{IC}_{975}=\log_{2}\left( \frac{N_{\mathrm{observed}}+ 0.5}{N_{\mathrm{expected}}+ 0.5} \right)+2.4*\left( N_{\mathrm{observed}}+0.5 \right)^{-\frac{1}{2}}-0.5*\left( N_{\mathrm{observed}}+0.5 \right)^{-\frac{3}{2}}\#\left( 4 \right) \end{aligned}$$

N_expected_: the number of case reports expected for the drug-ADR pairs.

N_observed_: the actual number of case reports for the drug-ADR pairs.

N_effect_: the number of case reports for the ADR, regardless of the drug.

N_total_: the total number of case reports in the database.

N_drug_: the number of case reports for the drug, regardless of the ADR.

1. **Other**

Figure S1. Annual trend of adverse event reports for three MRAs in FAERS database.


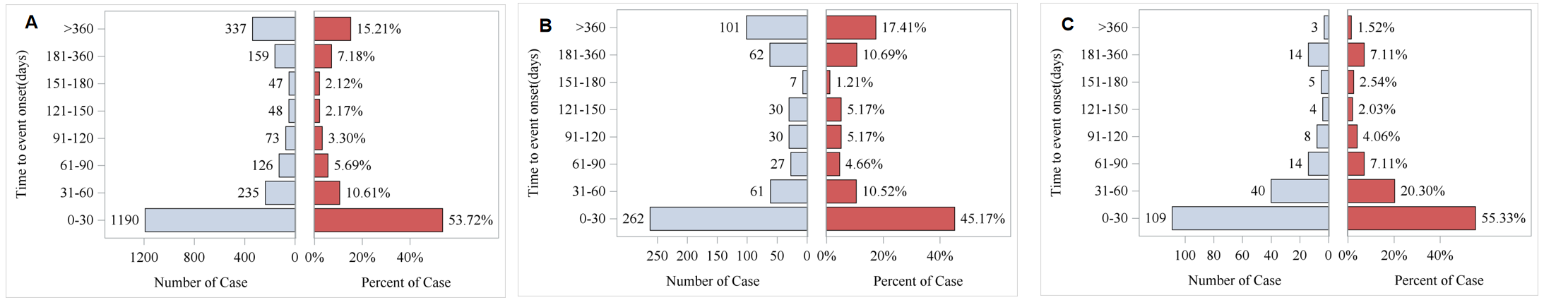


Figure S2. The number and percentage of AEs occurring at different time periods after administration of the three MRAs in the FAERS database. ( A. spironolactone; B. eplerenone; C. finerenone. )

Table S3. Top 30 PTs Ranked by Signal Detection Frequency for the Three MRAs in the FAERS Database

|  | **N** | **ROR (95%CI)** | **PRR (χ^2^)** | **IC (IC025)** | **EBGM(EBGM05)** |
| --- | --- | --- | --- | --- | --- |
| **Spironolactone（250/2480）** | | | | | |
| Hyperkalaemia | 1333 | 93.73(88.61,99.14) | 89.27(111427) | 6.42(6.25) | 85.49(80.82) |
| Acute kidney injury | 1110 | 13.11(12.34,13.92) | 12.62(11840.5) | 3.65(3.55) | 12.55(11.81) |
| Drug interaction | 671 | 9.63(8.92,10.40) | 9.42(5042.05) | 3.23(3.10) | 9.38(8.69) |
| Hyponatraemia | 486 | 19.60(17.91,21.45) | 19.28(8349.43) | 4.26(4.07) | 19.10(17.46) |
| Hypotension | 428 | 4.81(4.37,5.29) | 4.75(1268.75) | 2.25(2.09) | 4.74(4.31) |
| Dehydration | 397 | 6.64(6.01,7.33) | 6.56(1867.00) | 2.71(2.54) | 6.54(5.92) |
| Drug hypersensitivity | 301 | 3.43(3.06,3.84) | 3.40(511.83) | 1.77(1.59) | 3.40(3.03) |
| Gynaecomastia | 295 | 19.90(17.74,22.33) | 19.70(5188.53) | 4.29(4.03) | 19.52(17.39) |
| Bradycardia | 208 | 8.58(7.48,9.84) | 8.52(1376.81) | 3.09(2.83) | 8.49(7.41) |
| Confusional state | 188 | 2.58(2.23,2.98) | 2.57(180.32) | 1.36(1.14) | 2.57(2.22) |
| Blood creatinine increased | 184 | 6.19(5.35,7.16) | 6.16(793.21) | 2.62(2.37) | 6.14(5.31) |
| Renal failure | 181 | 2.91(2.51,3.37) | 2.90(225.00) | 1.53(1.30) | 2.89(2.50) |
| Hypokalaemia* | 177 | 8.72(7.52,10.11) | 8.67(1196.79) | 3.11(2.83) | 8.64(7.45) |
| Renal impairment | 175 | 4.77(4.11,5.53) | 4.74(516.38) | 2.24(1.99) | 4.73(4.08) |
| Blood potassium increased | 163 | 22.54(19.31,26.32) | 22.42(3299.14) | 4.47(4.07) | 22.18(19.00) |
| General physical health deterioration | 146 | 2.99(2.54,3.52) | 2.98(192.19) | 1.57(1.32) | 2.98(2.53) |
| Syncope | 143 | 3.13(2.65,3.69) | 3.12(205.43) | 1.64(1.38) | 3.11(2.64) |
| Cardiac failure* | 142 | 3.94(3.34,4.64) | 3.92(309.07) | 1.97(1.70) | 3.92(3.32) |
| Orthostatic hypotension | 120 | 15.48(12.93,18.53) | 15.42(1606.01) | 3.94(3.51) | 15.31(12.79) |
| Breast pain | 110 | 22.60(18.72,27.29) | 22.52(2237.11) | 4.48(3.95) | 22.28(18.45) |
| Hepatic encephalopathy | 96 | 22.63(18.50,27.68) | 22.56(1955.78) | 4.48(3.90) | 22.31(18.24) |
| Metabolic acidosis | 93 | 6.70(5.47,8.22) | 6.69(448.41) | 2.74(2.35) | 6.67(5.44) |
| Product substitution issue | 92 | 3.55(2.89,4.36) | 3.54(167.92) | 1.82(1.48) | 3.54(2.88) |
| Blood pressure decreased | 77 | 2.57(2.06,3.22) | 2.57(73.84) | 1.36(1.00) | 2.57(2.05) |
| Pemphigoid | 74 | 24.56(19.52,30.90) | 24.50(1647.65) | 4.60(3.87) | 24.21(19.25) |
| Product odour abnormal | 70 | 15.95(12.60,20.18) | 15.91(970.47) | 3.98(3.36) | 15.79(12.48) |
| Arrhythmia | 66 | 3.02(2.38,3.85) | 3.02(89.12) | 1.59(1.20) | 3.02(2.37) |
| Electrolyte imbalance | 63 | 12.68(9.89,16.24) | 12.65(671.75) | 3.65(3.05) | 12.58(9.81) |
| Lactic acidosis | 63 | 4.55(3.56,5.83) | 4.55(173.95) | 2.18(1.74) | 4.54(3.54) |
| Breast tenderness | 62 | 17.49(13.62,22.46) | 17.45(953.27) | 4.11(3.42) | 17.31(13.48) |
| **Eplerenone (122/1105)** | | | | | |
| Acute kidney injury | 263 | 14.58(12.88,16.50) | 13.97(3172.65) | 3.80(3.55) | 13.95(12.33) |
| Hyperkalaemia | 154 | 47.79(40.71,56.11) | 46.57(6837.46) | 5.53(4.93) | 46.35(39.48) |
| Hypotension* | 103 | 5.44(4.47,6.61) | 5.36(366.09) | 2.42(2.08) | 5.36(4.41) |
| Cardiac failure | 95 | 12.50(10.20,15.31) | 12.31(987.47) | 3.62(3.16) | 12.30(10.04) |
| Drug interaction | 81 | 5.38(4.32,6.70) | 5.32(284.61) | 2.41(2.02) | 5.32(4.27) |
| Hyponatraemia | 72 | 13.45(10.66,16.97) | 13.30(818.48) | 3.73(3.17) | 13.28(10.52) |
| Renal impairment* | 59 | 7.56(5.85,9.77) | 7.50(332.26) | 2.90(2.38) | 7.49(5.79) |
| Renal failure* | 57 | 4.31(3.32,5.60) | 4.28(143.45) | 2.10(1.64) | 4.28(3.29) |
| Dehydration | 52 | 4.05(3.08,5.32) | 4.02(118.15) | 2.01(1.53) | 4.02(3.06) |
| Blood potassium increased | 47 | 30.34(22.76,40.44) | 30.10(1318.59) | 4.91(3.81) | 30.01(22.51) |
| Oedema peripheral | 45 | 3.74(2.79,5.01) | 3.72(89.57) | 1.89(1.38) | 3.72(2.77) |
| Blood creatinine increased | 43 | 6.78(5.02,9.15) | 6.74(210.23) | 2.75(2.14) | 6.73(4.99) |
| Hypokalaemia* | 43 | 9.92(7.35,13.39) | 9.86(342.07) | 3.30(2.60) | 9.85(7.29) |
| General physical health deterioration | 39 | 3.75(2.74,5.14) | 3.73(78.16) | 1.90(1.35) | 3.73(2.72) |
| Atrial fibrillation* | 38 | 4.06(2.95,5.59) | 4.04(87.13) | 2.01(1.44) | 4.04(2.94) |
| Bradycardia* | 38 | 7.33(5.32,10.08) | 7.29(206.09) | 2.86(2.18) | 7.28(5.29) |
| Blood potassium decreased* | 29 | 9.98(6.93,14.38) | 9.94(232.94) | 3.31(2.41) | 9.93(6.89) |
| Syncope | 29 | 2.97(2.06,4.28) | 2.96(37.75) | 1.57(0.95) | 2.96(2.06) |
| Glomerular filtration rate decreased* | 28 | 26.16(18.03,37.93) | 26.04(672.34) | 4.70(3.26) | 25.97(17.90) |
| Gynaecomastia | 26 | 8.11(5.52,11.92) | 8.08(161.22) | 3.01(2.12) | 8.07(5.49) |
| Arrhythmia | 25 | 5.38(3.63,7.98) | 5.37(88.82) | 2.42(1.63) | 5.36(3.62) |
| Blood pressure decreased | 25 | 3.93(2.65,5.82) | 3.91(54.27) | 1.97(1.25) | 3.91(2.64) |
| Cardiac failure chronic* | 22 | 55.42(36.42,84.34) | 55.22(1164.42) | 5.78(3.43) | 54.90(36.08) |
| Contraindicated product administered | 21 | 7.98(5.20,12.25) | 7.95(127.58) | 2.99(1.98) | 7.95(5.18) |
| Orthostatic hypotension | 20 | 12.02(7.75,18.66) | 11.99(201.19) | 3.58(2.34) | 11.97(7.72) |
| Vertigo | 20 | 3.39(2.19,5.27) | 3.39(33.65) | 1.76(0.97) | 3.39(2.18) |
| Alanine aminotransferase increased | 19 | 3.19(2.03,5.01) | 3.18(28.49) | 1.67(0.87) | 3.18(2.03) |
| Pulmonary oedema* | 18 | 4.14(2.61,6.58) | 4.13(42.75) | 2.05(1.16) | 4.13(2.60) |
| Product prescribing error | 16 | 3.85(2.35,6.28) | 3.84(33.59) | 1.94(1.02) | 3.84(2.35) |
| Circulatory collapse | 15 | 8.91(5.37,14.79) | 8.89(104.96) | 3.15(1.85) | 8.88(5.35) |
| **Finerenone (35/520)** | | | | | |
| Glomerular filtration rate decreased | 179 | 434.80(372.97,506.87) | 403.41(70593.5) | 8.63(6.73) | 396.28(339.94) |
| Hyperkalaemia | 152 | 116.85(99.13,137.75) | 109.74(16306.6) | 6.77(5.76) | 109.21(92.64) |
| Blood creatinine increased | 133 | 52.61(44.17,62.67) | 49.84(6357.75) | 5.64(4.93) | 49.73(41.75) |
| Blood potassium increased | 99 | 158.22(129.32,193.58) | 151.93(14747.2) | 7.24(5.62) | 150.91(123.34) |
| Death* | 96 | 2.88(2.35,3.53) | 2.81(113.23) | 1.49(1.16) | 2.81(2.29) |
| Renal impairment | 73 | 22.79(18.05,28.76) | 22.14(1474.29) | 4.47(3.76) | 22.12(17.52) |
| Dizziness* | 58 | 2.95(2.27,3.82) | 2.90(72.76) | 1.54(1.11) | 2.90(2.23) |
| Acute kidney injury | 45 | 5.79(4.31,7.77) | 5.70(174.91) | 2.51(1.94) | 5.70(4.24) |
| Hypotension | 38 | 4.78(3.47,6.58) | 4.72(111.66) | 2.24(1.64) | 4.72(3.42) |
| Urine albumin/creatinine ratio increased* | 35 | 2343.21(1650.84,3325.96) | 2310.07(73223.2) | 11.03(4.64) | 2093.98(1475.25) |
| Hyponatraemia | 27 | 12.02(8.22,17.56) | 11.89(269.54) | 3.57(2.55) | 11.89(8.14) |
| Renal failure | 18 | 3.24(2.04,5.15) | 3.22(27.68) | 1.69(0.86) | 3.22(2.03) |
| Product prescribing issue | 13 | 22.12(12.82,38.17) | 22.01(260.56) | 4.46(2.36) | 21.99(12.75) |
| Blood pressure decreased | 12 | 4.50(2.55,7.94) | 4.48(32.51) | 2.16(1.02) | 4.48(2.54) |
| Blood creatine increased* | 10 | 56.16(30.16,104.59) | 55.94(538.24) | 5.80(2.35) | 55.80(29.96) |
| Urine albumin/creatinine ratio decreased* | 9 | 9186.70(4225.82,19971.4) | 9153.28(58450.2) | 12.67(2.27) | 6496.17(2988.19) |
| Albuminuria* | 8 | 393.81(195.53,793.14) | 392.54(3070.49) | 8.59(2.17) | 385.79(191.55) |
| Proteinuria* | 8 | 10.98(5.48,21.99) | 10.95(72.32) | 3.45(1.41) | 10.95(5.47) |
| Blood potassium decreased* | 7 | 5.73(2.73,12.04) | 5.72(27.28) | 2.52(0.82) | 5.72(2.72) |
| Glomerular filtration rate increased* | 7 | 232.35(110.23,489.75) | 231.69(1591.41) | 7.84(1.93) | 229.33(108.79) |
| Albumin urine present* | 6 | 410.29(182.81,920.82) | 409.29(2399.89) | 8.65(1.68) | 401.96(179.10) |
| Blood potassium abnormal | 5 | 48.63(20.20,117.06) | 48.54(232.28) | 5.60(1.26) | 48.43(20.12) |
| Blood pressure systolic increased* | 5 | 6.38(2.65,15.35) | 6.37(22.64) | 2.67(0.57) | 6.37(2.65) |
| Blood urea increased* | 5 | 7.06(2.94,16.98) | 7.05(25.95) | 2.82(0.63) | 7.05(2.93) |
| Glomerular filtration rate abnormal | 4 | 74.89(28.04,200.02) | 74.77(290.17) | 6.22(0.95) | 74.52(27.90) |
| Renal pain* | 4 | 9.43(3.54,25.15) | 9.42(30.09) | 3.23(0.52) | 9.41(3.53) |
| Thirst* | 4 | 5.63(2.11,15.03) | 5.63(15.22) | 2.49(0.25) | 5.63(2.11) |
| Protein urine present* | 4 | 20.67(7.75,55.14) | 20.64(74.68) | 4.37(0.77) | 20.62(7.73) |
| Labelled drug-drug interaction medication error | 4 | 10.67(4.00,28.47) | 10.66(34.99) | 3.41(0.57) | 10.65(3.99) |
| Acidosis* | 3 | 9.66(3.11,29.99) | 9.65(23.26) | 3.27(0.16) | 9.65(3.11) |

Note: *N*, number of AEs reported; *ROR*, reporting odds ratio; *PRR*, proportional reporting ratio; *IC*, information components; *EBGM*, empirical bayesian geometric mean; *CI*, confidence interval.; *χ^2^*, Chi-square test; *IC025*,the lower limit of 95% CI for IC; *EBGM05*, the lower limit of 95%CI for EBGM value; *, The instruction does not mention.

Table S4. Top 30 PTs With the Highest Signal Intensity for the Three MRAs in the FAERS Database

| **PT** | **N** | **ROR (95%CI)** | **PRR (χ^2^)** | **IC (IC025)** | **EBGM(EBGM05)** |
| --- | --- | --- | --- | --- | --- |
| **Spironolactone（250/2480）** | | | | | |
| Endometriosis male | 7 | 13978.2(1719.68,113620) | 13974.7(12226.1) | 10.77(1.59) | 1747.71(215.01) |
| 5-alpha-reductase deficiency | 5 | 1663.95(507.78,5452.60) | 1663.65(4531.78) | 9.83(1.13) | 907.90(277.06) |
| Secondary sexual characteristics absence | 3 | 748.72(198.62,2822.42) | 748.64(1629.04) | 9.09(0.33) | 544.74(144.51) |
| Blood aldosterone abnormal | 3 | 399.32(115.60,1379.44) | 399.28(993.20) | 8.38(0.40) | 332.90(96.37) |
| Bulbospinal muscular atrophy congenital | 6 | 374.40(156.54,895.49) | 374.32(1881.22) | 8.30(1.59) | 315.38(131.86) |
| Double hit lymphoma | 5 | 249.59(98.50,632.46) | 249.55(1100.23) | 7.79(1.30) | 221.93(87.58) |
| Hypocalvaria | 10 | 212.46(110.69,407.81) | 212.38(1901.57) | 7.59(2.47) | 192.06(100.06) |
| Female sexual arousal disorder | 8 | 210.21(101.44,435.59) | 210.15(1506.61) | 7.57(2.10) | 190.23(91.80) |
| Genitalia external ambiguous | 14 | 182.77(105.72,315.96) | 182.68(2317.48) | 7.39(3.01) | 167.45(96.86) |
| Gender dysphoria | 8 | 166.41(80.89,342.35) | 166.37(1213.82) | 7.26(2.09) | 153.64(74.69) |
| Asthenospermia | 4 | 135.37(49.17,372.69) | 135.35(499.55) | 6.99(0.94) | 126.82(46.06) |
| Blood aldosterone increased | 8 | 104.42(51.29,212.58) | 104.39(778.46) | 6.63(2.07) | 99.25(48.75) |
| Spur cell anaemia | 3 | 99.83(31.31,318.33) | 99.82(279.51) | 6.57(0.47) | 95.11(29.83) |
| Hyperkalaemia | 1333 | 93.73(88.61,99.14) | 89.27(111427) | 6.42(6.25) | 85.49(80.82) |
| Electrocardiogram T wave peaked | 16 | 92.10(55.79,152.05) | 92.05(1377.50) | 6.46(3.13) | 88.04(53.33) |
| Electrocardiogram T wave amplitude increased | 4 | 91.80(33.69,250.13) | 91.79(343.41) | 6.46(0.93) | 87.80(32.22) |
| Oligospermia | 4 | 87.77(32.24,238.90) | 87.75(328.61) | 6.39(0.93) | 84.10(30.90) |
| Urticaria cholinergic | 3 | 85.57(26.94,271.78) | 85.56(240.41) | 6.36(0.47) | 82.08(25.84) |
| Acute cutaneous lupus erythematosus | 5 | 82.51(33.73,201.84) | 82.50(386.56) | 6.31(1.29) | 79.26(32.40) |
| Therapeutic drug monitoring analysis not performed | 11 | 81.37(44.52,148.70) | 81.33(838.65) | 6.29(2.54) | 78.19(42.78) |
| Spermatogenesis abnormal | 3 | 76.79(24.24,243.31) | 76.78(216.08) | 6.21(0.46) | 73.98(23.35) |
| Hypoosmolar state | 4 | 68.85(25.41,186.56) | 68.84(258.51) | 6.06(0.92) | 66.58(24.57) |
| Angiotensin converting enzyme inhibitor foetopathy | 3 | 68.07(21.54,215.14) | 68.06(191.69) | 6.04(0.46) | 65.85(20.83) |
| Renin increased | 8 | 66.29(32.77,134.09) | 66.27(497.76) | 6.00(2.02) | 64.17(31.73) |
| Neonatal hyponatraemia | 3 | 53.48(16.99,168.35) | 53.47(150.45) | 5.70(0.45) | 52.11(16.55) |
| Blood pressure orthostatic | 4 | 53.24(19.72,143.73) | 53.24(199.70) | 5.70(0.90) | 51.88(19.22) |
| Gastrointestinal malformation | 5 | 52.00(21.40,126.36) | 51.99(243.70) | 5.66(1.25) | 50.69(20.86) |
| Nipple pain | 44 | 49.37(36.60,66.60) | 49.29(2031.71) | 5.59(4.12) | 48.13(35.68) |
| Sinoatrial block | 13 | 46.28(26.70,80.22) | 46.26(562.65) | 5.50(2.66) | 45.24(26.10) |
| Nipple swelling | 5 | 44.37(18.29,107.65) | 44.36(207.33) | 5.44(1.23) | 43.42(17.90) |
| **Eplerenone (122/1105)** |  |  |  |  |  |
| Venous pressure jugular decreased | 3 | 879.08(269.11,2871.57) | 878.63(2404.48) | 9.65(0.47) | 803.41(245.95) |
| Labelled drug-disease interaction issue | 3 | 461.16(144.66,1470.15) | 460.92(1312.24) | 8.78(0.50) | 439.36(137.82) |
| Hyperaldosteronism | 5 | 162.28(67.01,393.00) | 162.15(787.15) | 7.32(1.35) | 159.41(65.82) |
| Giardiasis | 4 | 128.03(47.72,343.53) | 127.95(497.03) | 6.98(0.98) | 126.24(47.05) |
| Hyperadrenocorticism | 4 | 119.09(44.41,319.39) | 119.01(462.21) | 6.88(0.97) | 117.53(43.82) |
| Therapeutic drug monitoring analysis not performed | 3 | 101.19(32.43,315.76) | 101.14(294.27) | 6.64(0.50) | 100.07(32.07) |
| Globulins increased | 3 | 87.91(28.19,274.09) | 87.86(255.23) | 6.44(0.50) | 87.06(27.92) |
| N-terminal prohormone brain natriuretic peptide increased | 10 | 84.73(45.44,157.99) | 84.59(818.60) | 6.39(2.42) | 83.84(44.96) |
| Venous pressure jugular increased | 3 | 81.54(26.16,254.13) | 81.50(236.47) | 6.34(0.50) | 80.80(25.92) |
| BRASH syndrome | 7 | 66.75(31.72,140.45) | 66.67(449.61) | 6.05(1.83) | 66.21(31.47) |
| Cardiac failure chronic | 22 | 55.42(36.42,84.34) | 55.22(1164.42) | 5.78(3.43) | 54.90(36.08) |
| Jugular vein distension | 3 | 50.41(16.20,156.83) | 50.39(144.45) | 5.65(0.47) | 50.12(16.11) |
| Hyperkalaemia | 154 | 47.79(40.71,56.11) | 46.57(6837.46) | 5.53(4.93) | 46.35(39.48) |
| Scrotal oedema | 3 | 42.88(13.79,133.34) | 42.86(122.09) | 5.42(0.45) | 42.67(13.72) |
| Cardiac amyloidosis | 4 | 40.21(15.05,107.39) | 40.18(152.17) | 5.32(0.89) | 40.01(14.98) |
| Waist circumference increased | 4 | 38.71(14.50,103.39) | 38.69(146.25) | 5.27(0.89) | 38.53(14.43) |
| Product monitoring error | 6 | 36.11(16.19,80.52) | 36.07(203.81) | 5.17(1.49) | 35.94(16.11) |
| Labelled drug-drug interaction issue | 3 | 30.64(9.86,95.22) | 30.63(85.70) | 4.93(0.42) | 30.53(9.83) |
| Blood potassium increased | 47 | 30.34(22.76,40.44) | 30.10(1318.59) | 4.91(3.81) | 30.01(22.51) |
| Creatinine renal clearance decreased | 11 | 28.73(15.89,51.95) | 28.68(292.95) | 4.84(2.28) | 28.59(15.81) |
| Arrhythmia supraventricular | 3 | 26.97(8.68,83.79) | 26.96(74.77) | 4.75(0.40) | 26.88(8.65) |
| Glomerular filtration rate decreased | 28 | 26.16(18.03,37.93) | 26.04(672.34) | 4.70(3.26) | 25.97(17.90) |
| Pulmonary arterial pressure increased | 4 | 24.89(9.33,66.43) | 24.88(91.43) | 4.63(0.81) | 24.81(9.30) |
| Dilatation atrial | 3 | 24.40(7.85,75.78) | 24.39(67.11) | 4.60(0.39) | 24.32(7.83) |
| Sleep deficit | 4 | 24.31(9.11,64.88) | 24.30(89.12) | 4.60(0.81) | 24.24(9.08) |
| Marasmus | 3 | 22.29(7.18,69.23) | 22.28(60.83) | 4.47(0.37) | 22.23(7.16) |
| Blood pressure inadequately controlled | 13 | 21.16(12.27,36.48) | 21.11(248.54) | 4.40(2.34) | 21.07(12.22) |
| Brain natriuretic peptide increased | 6 | 20.40(9.15,45.47) | 20.38(110.35) | 4.35(1.34) | 20.34(9.13) |
| Renal tubular acidosis | 4 | 20.27(7.60,54.07) | 20.25(73.05) | 4.34(0.77) | 20.21(7.58) |
| Ventricular arrhythmia | 7 | 19.91(9.48,41.82) | 19.89(125.34) | 4.31(1.54) | 19.85(9.45) |
| **Finerenone (35/520)** |  |  |  |  |  |
| Urine albumin/creatinine ratio decreased | 9 | 9186.70(4225.82,19971.4) | 9153.28(58450.2) | 12.67(2.27) | 6496.17(2988.19) |
| Urine albumin/creatinine ratio increased | 35 | 2343.21(1650.84,3325.96) | 2310.07(73223.2) | 11.03(4.64) | 2093.98(1475.25) |
| Glomerular filtration rate decreased | 179 | 434.80(372.97,506.87) | 403.41(70593.5) | 8.63(6.73) | 396.28(339.94) |
| Albumin urine present | 6 | 410.29(182.81,920.82) | 409.29(2399.89) | 8.65(1.68) | 401.96(179.10) |
| Albuminuria | 8 | 393.81(195.53,793.14) | 392.54(3070.49) | 8.59(2.17) | 385.79(191.55) |
| Glomerular filtration rate increased | 7 | 232.35(110.23,489.75) | 231.69(1591.41) | 7.84(1.93) | 229.33(108.79) |
| Blood potassium increased | 99 | 158.22(129.32,193.58) | 151.93(14747.2) | 7.24(5.62) | 150.91(123.34) |
| Hyperkalaemia | 152 | 116.85(99.13,137.75) | 109.74(16306.6) | 6.77(5.76) | 109.21(92.64) |
| Glomerular filtration rate abnormal | 4 | 74.89(28.04,200.02) | 74.77(290.17) | 6.22(0.95) | 74.52(27.90) |
| Blood creatine increased | 10 | 56.16(30.16,104.59) | 55.94(538.24) | 5.80(2.35) | 55.80(29.96) |
| Blood creatinine increased | 133 | 52.61(44.17,62.67) | 49.84(6357.75) | 5.64(4.93) | 49.73(41.75) |
| Blood potassium abnormal | 5 | 48.63(20.20,117.06) | 48.54(232.28) | 5.60(1.26) | 48.43(20.12) |
| Renal impairment | 73 | 22.79(18.05,28.76) | 22.14(1474.29) | 4.47(3.76) | 22.12(17.52) |
| Product prescribing issue | 13 | 22.12(12.82,38.17) | 22.01(260.56) | 4.46(2.36) | 21.99(12.75) |
| Protein urine present | 4 | 20.67(7.75,55.14) | 20.64(74.68) | 4.37(0.77) | 20.62(7.73) |
| Renal function test abnormal | 3 | 16.74(5.39,51.97) | 16.72(44.32) | 4.06(0.32) | 16.71(5.38) |
| Blood pressure systolic decreased | 3 | 16.55(5.33,51.37) | 16.53(43.74) | 4.05(0.31) | 16.52(5.32) |
| Blood pressure diastolic increased | 3 | 16.35(5.27,50.74) | 16.33(43.14) | 4.03(0.31) | 16.32(5.26) |
| Hyponatraemia | 27 | 12.02(8.22,17.56) | 11.89(269.54) | 3.57(2.55) | 11.89(8.14) |
| Proteinuria | 8 | 10.98(5.48,21.99) | 10.95(72.32) | 3.45(1.41) | 10.95(5.47) |
| Labelled drug-drug interaction medication error | 4 | 10.67(4.00,28.47) | 10.66(34.99) | 3.41(0.57) | 10.65(3.99) |
| Feeling drunk | 3 | 9.78(3.15,30.35) | 9.77(23.60) | 3.29(0.17) | 9.76(3.15) |
| Acidosis | 3 | 9.66(3.11,29.99) | 9.65(23.26) | 3.27(0.16) | 9.65(3.11) |
| Renal pain | 4 | 9.43(3.54,25.15) | 9.42(30.09) | 3.23(0.52) | 9.41(3.53) |
| Flank pain | 3 | 8.01(2.58,24.86) | 8.00(18.38) | 3.00(0.10) | 8.00(2.58) |
| Blood urea increased | 5 | 7.06(2.94,16.98) | 7.05(25.95) | 2.82(0.63) | 7.05(2.93) |
| Blood pressure systolic increased | 5 | 6.38(2.65,15.35) | 6.37(22.64) | 2.67(0.57) | 6.37(2.65) |
| Acute kidney injury | 45 | 5.79(4.31,7.77) | 5.70(174.91) | 2.51(1.94) | 5.70(4.24) |
| Blood potassium decreased | 7 | 5.73(2.73,12.04) | 5.72(27.28) | 2.52(0.82) | 5.72(2.72) |
| Thirst | 4 | 5.63(2.11,15.03) | 5.63(15.22) | 2.49(0.25) | 5.63(2.11) |

Note: *N*, number of AEs reported; *ROR*, reporting odds ratio; *PRR*, proportional reporting ratio; *IC*, information components; *EBGM*, empirical bayesian geometric mean; *CI*, confidence interval.; *χ^2^*, Chi-square test; *IC025*,the lower limit of 95% CI for IC; *EBGM05*, the lower limit of 95%CI for EBGM value; *, The instruction does not mention.

Table S5.The most frequently reported DDIs about three MRAs in the FAERS database.

| DrugA | DrugB | PT | n111 | Ω(95%CI) |
| --- | --- | --- | --- | --- |
| **Spironolactone** | | | | |
| POTASSIUM | SPIRONOLACTONE | Hyperkalaemia | 36 | 2.14(1.89,2.40) |
| LOSARTAN | SPIRONOLACTONE | Blood potassium increased | 33 | 1.46(1.04,1.90) |
| RAMIPRIL | SPIRONOLACTONE | Hyperkalaemia | 28 | 1.34(0.88,1.82) |
| ENALAPRIL | SPIRONOLACTONE | Hyperkalaemia | 26 | 1.98(1.49,2.47) |
| CANDESARTAN | SPIRONOLACTONE | Hyperkalaemia | 16 | 2.24(1.70,2.77) |
| SPIRONOLACTONE | SULFAMETHOXAZOLE;TRIMETHOPRIM | Hyperkalaemia | 10 | 2.08(1.52,2.63) |
| SPIRONOLACTONE | TORASEMIDE | Hyperkalaemia | 8 | 1.88(1.17,2.58) |
| SPIRONOLACTONE | VALSARTAN | Hyperkalaemia | 8 | 1.87(0.98,2.77) |
| EMPAGLIFLOZIN | SPIRONOLACTONE | Hypokalaemia | 7 | 2.46(1.46,3.46) |
| IRBESARTAN | SPIRONOLACTONE | Hyperkalaemia | 7 | 1.33(0.33,2.33) |
| Eplerenone | | | | |
| EPLERENONE | FUROSEMIDE | Acute kidney injury | 12 | 1.52(0.71,2.34) |
| EPLERENONE | FUROSEMIDE | Hyponatraemia | 8 | 2.04(1.04,3.04) |
| EPLERENONE | SACUBITRIL;VALSARTAN | Hyperkalaemia | 8 | 1.56(0.56,2.56) |
| ENALAPRIL | EPLERENONE | Cardiogenic shock | 6 | 3.69(2.53,4.84) |
| ENALAPRIL | EPLERENONE | Acute kidney injury | 5 | 2.48(1.21,3.74) |
| EPLERENONE | FUROSEMIDE | Drug interaction | 4 | 2.97(1.55,4.38) |
| EPLERENONE | RAMIPRIL | Dyspnoea | 4 | 1.6(0.19,3.02) |
| EPLERENONE | RAMIPRIL | Malaise | 4 | 1.84(0.42,3.25) |
| EPLERENONE | RAMIPRIL | Dizziness | 4 | 1.45(0.03,2.86) |
| EPLERENONE | FUROSEMIDE | Acute kidney injury | 12 | 1.52(0.471,2.34) |
| Finerenone | | | | |
| FINERENONE | INSULIN NOS | Off label use | 2 | 2.05(0.05,4.05) |
| FINERENONE | RIVAROXABAN | Iron deficiency anaemia | 2 | 2.29(0.29,4.29) |
| FINERENONE | ROSUVASTATIN | Hepatic enzyme increased | 2 | 2.25(0.25,4.25) |

TableS6. Positive signals for three MRAs extracted from the FAERS database based on reports from **healthcare professionals**.

| **PT** | **N** | **ROR (95% Cl)** | **PRR (χ^2^)** | **IC (IC025)** | **EBGM (EBGM05)** |
| --- | --- | --- | --- | --- | --- |
| **Spironolactone** | | | | | |
| Hyperkalaemia | 1273 | 75.57(71.29,80.09) | 70.63(83198.1) | 6.07(5.91) | 67.23(63.43) |
| Acute kidney injury | 467 | 12.16(11.42,12.94) | 11.54(10152.9) | 3.52(3.41) | 11.46(10.76) |
| Drug interaction | 639 | 8.67(8.01,9.38) | 8.41(4163.37) | 3.06(2.93) | 8.37(7.73) |
| Hyponatraemia | 347 | 16.09(14.67,17.65) | 15.72(6375.67) | 3.96(3.78) | 15.56(14.18) |
| Hypotension | 358 | 4.30(3.87,4.78) | 4.24(887.22) | 2.08(1.91) | 4.23(3.81) |
| Dehydration | 245 | 7.65(6.88,8.51) | 7.53(1960.08) | 2.91(2.72) | 7.50(6.74) |
| Drug hypersensitivity | 253 | 3.24(2.86,3.67) | 3.21(385.21) | 1.68(1.48) | 3.20(2.83) |
| Fall | 182 | 2.60(2.29,2.95) | 2.58(237.28) | 1.36(1.17) | 2.57(2.27) |
| Bradycardia | 196 | 7.02(6.09,8.08) | 6.95(995.68) | 2.79(2.54) | 6.92(6.01) |
| Gynaecomastia | 162 | 55.05(47.44,63.89) | 54.54(9203.02) | 5.71(5.14) | 52.50(45.24) |
| Hypokalaemia | 162 | 6.86(5.88,8.01) | 6.81(800.68) | 2.76(2.48) | 6.79(5.81) |
| Renal failure | 146 | 3.71(3.17,4.33) | 3.68(316.65) | 1.88(1.63) | 3.68(3.15) |
| Blood creatinine increased | 155 | 5.26(4.49,6.16) | 5.22(527.77) | 2.38(2.11) | 5.21(4.44) |
| Renal impairment | 138 | 4.24(3.60,4.99) | 4.22(357.84) | 2.07(1.80) | 4.21(3.57) |
| Confusional state | 138 | 2.42(2.05,2.86) | 2.41(114.10) | 1.27(1.01) | 2.41(2.04) |
| General physical health deterioration | 116 | 3.15(2.66,3.73) | 3.14(200.76) | 1.65(1.38) | 3.13(2.65) |
| Syncope | 117 | 3.02(2.51,3.62) | 3.00(156.42) | 1.58(1.29) | 3.00(2.50) |
| Cardiac failure | 110 | 3.38(2.82,4.06) | 3.37(192.96) | 1.75(1.45) | 3.36(2.80) |
| Orthostatic hypotension | 111 | 13.46(11.16,16.24) | 13.39(1261.16) | 3.73(3.31) | 13.27(11.00) |
| Blood potassium increased | 94 | 20.37(16.87,24.61) | 20.26(1985.74) | 4.32(3.82) | 19.98(16.55) |
| Atrial fibrillation | 95 | 2.60(2.13,3.18) | 2.59(92.93) | 1.37(1.05) | 2.59(2.12) |
| Hepatic encephalopathy | 71 | 18.97(15.47,23.27) | 18.89(1571.08) | 4.22(3.68) | 18.64(15.20) |
| Metabolic acidosis | 90 | 5.27(4.29,6.49) | 5.25(309.01) | 2.39(2.02) | 5.24(4.26) |
| Pemphigoid | 59 | 19.07(15.08,24.12) | 19.01(1194.87) | 4.23(3.57) | 18.76(14.84) |
| Chronic kidney disease | 65 | 5.07(3.97,6.47) | 5.06(210.87) | 2.33(1.89) | 5.04(3.95) |
| Lactic acidosis | 54 | 3.34(2.59,4.32) | 3.33(96.25) | 1.73(1.31) | 3.33(2.58) |
| Inappropriate antidiuretic hormone secretion | 56 | 10.30(7.92,13.41) | 10.28(465.56) | 3.35(2.75) | 10.21(7.84) |
| Electrolyte imbalance | 45 | 10.63(8.13,13.89) | 10.60(465.95) | 3.40(2.77) | 10.53(8.05) |
| Contraindicated product administered | 53 | 4.34(3.31,5.69) | 4.33(135.54) | 2.11(1.63) | 4.32(3.30) |
| Arrhythmia | 256 | 2.78(2.08,3.73) | 2.78(51.20) | 1.47(0.99) | 2.78(2.07) |
| **Eplerenone** | | | | | |
| Acute kidney injury | 94 | 12.68(11.18,14.39) | 12.01(2590.77) | 3.58(3.34) | 11.99(10.56) |
| Hyperkalaemia | 140 | 33.15(28.00,39.25) | 32.14(4205.23) | 5.00(4.46) | 31.97(27.01) |
| Hypotension | 72 | 4.89(3.98,6.00) | 4.80(284.24) | 2.26(1.91) | 4.80(3.91) |
| Cardiac failure | 85 | 10.86(8.76,13.46) | 10.67(744.87) | 3.41(2.94) | 10.65(8.59) |
| Drug interaction | 56 | 4.13(3.27,5.21) | 4.08(167.82) | 2.03(1.63) | 4.08(3.23) |
| Hyponatraemia | 65 | 9.50(7.44,12.14) | 9.38(486.39) | 3.23(2.70) | 9.36(7.33) |
| Renal impairment | 46 | 7.06(5.42,9.19) | 6.98(287.35) | 2.80(2.27) | 6.98(5.36) |
| Renal failure | 54 | 5.36(4.10,7.01) | 5.30(188.83) | 2.41(1.91) | 5.30(4.05) |
| Dehydration | 40 | 4.33(3.24,5.80) | 4.30(116.71) | 2.10(1.58) | 4.30(3.21) |
| Blood potassium increased | 41 | 32.67(24.00,44.47) | 32.38(1240.40) | 5.01(3.76) | 32.21(23.66) |
| Hypokalaemia | 37 | 7.31(5.35,9.98) | 7.25(215.62) | 2.86(2.20) | 7.24(5.31) |
| Bradycardia | 37 | 5.70(4.12,7.87) | 5.66(141.92) | 2.50(1.86) | 5.65(4.09) |
| General physical health deterioration | 36 | 3.65(2.64,5.05) | 3.63(70.68) | 1.86(1.29) | 3.63(2.63) |
| Atrial fibrillation | 36 | 4.27(3.08,5.93) | 4.25(89.43) | 2.09(1.49) | 4.24(3.06) |
| Oedema peripheral | 27 | 3.42(2.46,4.75) | 3.40(61.17) | 1.77(1.20) | 3.40(2.45) |
| Blood creatinine increased | 34 | 4.97(3.55,6.97) | 4.94(106.93) | 2.30(1.66) | 4.94(3.52) |
| Syncope | 22 | 3.01(2.06,4.39) | 2.99(35.93) | 1.58(0.94) | 2.99(2.05) |
| Glomerular filtration rate decreased | 23 | 20.21(13.40,30.46) | 20.11(416.32) | 4.33(2.89) | 20.04(13.30) |
| Cardiac failure chronic | 19 | 46.32(30.42,70.54) | 46.10(963.33) | 5.52(3.35) | 45.75(30.05) |
| Gynaecomastia | 20 | 25.16(16.20,39.07) | 25.05(459.92) | 4.64(2.91) | 24.95(16.06) |
| Blood potassium decreased | 19 | 9.08(5.79,14.26) | 9.05(135.90) | 3.18(2.04) | 9.04(5.76) |
| Blood pressure decreased | 19 | 3.72(2.37,5.84) | 3.71(37.59) | 1.89(1.06) | 3.71(2.36) |
| Contraindicated product administered | 15 | 6.73(4.29,10.57) | 6.71(92.22) | 2.74(1.74) | 6.70(4.27) |
| Orthostatic hypotension | 18 | 9.35(5.89,14.87) | 9.32(133.55) | 3.22(2.03) | 9.31(5.86) |
| Ventricular tachycardia | 14 | 7.70(4.64,12.78) | 7.67(87.01) | 2.94(1.71) | 7.67(4.62) |
| Vertigo | 15 | 3.64(2.19,6.05) | 3.63(28.65) | 1.86(0.92) | 3.63(2.19) |
| Arrhythmia | 14 | 3.74(2.22,6.33) | 3.74(28.06) | 1.90(0.91) | 3.73(2.21) |
| Pulmonary oedema | 14 | 3.56(2.11,6.02) | 3.55(25.70) | 1.83(0.86) | 3.55(2.10) |
| Product prescribing error |  | 4.76(2.82,8.05) | 4.75(41.47) | 2.25(1.18) | 4.75(2.81) |
| Gout | 13 | 10.75(6.23,18.53) | 10.72(114.40) | 3.42(1.89) | 10.70(6.21) |
| **Finerenone** | | | | | |
| Glomerular filtration rate decreased | 150 | 383.15(323.47,453.83) | 349.43(50984.4) | 8.42(6.47) | 341.78(288.55) |
| Hyperkalaemia | 84 | 92.21(77.58,109.60) | 84.65(11603.1) | 6.40(5.48) | 84.19(70.83) |
| Blood creatinine increased | 121 | 49.53(41.16,59.60) | 46.07(5328.20) | 5.52(4.80) | 45.94(38.18) |
| Blood potassium increased | 44 | 183.48(147.17,228.75) | 174.46(14331.0) | 7.43(5.51) | 172.54(138.40) |
| Renal impairment | 60 | 20.25(15.65,26.21) | 19.57(1057.93) | 4.29(3.53) | 19.55(15.11) |
| Acute kidney injury | 33 | 5.50(4.08,7.43) | 5.39(157.95) | 2.43(1.86) | 5.39(3.99) |
| Dizziness | 37 | 3.38(2.44,4.68) | 3.33(60.65) | 1.73(1.17) | 3.33(2.40) |
| Urine albumin/creatinine ratio increased | 26 | 2063.29(1431.12,2974.70) | 2023.25(59032.5) | 10.81(4.53) | 1790.73(1242.07) |
| Hypotension | 29 | 3.92(2.72,5.66) | 3.87(62.09) | 1.95(1.29) | 3.87(2.68) |
| Hyponatraemia | 8 | 9.93(6.74,14.64) | 9.80(205.60) | 3.29(2.32) | 9.79(6.65) |
| Urine albumin/creatinine ratio decreased | 9 | 6404.64(2944.74,13929.8) | 6370.74(40677.8) | 12.14(2.27) | 4521.46(2078.89) |
| Blood creatine increased | 8 | 49.71(24.79,99.67) | 49.48(378.81) | 5.62(1.99) | 49.32(24.60) |
| Blood pressure decreased | 8 | 4.10(2.04,8.20) | 4.08(18.63) | 2.03(0.64) | 4.08(2.04) |
| Proteinuria | 7 | 10.36(5.17,20.76) | 10.32(67.33) | 3.37(1.38) | 10.31(5.15) |
| Chronic kidney disease | 8 | 7.05(3.52,14.12) | 7.02(41.29) | 2.81(1.11) | 7.02(3.50) |
| Albuminuria | 5 | 335.77(158.57,710.98) | 334.39(2277.85) | 8.35(1.93) | 327.38(154.61) |
| Albumin urine present | 6 | 464.20(205.81,1046.98) | 462.56(2683.69) | 8.81(1.68) | 449.25(199.18) |
| Glomerular filtration rate increased | 4 | 201.27(83.20,486.92) | 200.68(980.81) | 7.63(1.36) | 198.14(81.90) |
| Product prescribing issue | 5 | 16.86(7.00,40.57) | 16.81(74.28) | 4.07(1.03) | 16.79(6.98) |
| Blood pressure systolic increased | 4 | 5.45(2.04,14.53) | 5.43(14.48) | 2.44(0.23) | 5.43(2.04) |
| Blood sodium decreased | 4 | 7.35(2.76,19.62) | 7.34(21.90) | 2.87(0.40) | 7.34(2.75) |
| Blood urea increased | 3 | 5.47(2.05,14.61) | 5.46(14.59) | 2.45(0.24) | 5.46(2.05) |
| Renal pain | 4 | 21.66(8.11,57.81) | 21.61(78.51) | 4.43(0.78) | 21.58(8.08) |
| Acidosis | 3 | 8.44(2.72,26.19) | 8.42(19.62) | 3.07(0.12) | 8.42(2.71) |
| Blood pressure diastolic increased | 3 | 17.21(5.54,53.45) | 17.18(45.68) | 4.10(0.32) | 17.16(5.53) |
| Dermatitis allergic | 3 | 7.05(2.27,21.87) | 7.03(15.53) | 2.81(0.04) | 7.03(2.27) |
| Gastric cancer | 3 | 6.90(2.22,21.42) | 6.89(15.10) | 2.78(0.03) | 6.89(2.22) |
| Glomerular filtration rate abnormal | 3 | 80.00(25.70,249.02) | 79.86(232.43) | 6.31(0.50) | 79.46(25.53) |
| Glycosylated haemoglobin increased | 3 | 7.04(2.27,21.86) | 7.03(15.52) | 2.81(0.04) | 7.03(2.26) |
| Dialysis |  | 7.00(2.25,21.73) | 6.99(15.40) | 2.80(0.04) | 6.99(2.25) |

Table S7. Validation of the Top Five SOC-Level Signals Identified in FAERS using the WHO VigiAccess Database

| **SOC** | **N(cases)** | | **ROR (95%CI)** | | **PRR (χ^2^)** | | | | **IC (IC025)** | | **EBGM (EBGM05)** |
| --- | --- | --- | --- | --- | --- | --- | --- | --- | --- | --- | --- |
| **Spironolactone** |  | |  | |  | | | |  | |  |
| Metabolism and nutrition disorders  Renal and urinary disorders  Reproductive system and breast disorders  Cardiac disorders  Endocrine disorders | 11164  5043  4561  3031  200 | | 10.54(10.33,10.76)  4.81(4.68,4.95)  6.53(6.33,6.72)  1.87(1.81,1.94)  1.43(1.25,1.64) | | 9.01(80505.9)  4.54(14088.6)  6.16(19869.7)  1.84(1182.21)  1.43(25.82) | | | | 3.16(3.13)  2.18(2.14)  2.62(2.57)  0.88(0.82)  0.51(0.31) | | 8.97(8.79)  4.53(4.40)  6.14(5.96)  1.84(1.77)  1.43(1.24) |
| **Eplerenone** |  |  | |  | |  | |  | | | |
| Renal and urinary disorders  Metabolism and nutrition disorders  Cardiac disorders  Endocrine disorders  Vascular disorders | 482  604  624  29  342 | 4.17(3.80,4.57)  4.75(4.37,5.16)  3.69(3.40,4.00)  1.90(1.32,2.74)  2.17(1.94,2.41) | | 3.97(1086.73)  4.45(1643.55)  3.47(1120.91)  1.90(12.38)  2.11(204.77) | | | 1.99(1.84)  2.15(2.02)  1.79(1.67)  0.93(0.35)  1.08(0.91) | | | 3.97(3.62)  4.45(4.09)  3.47(3.19)  1.90(1.32)  2.11(1.90) | |
| **Finerenone** |  |  | |  | | |  | | |  | |
| Investigations  Renal and urinary disorders  Metabolism and nutrition disorders  Endocrine disorders  Vascular disorders | 678  166  379  7  78 | 5.60(5.12,6.11)  4.42(3.77,5.17)  9.92(8.89,11.07)  1.41(0.67,2.95)  1.49(1.19,1.87) | | 4.34(1858.46)  4.19(409.38)  8.56(2575.04)  1.41(0.82)  1.483(12.29) | | | 2.12(1.98)  2.07(1.81)  3.10(2.91)  0.49(-0.60)  0.56(0.22) | | | 4.34(3.97)  4.19(3.58)  8.56(7.67)  1.41(0.67)  1.48(1.18) | |

*N(PT)*, the number of PTs included under the SOC; *N(cases)*, the number of cases resulted about the SOC; *ROR*, reporting odds ratio; *PRR*, proportional reporting ratio; *IC*, information components; *EBGM*, empirical bayesian geometric mean; *CI*, confidence interval.; *χ^2^*, Chi-square test; *IC025*,the lower limit of 95% CI for IC; *EBGM05*, the lower limit of 95%CI for EBGM value.

TableS8. Validation of the Top 30 PTs With the Highest Signal-Detection Frequencies in FAERS Using the WHO VigiAccess Database

| **Spironolactone** | | | | | |
| --- | --- | --- | --- | --- | --- |
| **PT** | **N** | **ROR (95% Cl)** | **PRR (χ^2^)** | **IC (IC025)** | **EBGM (EBGM05)** |
| Hyperkalaemia | 6201 | 204.96(199.43,210.65) | 186.79(1032636) | 7.40(7.32) | 168.34(163.79) |
| Acute kidney injury | 2566 | 14.94(14.36,15.54) | 14.43(31873.3) | 3.84(3.77) | 14.31(13.76) |
| Drug interaction | 428 | 4.30(3.91,4.72) | 4.28(1072.95) | 2.09(1.94) | 4.27(3.88) |
| Hyponatraemia | 1963 | 31.50(30.10,32.95) | 30.64(55327.0) | 4.90(4.83) | 30.11(28.78) |
| Hypotension | 1186 | 4.80(4.54,5.09) | 4.74(3502.82) | 2.24(2.14) | 4.73(4.47) |
| Dehydration | 704 | 7.24(6.72,7.80) | 7.18(3734.42) | 2.84(2.72) | 7.15(6.64) |
| Drug hypersensitivity | 455 | 2.70(2.46,2.96) | 2.69(482.96) | 1.43(1.28) | 2.69(2.45) |
| Gynaecomastia | 69 | 49.09(12.03,200.26) | 49.09(91.57) | 5.58(-0.18) | 47.74(11.70) |
| Bradycardia | 432 | 6.00(5.45,6.59) | 5.96(1780.67) | 2.57(2.42) | 5.95(5.41) |
| Confusional state | 363 | 2.28(2.05,2.52) | 2.27(257.85) | 1.18(1.02) | 2.27(2.04) |
| Blood creatinine increased | 498 | 8.39(7.68,9.17) | 8.34(3202.88) | 3.05(2.90) | 8.30(7.60) |
| Renal failure | 485 | 4.11(3.76,4.50) | 4.09(1131.95) | 2.03(1.89) | 4.08(3.73) |
| Hypokalaemia* | 322 | 4.99(4.47,5.57) | 4.97(1019.91) | 2.31(2.13) | 4.96(4.45) |
| Renal impairment | 681 | 9.10(8.44,9.82) | 9.02(4837.18) | 3.17(3.04) | 8.98(8.33) |
| Blood potassium increased | 604 | 53.13(48.98,57.63) | 52.68(29702.5) | 5.68(5.44) | 51.12(47.13) |
| General physical health deterioration* | 135 | 2.18(1.84,2.58) | 2.18(85.70) | 1.12(0.86) | 2.17(1.84) |
| Syncope | 271 | 1.63(1.44,1.83) | 1.62(64.83) | 0.70(0.52) | 1.62(1.44) |
| Cardiac failure* | 287 | 5.28(4.71,5.94) | 5.27(989.82) | 2.39(2.20) | 5.25(4.68) |
| Orthostatic hypotension | 198 | 8.78(7.64,10.10) | 8.76(1354.43) | 3.12(2.86) | 8.72(7.58) |
| Breast pain | 505 | 23.43(21.45,25.59) | 23.27(10619.0) | 4.52(4.33) | 22.97(21.03) |
| Hepatic encephalopathy | 142 | 26.38(22.35,31.15) | 26.33(3407.93) | 4.70(4.22) | 25.94(21.98) |
| Metabolic acidosis | 137 | 8.55(7.23,10.11) | 8.53(906.80) | 3.09(2.76) | 8.50(7.18) |
| Product substitution issue* | 122 | 2.44(2.04,2.91) | 2.43(103.08) | 1.28(1.01) | 2.43(2.04) |
| Blood pressure decreased | 220 | 3.25(2.85,3.72) | 3.25(341.80) | 1.70(1.49) | 3.24(2.84) |
| Pemphigoid | 59 | 8.61(6.66,11.12) | 8.60(394.35) | 3.10(2.55) | 8.56(6.63) |
| Product odour abnormal* | 74 | 10.93(8.70,13.74) | 10.92(662.65) | 3.44(2.93) | 10.86(8.64) |
| Arrhythmia | 106 | 1.62(1.34,1.96) | 1.62(24.98) | 0.69(0.41) | 1.62(1.34) |
| Electrolyte imbalance | 208 | 22.46(19.58,25.76) | 22.39(4196.30) | 4.47(4.13) | 22.11(19.28) |
| Lactic acidosis* | 63 | 3.41(2.66,4.36) | 3.40(106.71) | 1.76(1.35) | 3.40(2.65) |
| Breast tenderness | 107 | 14.62(12.09,17.69) | 14.60(1344.37) | 3.86(3.41) | 14.49(11.97) |
| **Eplerenone** | | | | | |
| **PT** | **N** | **ROR (95% Cl)** | **PRR (χ^2^)** | **IC (IC025)** | **EBGM (EBGM05)** |
| Acute kidney injury | 210 | 11.02(9.61,12.64) | 10.75(1859.51) | 3.42(3.16) | 10.74(9.36) |
| Hyperkalaemia | 292 | 75.89(67.49,85.33) | 73.01(20652.3) | 6.18(5.70) | 72.67(64.63) |
| Hypotension | 145 | 5.39(4.57,6.35) | 5.30(507.90) | 2.41(2.12) | 5.30(4.50) |
| Cardiac failure* | 93 | 15.80(12.88,19.39) | 15.62(1272.56) | 3.96(3.46) | 15.61(12.72) |
| Drug interaction | 44 | 4.04(3.00,5.43) | 4.02(100.06) | 2.01(1.48) | 4.02(2.99) |
| Hyponatraemia | 64 | 9.07(7.09,11.60) | 9.00(455.53) | 3.17(2.64) | 9.00(7.04) |
| Renal impairment* | 100 | 12.25(10.05,14.92) | 12.10(1018.33) | 3.60(3.16) | 12.09(9.92) |
| Renal failure* | 45 | 3.49(2.60,4.68) | 3.47(79.41) | 1.80(1.29) | 3.47(2.59) |
| Dehydration | 46 | 4.31(3.22,5.75) | 4.29(116.04) | 2.10(1.58) | 4.29(3.21) |
| Blood potassium increased | 60 | 47.06(36.49,60.69) | 46.69(2675.22) | 5.54(4.36) | 46.56(36.10) |
| Oedema peripheral | 61 | 3.30(2.56,4.24) | 3.28(96.84) | 1.71(1.29) | 3.28(2.55) |
| Blood creatinine increased | 48 | 7.38(5.56,9.80) | 7.34(262.90) | 2.87(2.29) | 7.34(5.52) |
| Hypokalaemia* | 39 | 5.53(4.04,7.58) | 5.51(144.06) | 2.46(1.85) | 5.51(4.02) |
| General physical health deterioration | 26 | 3.85(2.62,5.66) | 3.84(54.62) | 1.94(1.24) | 3.84(2.61) |
| Atrial fibrillation* | 35 | 4.34(3.12,6.06) | 4.33(89.66) | 2.11(1.50) | 4.33(3.10) |
| Bradycardia* | 28 | 3.54(2.45,5.14) | 3.53(50.93) | 1.82(1.16) | 3.53(2.44) |
| Blood potassium decreased* | 25 | 10.73(7.24,15.89) | 10.69(219.62) | 3.42(2.39) | 10.69(7.22) |
| Syncope | 5 | 1.15(0.48,2.77) | 1.15(0.10) | 0.21(-1.01) | 1.15(0.48) |
| Glomerular filtration rate decreased* | 24 | 25.33(16.96,37.82) | 25.25(558.08) | 4.66(3.10) | 25.21(16.88) |
| Gynaecomastia | 76 | 21.27(16.97,26.67) | 21.07(1451.45) | 4.40(3.73) | 21.04(16.78) |
| Arrhythmia | 36 | 5.05(3.64,7.01) | 5.04(116.50) | 2.33(1.71) | 5.03(3.63) |
| Blood pressure decreased | 35 | 4.75(3.41,6.62) | 4.73(103.05) | 2.24(1.62) | 4.73(3.39) |
| Cardiac failure chronic* | 27 | 104.55(71.56,152.76) | 104.19(2740.98) | 6.69(3.92) | 103.50(70.84) |
| Contraindicated product administered | 26 | 18.47(12.56,27.15) | 18.41(427.56) | 4.20(2.93) | 18.39(12.51) |
| Orthostatic hypotension | 27 | 10.94(7.50,15.97) | 10.91(242.90) | 3.45(2.46) | 10.90(7.47) |
| Vertigo | 20 | 1.51(0.97,2.34) | 1.50(3.39) | 0.59(-0.08) | 1.50(0.97) |
| Alanine aminotransferase increased | 11 | 1.30(0.72,2.36) | 1.30(0.78) | 0.38(-0.49) | 1.30(0.72) |
| Pulmonary oedema* | 22 | 5.98(3.94,9.09) | 5.97(90.98) | 2.58(1.69) | 5.97(3.93) |
| Product prescribing error* | 15 | 3.89(2.35,6.46) | 3.89(32.18) | 1.96(1.00) | 3.89(2.34) |
| Circulatory collapse | 7 | 2.54(1.21,5.34) | 2.54(6.55) | 1.35(0.07) | 2.54(1.21) |
| **Finerenone** | | | | | |
| Glomerular filtration rate decreased | 196 | 694.54(599.72,804.36) | 639.64(123337) | 9.30(7.02) | 631.18(545.01) |
| Hyperkalaemia | 290 | 251.66(222.59,284.52) | 222.30(63625.1) | 7.79(6.80) | 221.27(195.71) |
| Blood creatinine increased | 105 | 51.38(42.26,62.48) | 49.25(4962.23) | 5.62(4.79) | 49.20(40.46) |
| Blood potassium increased | 158 | 404.59(344.17,475.63) | 378.84(59080.9) | 8.55(6.57) | 375.86(319.72) |
| Death* | 62 | 3.16(2.46,4.07) | 3.11(89.25) | 1.63(1.22) | 3.11(2.41) |
| Renal impairment* | 29 | 10.87(7.53,15.67) | 10.75(256.68) | 3.43(2.49) | 10.75(7.45) |
| Dizziness* | 60 | 1.59(1.23,2.06) | 1.58(12.99) | 0.66(0.27) | 1.58(1.22) |
| Acute kidney injury* | 37 | 5.87(4.25,8.13) | 5.80(147.38) | 2.54(1.89) | 5.80(4.19) |
| Hypotension | 48 | 5.47(4.11,7.28) | 5.38(171.80) | 2.43(1.89) | 5.38(4.04) |
| Urine albumin/creatinine ratio increased* | 24 | 3094.68(2044.26,4684.84) | 3064.69(69061.2) | 11.49(4.03) | 2879.48(1902.11) |
| Hyponatraemia | 33 | 14.41(10.22,20.32) | 14.23(406.19) | 3.83(2.86) | 14.23(10.09) |
| Renal failure* | 13 | 3.09(1.79,5.33) | 3.08(18.25) | 1.62(0.65) | 3.08(1.78) |
| Product prescribing issue | 8 | 19.03(9.50,38.10) | 18.97(136.15) | 4.25(1.70) | 18.96(9.47) |
| Blood pressure decreased | 14 | 5.83(3.45,9.86) | 5.80(55.67) | 2.54(1.39) | 5.80(3.43) |
| Blood creatine increased* | 9 | 72.23(37.52,139.06) | 71.97(628.97) | 6.17(2.24) | 71.87(37.33) |
| Urine albumin/creatinine ratio decreased* | 7 | 14537.3(6232.23,33909.7) | 14496.2(77785.6) | 13.44(1.85) | 11114.0(4764.63) |
| Albuminuria* | 9 | 114.27(59.34,220.05) | 113.86(1004.43) | 6.83(2.30) | 113.59(58.98) |
| Proteinuria* | 9 | 17.02(8.84,32.75) | 16.96(135.13) | 4.08(1.79) | 16.95(8.81) |
| Blood potassium decreased* | 5 | 6.57(2.73,15.79) | 6.55(23.54) | 2.71(0.59) | 6.55(2.73) |
| Glomerular filtration rate increased* | 8 | 411.05(204.72,825.32) | 409.72(3234.02) | 8.67(2.17) | 406.24(202.33) |
| Albumin urine present* | 4 | 433.70(161.92,1161.65) | 433.00(1708.50) | 8.75(1.01) | 429.11(160.21) |
| Blood potassium abnormal | 7 | 126.07(59.98,265.00) | 125.72(863.84) | 6.97(1.90) | 125.39(59.66) |
| Blood pressure systolic increased* | 5 | 16.59(6.90,39.90) | 16.56(73.07) | 4.05(1.02) | 16.55(6.88) |
| Blood urea increased* | 7 | 11.15(5.31,23.42) | 11.13(64.51) | 3.48(1.27) | 11.12(5.30) |
| Glomerular filtration rate abnormal | 4 | 108.42(40.62,289.44) | 108.25(424.08) | 6.75(0.98) | 108.01(40.46) |
| Renal pain* | 4 | 8.59(3.22,22.90) | 8.58(26.77) | 3.10(0.48) | 8.58(3.22) |
| Thirst* | 3 | 3.56(1.15,11.06) | 3.56(5.53) | 1.83(-0.33) | 3.56(1.15) |
| Protein urine present* | 3 | 24.83(8.00,77.07) | 24.80(68.50) | 4.63(0.39) | 24.79(7.99) |
| Labelled drug-drug interaction medication error | 5 | 20.37(8.47,48.99) | 20.33(91.87) | 4.34(1.09) | 20.32(8.45) |
| Acidosis* | 3 | 11.42(3.68,35.44) | 11.41(28.49) | 3.51(0.22) | 11.41(3.68) |

Note: *N*, number of AEs reported; *ROR*, reporting odds ratio; *PRR*, proportional reporting ratio; *IC*, information components; *EBGM*, empirical bayesian geometric mean; *CI*, confidence interval.; *χ^2^*, Chi-square test; *IC025*,the lower limit of 95% CI for IC; *EBGM05*, the lower limit of 95%CI for EBGM value; *, The instruction does not mention.

TableS9. Validation of the Top 30 PTs With the Highest Signal Intensity in FAERS Using the WHO VigiAccess Database

| **Spironolactone** | | | | | |
| --- | --- | --- | --- | --- | --- |
| **PT** | **N** | **ROR (95% Cl)** | **PRR (χ^2^)** | **IC (IC025)** | **EBGM (EBGM05)** |
| Endometriosis male* | 2 | 1129.13(188.66,6757.74) | 1129.10(1352.52) | 9.40(-0.46) | 677.86(113.26) |
| 5-alpha-reductase deficiency* | 2 | 483.91(100.52,2329.52) | 483.90(749.62) | 8.56(-0.32) | 376.59(78.23) |
| Secondary sexual characteristics absence* | 1 | 99.63(13.26,748.65) | 99.63(92.21) | 6.56(-1.16) | 94.15(12.53) |
| Blood aldosterone abnormal | 10 | 63.68(33.87,119.73) | 63.67(594.52) | 5.94(2.35) | 61.40(32.66) |
| Bulbospinal muscular atrophy congenital* | 6 | 923.89(341.66,2498.27) | 923.81(3578.78) | 9.22(1.51) | 598.11(221.19) |
| Double hit lymphoma* | 2 | 56.46(13.80,230.98) | 56.45(105.43) | 5.77(-0.17) | 54.67(13.36) |
| Hypocalvaria* | 73 | 5.35(4.25,6.73) | 5.35(257.23) | 2.42(2.00) | 5.33(4.24) |
| Female sexual arousal disorder | 5 | 76.99(31.42,188.66) | 76.98(358.68) | 6.20(1.28) | 73.68(30.07) |
| Genitalia external ambiguous* | 12 | 93.67(52.38,167.52) | 93.66(1042.38) | 6.47(2.69) | 88.80(49.66) |
| Gender dysphoria* | 4 | 55.53(20.51,150.34) | 55.53(207.39) | 5.75(0.90) | 53.80(19.87) |
| Asthenospermia | 1 | 22.89(3.18,164.65) | 22.89(20.65) | 4.50(-1.13) | 22.60(3.14) |
| Blood aldosterone increased | 10 | 63.68(33.87,119.73) | 63.67(594.52) | 5.94(2.35) | 61.40(32.66) |
| Spur cell anaemia* | 1 | 36.82(5.08,266.99) | 36.82(34.10) | 5.17(-1.12) | 36.06(4.97) |
| Hyperkalaemia | 261 | 204.96(199.43,210.65) | 186.79(1032636) | 7.40(7.32) | 168.34(163.79) |
| Electrocardiogram T wave peaked* | 26 | 114.12(76.71,169.77) | 114.08(2730.40) | 6.74(3.87) | 106.94(71.89) |
| Electrocardiogram T wave amplitude increased* | 4 | 64.52(23.77,175.13) | 64.52(240.96) | 5.96(0.91) | 62.19(22.91) |
| Oligospermia | 1 | 2.70(0.38,19.21) | 2.70(1.07) | 1.43(-1.50) | 2.70(0.38) |
| Urticaria cholinergic | 1 | 10.72(1.50,76.57) | 10.72(8.76) | 3.41(-1.18) | 10.66(1.49) |
| Acute cutaneous lupus erythematosus | 2 | 24.73(6.12,99.87) | 24.72(44.88) | 4.61(-0.21) | 24.38(6.04) |
| Therapeutic drug monitoring analysis not performed* | 1 | 25.66(3.56,184.90) | 25.66(23.35) | 4.66(-1.13) | 25.29(3.51) |
| Spermatogenesis abnormal | 1 | 3.63(0.51,25.80) | 3.63(1.90) | 1.86(-1.40) | 3.62(0.51) |
| Hypoosmolar state | 2 | 20.16(5.00,81.29) | 20.16(36.00) | 4.32(-0.23) | 19.94(4.95) |
| Angiotensin converting enzyme inhibitor foetopathy* | 1 | 1.80(0.25,12.80) | 1.80(0.36) | 0.85(-1.68) | 1.80(0.25) |
| Renin increased | 9 | 64.60(33.20,125.70) | 64.59(542.74) | 5.96(2.20) | 62.25(31.99) |
| Neonatal hyponatraemia | 1 | 12.36(1.73,88.40) | 12.36(10.37) | 3.62(-1.17) | 12.28(1.72) |
| Blood pressure orthostatic | 2 | 16.94(4.21,68.19) | 16.94(29.69) | 4.07(-0.26) | 16.78(4.17) |
| Gastrointestinal malformation* | 1 | 3.70(0.52,26.31) | 3.70(1.96) | 1.88(-1.39) | 3.69(0.52) |
| Nipple pain | 149 | 70.72(60.02,83.32) | 70.57(9810.37) | 6.08(5.31) | 67.79(57.53) |
| Sinoatrial block* | 13 | 30.50(17.62,52.79) | 30.49(364.30) | 4.91(2.51) | 29.97(17.32) |
| Nipple swelling | 14 | 52.35(30.76,89.11) | 52.34(683.92) | 5.67(2.80) | 50.80(29.85) |
| **Eplerenone** | | | | | |
| **PT** | **N** | **ROR (95% Cl)** | **PRR (χ^2^)** | **IC (IC025)** | **EBGM (EBGM05)** |
| Venous pressure jugular decreased* | 1 | 597.59(81.08,4404.41) | 597.51(573.45) | 9.17(-1.11) | 575.42(78.07) |
| Labelled drug-disease interaction issue* | 1 | 194.22(27.02,1395.92) | 194.19(189.82) | 7.58(-1.07) | 191.81(26.69) |
| Hyperaldosteronism | 4 | 185.59(69.23,497.52) | 185.49(725.34) | 7.52(0.99) | 183.32(68.38) |
| Giardiasis* | 1 | 45.17(6.34,321.61) | 45.16(43.06) | 5.49(-1.08) | 45.03(6.32) |
| Hyperadrenocorticism* | 1 | 24.82(3.49,176.50) | 24.82(22.82) | 4.63(-1.10) | 24.78(3.48) |
| Therapeutic drug monitoring analysis not performed* | 1 | 65.28(9.16,465.43) | 65.27(63.02) | 6.02(-1.07) | 65.01(9.12) |
| Globulins increased* | 1 | 20.13(2.83,143.08) | 20.12(18.15) | 4.33(-1.11) | 20.10(2.83) |
| N-terminal prohormone brain natriuretic peptide increased* | 14 | 121.05(71.51,204.91) | 120.83(1650.91) | 6.91(3.00) | 119.90(70.83) |
| Venous pressure jugular increased* | 1 | 1553.73(198.86,12139.3) | 1553.52(1410.47) | 10.46(-1.21) | 1412.38(180.77) |
| BRASH syndrome* |  |  |  |  |  |
| Cardiac failure chronic* | 27 | 104.55(71.56,152.76) | 104.19(2740.98) | 6.69(3.92) | 103.50(70.84) |
| Jugular vein distension* | 2 | 56.40(14.07,226.14) | 56.39(108.42) | 5.81(-0.14) | 56.19(14.01) |
| Hyperkalaemia | 292 | 75.89(67.49,85.33) | 73.01(20652.3) | 6.18(5.70) | 72.67(64.63) |
| Scrotal oedema* | 1 | 10.98(1.55,78.02) | 10.98(9.06) | 3.46(-1.17) | 10.97(1.54) |
| Cardiac amyloidosis* | 3 | 48.87(15.73,151.84) | 48.85(140.18) | 5.61(0.47) | 48.70(15.68) |
| Waist circumference increased* | 6 | 72.59(32.54,161.94) | 72.54(421.35) | 6.17(1.60) | 72.21(32.37) |
| Product monitoring error* | 2 | 22.75(5.68,91.08) | 22.75(41.52) | 4.51(-0.20) | 22.71(5.67) |
| Labelled drug-drug interaction issue | 3 | 22.82(7.35,70.83) | 22.81(62.48) | 4.51(0.38) | 22.78(7.34) |
| Blood potassium increased | 60 | 47.06(36.49,60.69) | 46.69(2675.22) | 5.54(4.36) | 46.56(36.10) |
| Creatinine renal clearance decreased | 8 | 19.31(9.65,38.64) | 19.29(138.57) | 4.27(1.71) | 19.27(9.63) |
| Arrhythmia supraventricular | 2 | 8.94(2.23,35.76) | 8.94(14.09) | 3.16(-0.37) | 8.93(2.23) |
| Glomerular filtration rate decreased | 24 | 25.33(16.96,37.82) | 25.25(558.08) | 4.66(3.10) | 25.21(16.88) |
| Pulmonary arterial pressure increased* | 4 | 28.59(10.72,76.25) | 28.57(106.23) | 4.83(0.84) | 28.52(10.69) |
| Dilatation atrial* | 1 | 11.32(1.59,80.40) | 11.31(9.40) | 3.50(-1.16) | 11.31(1.59) |
| Sleep deficit | 3 | 18.31(5.90,56.81) | 18.30(49.00) | 4.19(0.34) | 18.28(5.89) |
| Marasmus* | 4 | 100.60(37.63,268.97) | 100.55(391.71) | 6.64(0.97) | 99.91(37.37) |
| Blood pressure inadequately controlled | 15 | 29.74(17.91,49.38) | 29.69(415.02) | 4.89(2.69) | 29.63(17.85) |
| Brain natriuretic peptide increased* | 8 | 31.06(15.51,62.17) | 31.02(231.99) | 4.95(1.87) | 30.96(15.47) |
| Renal tubular acidosis* | 2 | 16.14(4.03,64.61) | 16.14(28.38) | 4.01(-0.25) | 16.12(4.03) |
| Ventricular arrhythmia | 1 | 46.94(6.59,334.27) | 46.93(44.82) | 5.55(-1.08) | 46.80(6.57) |
| **Finerenone** | | | | | |
| Urine albumin/creatinine ratio decreased* | 7 | 14537.3(6232.23,33909.7) | 14496.2(77785.6) | 13.44(1.85) | 11114.0(4764.63) |
| Urine albumin/creatinine ratio increased* | 24 | 3094.68(2044.26,4684.84) | 3064.69(69061.2) | 11.49(4.03) | 2879.48(1902.11) |
| Glomerular filtration rate decreased | 196 | 694.54(599.72,804.36) | 639.64(123337) | 9.30(7.02) | 631.18(545.01) |
| Albumin urine present* | 4 | 433.70(161.92,1161.65) | 433.00(1708.50) | 8.75(1.01) | 429.11(160.21) |
| Albuminuria* | 9 | 114.27(59.34,220.05) | 113.86(1004.43) | 6.83(2.30) | 113.59(58.98) |
| Glomerular filtration rate increased* | 8 | 411.05(204.72,825.32) | 409.72(3234.02) | 8.67(2.17) | 406.24(202.33) |
| Blood potassium increased | 158 | 404.59(344.17,475.63) | 378.84(59080.9) | 8.55(6.57) | 375.86(319.72) |
| Hyperkalaemia | 290 | 251.66(222.59,284.52) | 222.30(63625.1) | 7.79(6.80) | 221.27(195.71) |
| Glomerular filtration rate abnormal | 4 | 108.42(40.62,289.44) | 108.25(424.08) | 6.75(0.98) | 108.01(40.46) |
| Blood creatine increased* | 9 | 72.23(37.52,139.06) | 71.97(628.97) | 6.17(2.24) | 71.87(37.33) |
| Blood creatinine increased | 105 | 51.38(42.26,62.48) | 49.25(4962.23) | 5.62(4.79) | 49.20(40.46) |
| Blood potassium abnormal | 7 | 126.07(59.98,265.00) | 125.72(863.84) | 6.97(1.90) | 125.39(59.66) |
| Renal impairment | 29 | 10.87(7.53,15.67) | 10.75(256.68) | 3.43(2.49) | 10.75(7.45) |
| Product prescribing issue* | 8 | 19.03(9.50,38.10) | 18.97(136.15) | 4.25(1.70) | 18.96(9.47) |
| Protein urine present* | 3 | 24.83(8.00,77.07) | 24.80(68.50) | 4.63(0.39) | 24.79(7.99) |
| Renal function test abnormal | 3 | 23.09(7.44,71.67) | 23.07(63.30) | 4.53(0.38) | 23.05(7.43) |
| Blood pressure systolic decreased | 3 | 24.01(7.74,74.53) | 23.99(66.06) | 4.58(0.39) | 23.98(7.72) |
| Blood pressure diastolic increased* | 2 | 26.70(6.67,106.88) | 26.68(49.42) | 4.74(-0.19) | 26.67(6.66) |
| Hyponatraemia | 33 | 14.41(10.22,20.32) | 14.23(406.19) | 3.83(2.86) | 14.23(10.09) |
| Proteinuria* | 9 | 17.02(8.84,32.75) | 16.96(135.13) | 4.08(1.79) | 16.95(8.81) |
| Labelled drug-drug interaction medication error* | 5 | 20.37(8.47,48.99) | 20.33(91.87) | 4.34(1.09) | 20.32(8.45) |
| Feeling drunk* | 2 | 7.63(1.91,30.53) | 7.63(11.51) | 2.93(-0.42) | 7.62(1.91) |
| Acidosis* | 3 | 11.42(3.68,35.44) | 11.41(28.49) | 3.51(0.22) | 11.41(3.68) |
| Renal pain* | 4 | 8.59(3.22,22.90) | 8.58(26.77) | 3.10(0.48) | 8.58(3.22) |
| Flank pain* | 3 | 9.66(3.11,29.98) | 9.65(23.27) | 3.27(0.17) | 9.65(3.11) |
| Blood urea increased* | 7 | 11.15(5.31,23.42) | 11.13(64.51) | 3.48(1.27) | 11.12(5.30) |
| Blood pressure systolic increased* | 5 | 16.59(6.90,39.90) | 16.56(73.07) | 4.05(1.02) | 16.55(6.88) |
| Acute kidney injury | 37 | 5.87(4.25,8.13) | 5.80(147.38) | 2.54(1.89) | 5.80(4.19) |
| Blood potassium decreased* | 5 | 6.57(2.73,15.79) | 6.55(23.54) | 2.71(0.59) | 6.55(2.73) |
| Thirst* | 3 | 3.56(1.15,11.06) | 3.56(5.53) | 1.83(-0.33) | 3.56(1.15) |

Note: *N*, number of AEs reported; *ROR*, reporting odds ratio; *PRR*, proportional reporting ratio; *IC*, information components; *EBGM*, empirical bayesian geometric mean; *CI*, confidence interval.; *χ^2^*, Chi-square test; *IC025*,the lower limit of 95% CI for IC; *EBGM05*, the lower limit of 95%CI for EBGM value; *, The instruction does not mention.

Table S10. Comparison of Sex-related AE Signals Among Three MRAs in the FAERS database

| PT | n | ROR(95% CI) | PRR(**χ^2^**) | IC(IC025) | EBGM(EBGM05) |
| --- | --- | --- | --- | --- | --- |
| **Spironolactone** |  |  |  |  |  |
| Gynaecomastia | 295 | 19.90(17.74,22.33) | 19.70(5188.53) | 4.29(4.03) | 19.52(17.39) |
| Breast pain | 110 | 22.60(18.72,27.29) | 22.52(2237.11) | 4.48(3.95) | 22.28(18.45) |
| Breast tenderness | 62 | 17.49(13.62,22.46) | 17.45(953.27) | 4.11(3.42) | 17.31(13.48) |
| Nipple pain | 44 | 49.37(36.60,66.60) | 49.29(2031.71) | 5.59(4.12) | 48.13(35.68) |
| Erectile dysfunction | 41 | 3.60(2.65,4.90) | 3.60(76.85) | 1.85(1.31) | 3.59(2.65) |
| Breast mass | 39 | 12.89(9.41,17.66) | 12.87(424.41) | 3.68(2.85) | 12.80(9.34) |
| Breast enlargement | 38 | 22.63(16.43,31.16) | 22.60(775.69) | 4.48(3.39) | 22.36(16.23) |
| Breast swelling | 30 | 24.18(16.87,34.67) | 24.16(658.09) | 4.58(3.26) | 23.88(16.66) |
| Hair growth abnormal | 18 | 5.74(3.61,9.12) | 5.74(70.22) | 2.52(1.53) | 5.72(3.60) |
| Loss of libido | 17 | 4.30(2.67,6.93) | 4.30(42.97) | 2.10(1.18) | 4.29(2.67) |
| Genitalia external ambiguous | 14 | 182.77(105.72,315.96) | 182.68(2317.48) | 7.39(3.01) | 167.45(96.86) |
| Vulvovaginal pain | 11 | 4.96(2.74,8.96) | 4.95(34.64) | 2.31(1.06) | 4.95(2.74) |
| Hypertrichosis | 10 | 11.44(6.14,21.30) | 11.43(94.68) | 3.51(1.68) | 11.37(6.11) |
| Dyspareunia | 8 | 4.13(2.06,8.26) | 4.12(18.90) | 2.04(0.65) | 4.12(2.06) |
| Female sexual arousal disorder | 8 | 210.21(101.44,435.59) | 210.15(1506.61) | 7.57(2.10) | 190.23(91.80) |
| Blood testosterone increased | 7 | 7.21(3.43,15.14) | 7.20(37.26) | 2.84(1.00) | 7.18(3.42) |
| Nipple disorder | 7 | 23.94(11.36,50.43) | 23.93(151.98) | 4.56(1.60) | 23.66(11.23) |
| Breast discomfort | 7 | 14.65(6.97,30.82) | 14.65(88.37) | 3.86(1.41) | 14.55(6.92) |
| Endometriosis male | 7 | 13978.2(1719.68,113620) | 13974.7(12226.1) | 10.77(1.59) | 1747.71(215.01) |
| Blood oestrogen increased | 6 | 23.40(10.46,52.33) | 23.40(127.14) | 4.53(1.38) | 23.14(10.34) |
| Breast discharge | 6 | 7.36(3.30,16.42) | 7.36(32.87) | 2.88(0.85) | 7.34(3.29) |
| Penis disorder | 6 | 5.13(2.30,11.42) | 5.13(19.87) | 2.35(0.59) | 5.11(2.30) |
| Testicular swelling | 5 | 8.64(3.59,20.81) | 8.64(33.65) | 3.11(0.74) | 8.61(3.58) |
| Nipple swelling | 5 | 44.37(18.29,107.65) | 44.36(207.33) | 5.44(1.23) | 43.42(17.90) |
| Hormone receptor positive breast cancer | 5 | 31.20(12.90,75.48) | 31.19(143.88) | 4.94(1.18) | 30.73(12.70) |
| Hirsutism | 4 | 6.48(2.43,17.30) | 6.48(18.48) | 2.69(0.33) | 6.46(2.42) |
| Oligospermia | 4 | 87.77(32.24,238.90) | 87.75(328.61) | 6.39(0.93) | 84.10(30.90) |
| Scrotal oedema | 4 | 12.19(4.56,32.59) | 12.19(40.85) | 3.60(0.61) | 12.12(4.54) |
| Asthenospermia | 4 | 135.37(49.17,372.69) | 135.35(499.55) | 6.99(0.94) | 126.82(46.06) |
| Prolactin-producing pituitary tumour | 3 | 19.57(6.28,61.03) | 19.57(52.36) | 4.28(0.34) | 19.39(6.22) |
| Spermatogenesis abnormal | 3 | 76.79(24.24,243.31) | 76.78(216.08) | 6.21(0.46) | 73.98(23.35) |
| Secondary sexual characteristics absence | 3 | 748.72(198.62,2822.42) | 748.64(1629.04) | 9.09(0.33) | 544.74(144.51) |
| Disturbance in sexual arousal | 3 | 7.19(2.31,22.34) | 7.19(15.93) | 2.84(0.05) | 7.17(2.31) |
| Breast cancer male | 3 | 12.58(4.04,39.16) | 12.58(31.79) | 3.64(0.24) | 12.51(4.02) |
| **Eplerenone** |  |  |  |  |  |
| Gynaecomastia | 26 | 8.11(5.52,11.92) | 8.08(161.22) | 3.01(2.12) | 8.07(5.49) |
| Erectile dysfunction | 13 | 5.36(3.11,9.24) | 5.35(45.98) | 2.42(1.26) | 5.35(3.10) |
| Breast pain | 11 | 10.48(5.80,18.95) | 10.47(94.08) | 3.39(1.71) | 10.45(5.78) |
| Breast tenderness | 6 | 7.87(3.53,17.54) | 7.87(35.93) | 2.97(0.90) | 7.86(3.53) |
| Breast swelling | 5 | 18.73(7.79,45.05) | 18.71(83.68) | 4.22(1.06) | 18.68(7.76) |
| Breast enlargement | 3 | 8.29(2.67,25.73) | 8.29(19.21) | 3.05(0.11) | 8.28(2.67) |
| Nipple pain | 3 | 15.43(4.97,47.90) | 15.42(40.40) | 3.94(0.30) | 15.40(4.96) |
|  |  |  |  |  |  |

Note: *N*, number of AEs reported; *ROR*, reporting odds ratio; *PRR*, proportional reporting ratio; *IC*, information components; *EBGM*, empirical bayesian geometric mean; *CI*, confidence interval.; *χ^2^*, Chi-square test; *IC025*,the lower limit of 95% CI for IC; *EBGM05*, the lower limit of 95%CI for EBGM value; *, The instruction does not mention.

Table S11. Comparison of congenital anomaly-related AE signals among the three MRAs in the FAERS Database

| PT | n | ROR(95% CI) | PRR(**χ^2^**) | IC(IC025) | EBGM(EBGM05) |  |
| --- | --- | --- | --- | --- | --- | --- |
| **Spironolactone** |  |  |  |  |  |  |
| Genitalia external ambiguous | 14 | 182.77(105.72,315.96) | 182.68(2317.48) | 7.39(3.01) | 167.45(96.86) |  |
| Dysmorphism | 13 | 10.45(6.06,18.03) | 10.45(110.50) | 3.38(1.86) | 10.40(6.03) |  |
| Hypocalvaria | 10 | 212.46(110.69,407.81) | 212.38(1901.57) | 7.59(2.47) | 192.06(100.06) |  |
| Hypospadias | 9 | 9.61(4.99,18.50) | 9.61(69.08) | 3.26(1.45) | 9.57(4.97) |  |
| Bulbospinal muscular atrophy congenital | 6 | 374.40(156.54,895.49) | 374.32(1881.22) | 8.30(1.59) | 315.38(131.86) |  |
| Congenital musculoskeletal disorder of limbs | 6 | 14.21(6.37,31.73) | 14.21(73.16) | 3.82(1.20) | 14.12(6.32) |  |
| 5-alpha-reductase deficiency | 5 | 1663.95(507.78,5452.60) | 1663.65(4531.78) | 9.83(1.13) | 907.90(277.06) |  |
| Gastrointestinal malformation | 5 | 52.00(21.40,126.36) | 51.99(243.70) | 5.66(1.25) | 50.69(20.86) |  |
| Hypertrophic cardiomyopathy | 4 | 5.83(2.19,15.57) | 5.83(15.97) | 2.54(0.27) | 5.82(2.18) |  |
| Congenital musculoskeletal disorder | 4 | 16.13(6.03,43.16) | 16.13(56.32) | 4.00(0.70) | 16.01(5.99) |  |
| Bicuspid aortic valve | 3 | 11.20(3.60,34.83) | 11.19(27.70) | 3.48(0.21) | 11.14(3.58) |  |
| Familial periodic paralysis | 3 | 38.64(12.33,121.14) | 38.64(107.91) | 5.25(0.43) | 37.92(12.10) |  |
| Potter's syndrome | 3 | 41.03(13.08,128.69) | 41.02(114.78) | 5.33(0.43) | 40.22(12.82) |  |
| Angiotensin converting enzyme inhibitor foetopathy | 3 | 68.07(21.54,215.14) | 68.06(191.69) | 6.04(0.46) | 65.85(20.83) |  |
| Congenital renal disorder | 3 | 23.67(7.58,73.90) | 23.67(64.38) | 4.55(0.37) | 23.41(7.50) |  |
| **Eplerenone** |  |  |  |  |  |  |
| Ventricular septal defect | 4 | 6.91(2.59,18.42) | 6.91(20.19) | 2.79(0.37) | 6.90(2.59) |  |

Note: *N*, number of AEs reported; *ROR*, reporting odds ratio; *PRR*, proportional reporting ratio; *IC*, information components; *EBGM*, empirical bayesian geometric mean; *CI*, confidence interval.; *χ^2^*, Chi-square test; *IC025*,the lower limit of 95% CI for IC; *EBGM05*, the lower limit of 95%CI for EBGM value; *, The instruction does not mention.

TableS12. Gender-Stratified Subgroup Analysis of AEs Associated With MRAs in FAERS

| **Female** | | | | | | **Male** | | | | | |
| --- | --- | --- | --- | --- | --- | --- | --- | --- | --- | --- | --- |
| **PT** | **N** | **ROR (95% Cl)** | **PRR (χ^2^)** | **IC (IC025)** | **EBGM (EBGM05)** | **PT** | **N** | **ROR (95% Cl)** | **PRR (χ^2^)** | **IC (IC025)** | **EBGM (EBGM05)** |
| **Spironolactone** | | | | | | | | | | | |
| Hyperkalaemia | 504 | 98.97(90.39,108.37) | 95.58(45173.4) | 6.52(6.14) | 91.54(83.60) | Hyperkalaemia | 744 | 83.61(77.49,90.20) | 78.24(54192.9) | 6.22(5.98) | 74.72(69.25) |
| Acute kidney injury | 496 | 15.78(14.42,17.26) | 15.28(6585.98) | 3.92(3.75) | 15.18(13.87) | Acute kidney injury | 530 | 11.10(10.17,12.12) | 10.64(4617.03) | 3.40(3.25) | 10.57(9.69) |
| Drug interaction | 318 | 10.43(9.33,11.66) | 10.22(2638.46) | 3.35(3.14) | 10.18(9.10) | Drug interaction | 319 | 8.95(8.01,10.01) | 8.73(2179.25) | 3.12(2.92) | 8.69(7.77) |
| Hyponatraemia | 305 | 24.73(22.06,27.71) | 24.23(6722.39) | 4.58(4.31) | 23.97(21.39) | Gynaecomastia | 270 | 15.47(13.70,17.47) | 15.13(3535.89) | 3.91(3.66) | 15.00(13.29) |
| Dehydration | 191 | 6.17(5.35,7.11) | 6.10(813.74) | 2.61(2.36) | 6.08(5.27) | Hypotension | 229 | 5.07(4.45,5.78) | 4.99(731.96) | 2.32(2.10) | 4.98(4.37) |
| Hypotension | 180 | 4.34(3.74,5.03) | 4.30(455.74) | 2.10(1.86) | 4.29(3.70) | Dehydration | 190 | 6.87(5.95,7.93) | 6.77(933.70) | 2.76(2.50) | 6.75(5.85) |
| Drug hypersensitivity | 141 | 2.56(2.17,3.03) | 2.55(132.98) | 1.35(1.09) | 2.55(2.16) | Hyponatraemia | 151 | 13.76(11.71,16.17) | 13.59(1749.11) | 3.75(3.40) | 13.49(11.48) |
| Confusional state | 117 | 3.22(2.68,3.86) | 3.20(177.17) | 1.68(1.38) | 3.20(2.66) | Drug hypersensitivity | 126 | 5.56(4.67,6.63) | 5.51(465.05) | 2.46(2.15) | 5.50(4.61) |
| Bradycardia | 96 | 9.44(7.72,11.54) | 9.39(716.57) | 3.22(2.81) | 9.35(7.65) | Blood creatinine increased | 102 | 5.66(4.66,6.88) | 5.62(386.87) | 2.49(2.14) | 5.61(4.61) |
| Hypokalaemia | 91 | 8.49(6.91,10.44) | 8.44(595.27) | 3.07(2.66) | 8.41(6.84) | Bradycardia | 102 | 7.71(6.34,9.37) | 7.65(587.33) | 2.93(2.55) | 7.62(6.26) |
| Renal impairment | 89 | 6.26(5.08,7.72) | 6.23(390.10) | 2.64(2.25) | 6.22(5.05) | Renal failure | 97 | 2.74(2.24,3.35) | 2.73(106.24) | 1.45(1.13) | 2.72(2.23) |
| Blood creatinine increased | 77 | 6.86(5.48,8.58) | 6.83(381.95) | 2.77(2.34) | 6.81(5.44) | Blood potassium increased | 82 | 19.78(15.90,24.61) | 19.65(1434.49) | 4.28(3.67) | 19.43(15.61) |
| Renal failure | 74 | 3.08(2.45,3.87) | 3.07(103.41) | 1.62(1.24) | 3.07(2.44) | Breast pain | 82 | 113.89(90.98,142.56) | 113.08(8521.77) | 6.73(5.22) | 105.84(84.56) |
| Product substitution issue | 70 | 5.23(4.14,6.62) | 5.21(238.07) | 2.38(1.95) | 5.20(4.11) | Syncope | 80 | 3.85(3.09,4.79) | 3.83(166.99) | 1.93(1.56) | 3.82(3.07) |
| Blood potassium increased | 69 | 22.78(17.96,28.90) | 22.68(1415.40) | 4.49(3.76) | 22.45(17.70) | Renal impairment | 80 | 3.89(3.12,4.85) | 3.87(170.31) | 1.95(1.58) | 3.87(3.10) |
| General physical health deterioration | 65 | 2.81(2.20,3.58) | 2.80(75.28) | 1.48(1.09) | 2.80(2.19) | Cardiac failure | 77 | 3.88(3.10,4.85) | 3.86(162.84) | 1.94(1.56) | 3.85(3.08) |
| Orthostatic hypotension | 58 | 18.87(14.57,24.45) | 18.80(969.22) | 4.22(3.46) | 18.65(14.39) | General physical health deterioration | 77 | 3.25(2.60,4.07) | 3.23(118.78) | 1.69(1.32) | 3.23(2.58) |
| Cardiac failure | 57 | 3.81(2.94,4.95) | 3.80(117.72) | 1.93(1.48) | 3.80(2.93) | Oedema peripheral | 70 | 2.93(2.31,3.70) | 2.92(88.16) | 1.54(1.16) | 2.91(2.30) |
| Product odour abnormal | 54 | 23.69(18.11,30.99) | 23.61(1156.54) | 4.55(3.66) | 23.36(17.86) | Hypokalaemia | 69 | 8.31(6.55,10.53) | 8.26(438.51) | 3.04(2.55) | 8.23(6.49) |
| Blood pressure decreased | 41 | 2.76(2.03,3.76) | 2.76(45.94) | 1.46(0.96) | 2.76(2.03) | Hepatic encephalopathy | 55 | 20.72(15.87,27.05) | 20.63(1014.71) | 4.35(3.53) | 20.38(15.61) |
| Inappropriate antidiuretic hormone secretion | 37 | 17.65(12.77,24.40) | 17.61(574.96) | 4.13(3.14) | 17.47(12.64) | Metabolic acidosis | 53 | 7.40(5.64,9.70) | 7.37(290.60) | 2.88(2.32) | 7.34(5.60) |
| Drug ineffective for unapproved indication | 36 | 3.12(2.25,4.32) | 3.11(51.50) | 1.64(1.08) | 3.11(2.24) | Breast tenderness | 51 | 96.69(72.85,128.31) | 96.26(4541.17) | 6.51(4.65) | 90.97(68.55) |
| Hepatic encephalopathy | 35 | 23.26(16.66,32.46) | 23.20(735.71) | 4.52(3.35) | 22.96(16.45) | Orthostatic hypotension | 48 | 10.63(8.00,14.12) | 10.59(414.19) | 3.40(2.72) | 10.53(7.92) |
| Arrhythmia | 34 | 3.41(2.43,4.77) | 3.40(57.58) | 1.76(1.18) | 3.40(2.43) | Pemphigoid | 41 | 22.64(16.63,30.83) | 22.56(833.56) | 4.48(3.44) | 22.27(16.35) |
| Metabolic acidosis | 33 | 5.22(3.71,7.35) | 5.21(112.00) | 2.38(1.71) | 5.20(3.69) | Nipple pain | 39 | 68.42(49.64,94.30) | 68.19(2478.92) | 6.03(4.18) | 65.50(47.53) |
| Blood potassium decreased | 31 | 3.57(2.51,5.07) | 3.56(57.04) | 1.83(1.21) | 3.56(2.50) | Erectile dysfunction | 39 | 3.01(2.20,4.12) | 3.00(52.02) | 1.58(1.06) | 3.00(2.19) |
| Electrolyte imbalance | 31 | 12.39(8.70,17.65) | 12.37(322.16) | 3.62(2.67) | 12.30(8.64) | Ascites | 32 | 4.54(3.21,6.42) | 4.53(87.76) | 2.18(1.53) | 4.52(3.19) |
| Contraindicated product administered | 27 | 3.69(2.53,5.38) | 3.68(52.66) | 1.88(1.20) | 3.68(2.52) | Breast mass | 32 | 76.63(53.74,109.29) | 76.42(2275.85) | 6.19(4.01) | 73.06(51.23) |
| Product prescribing error | 27 | 3.02(2.07,4.40) | 3.02(36.34) | 1.59(0.94) | 3.01(2.06) | Lactic acidosis | 32 | 4.60(3.25,6.51) | 4.59(89.59) | 2.19(1.54) | 4.58(3.23) |
| Lactic acidosis | 26 | 4.39(2.99,6.45) | 4.38(67.81) | 2.13(1.40) | 4.38(2.98) | Blood urea increased | 31 | 6.36(4.47,9.06) | 6.35(139.20) | 2.66(1.93) | 6.33(4.44) |
| **Eplerenone** | | | | | | | | | | | |
| Acute kidney injury | 63 | 19.23(14.94,24.74) | 18.47(1042.44) | 4.21(3.49) | 18.45(14.34) | Acute kidney injury | 179 | 11.11(9.56,12.91) | 10.64(1567.07) | 3.41(3.11) | 10.62(9.14) |
| Hyperkalaemia | 43 | 77.20(56.98,104.60) | 75.04(3131.13) | 6.22(4.36) | 74.77(55.19) | Hyperkalaemia | 104 | 32.09(26.40,39.02) | 31.26(3029.10) | 4.96(4.31) | 31.06(25.55) |
| Hyponatraemia | 31 | 23.81(16.68,33.99) | 23.35(662.98) | 4.54(3.26) | 23.32(16.34) | Dizziness | 76 | 2.73(2.18,3.43) | 2.70(81.69) | 1.43(1.07) | 2.70(2.15) |
| Hypotension | 24 | 5.55(3.71,8.31) | 5.48(88.08) | 2.45(1.63) | 5.48(3.66) | Hypotension | 67 | 4.40(3.45,5.60) | 4.34(172.56) | 2.12(1.69) | 4.33(3.40) |
| Drug interaction | 22 | 6.83(4.48,10.40) | 6.74(107.79) | 2.75(1.82) | 6.74(4.42) | Cardiac failure | 62 | 9.37(7.29,12.05) | 9.24(455.24) | 3.20(2.66) | 9.22(7.17) |
| Cardiac failure | 21 | 13.58(8.83,20.90) | 13.41(241.21) | 3.74(2.48) | 13.40(8.71) | Drug interaction | 55 | 4.51(3.46,5.89) | 4.46(147.93) | 2.16(1.68) | 4.46(3.41) |
| Dehydration | 14 | 4.30(2.54,7.28) | 4.27(35.14) | 2.09(1.06) | 4.27(2.52) | Renal failure | 39 | 3.28(2.39,4.50) | 3.26(61.21) | 1.70(1.17) | 3.26(2.38) |
| Renal failure | 13 | 5.20(3.01,8.97) | 5.16(43.69) | 2.37(1.22) | 5.16(2.99) | Renal impairment | 36 | 5.22(3.76,7.24) | 5.18(121.42) | 2.37(1.74) | 5.17(3.72) |
| Atrial fibrillation | 11 | 5.68(3.14,10.27) | 5.64(42.05) | 2.50(1.19) | 5.64(3.12) | Blood creatinine increased | 35 | 5.77(4.14,8.05) | 5.73(136.60) | 2.52(1.85) | 5.72(4.10) |
| Bradycardia | 10 | 9.38(5.04,17.48) | 9.33(74.36) | 3.22(1.53) | 9.32(5.01) | Dehydration | 34 | 3.62(2.58,5.07) | 3.59(63.79) | 1.85(1.25) | 3.59(2.56) |
| Blood potassium increased | 8 | 25.08(12.51,50.26) | 24.95(183.76) | 4.64(1.80) | 24.92(12.44) | Oedema peripheral | 34 | 4.24(3.02,5.94) | 4.21(83.33) | 2.07(1.46) | 4.21(3.00) |
| Polyuria | 8 | 47.52(23.70,95.27) | 47.27(361.55) | 5.56(1.98) | 47.17(23.53) | Hyponatraemia | 33 | 8.85(6.28,12.47) | 8.78(227.39) | 3.13(2.34) | 8.77(6.22) |
| Pulmonary oedema | 7 | 7.01(3.33,14.73) | 6.98(35.87) | 2.80(0.97) | 6.98(3.32) | Blood potassium increased | 30 | 21.38(14.92,30.65) | 21.22(575.82) | 4.40(3.16) | 21.14(14.75) |
| Alanine aminotransferase increased | 6 | 4.50(2.02,10.04) | 4.49(16.29) | 2.17(0.49) | 4.49(2.01) | General physical health deterioration | 30 | 3.76(2.63,5.39) | 3.74(60.39) | 1.90(1.26) | 3.74(2.61) |
| Aspartate aminotransferase increased | 6 | 5.10(2.29,11.38) | 5.09(19.72) | 2.35(0.59) | 5.09(2.28) | Syncope | 26 | 3.71(2.52,5.46) | 3.69(51.16) | 1.88(1.19) | 3.69(2.51) |
| Hepatic function abnormal | 6 | 8.98(4.03,20.02) | 8.95(42.35) | 3.16(0.97) | 8.94(4.01) | Glomerular filtration rate decreased | 25 | 29.44(19.85,43.68) | 29.26(678.37) | 4.86(3.24) | 29.09(19.61) |
| Hypokalaemia | 6 | 5.33(2.39,11.88) | 5.31(21.02) | 2.41(0.62) | 5.31(2.38) | Bradycardia | 24 | 5.36(3.59,8.01) | 5.33(84.54) | 2.41(1.60) | 5.33(3.57) |
| Contraindicated product administered | 6 | 7.85(3.52,17.51) | 7.82(35.72) | 2.97(0.89) | 7.82(3.51) | Gynaecomastia | 24 | 3.99(2.67,5.96) | 3.97(53.32) | 1.99(1.25) | 3.97(2.65) |
| Breast pain | 5 | 12.62(5.24,30.37) | 12.58(53.27) | 3.65(0.92) | 12.57(5.22) | Arrhythmia | 19 | 5.22(3.32,8.19) | 5.20(64.37) | 2.38(1.45) | 5.19(3.31) |
| Fracture | 5 | 10.34(4.30,24.89) | 10.31(42.04) | 3.37(0.83) | 10.31(4.28) | Blood pressure decreased | 18 | 3.68(2.32,5.86) | 3.67(35.02) | 1.88(1.02) | 3.67(2.31) |
| Hypothyroidism | 5 | 5.95(2.47,14.32) | 5.94(20.53) | 2.57(0.52) | 5.94(2.47) | Vertigo | 17 | 5.86(3.64,9.44) | 5.84(68.10) | 2.54(1.52) | 5.83(3.62) |
| Abnormal loss of weight | 4 | 25.70(9.63,68.61) | 25.64(94.59) | 4.68(0.82) | 25.60(9.59) | Cardiac failure chronic | 16 | 39.07(23.87,63.97) | 38.92(586.46) | 5.27(2.88) | 38.62(23.59) |
| Altered state of consciousness | 4 | 9.34(3.50,24.92) | 9.32(29.69) | 3.22(0.51) | 9.31(3.49) | Hypokalaemia | 15 | 5.34(3.21,8.87) | 5.32(52.64) | 2.41(1.34) | 5.32(3.20) |
| Angina pectoris | 4 | 6.60(2.47,17.61) | 6.58(18.94) | 2.72(0.34) | 6.58(2.47) | Orthostatic hypotension | 15 | 9.83(5.92,16.34) | 9.80(118.33) | 3.29(1.93) | 9.78(5.89) |
| Blood uric acid increased | 4 | 42.06(15.75,112.31) | 41.95(159.57) | 5.39(0.90) | 41.86(15.68) | Ventricular tachycardia | 14 | 9.15(5.41,15.48) | 9.12(101.13) | 3.19(1.82) | 9.11(5.39) |
| Peripheral coldness | 4 | 11.28(4.23,30.11) | 11.26(37.37) | 3.49(0.59) | 11.25(4.22) | Circulatory collapse | 13 | 9.26(5.37,15.97) | 9.23(95.24) | 3.20(1.76) | 9.21(5.34) |
| Tachypnoea | 4 | 15.06(5.64,40.19) | 15.02(52.33) | 3.91(0.69) | 15.01(5.63) | Oedema | 13 | 3.83(2.22,6.60) | 3.82(27.04) | 1.93(0.89) | 3.82(2.21) |
| Ventricular septal defect | 4 | 40.85(15.30,109.08) | 40.74(154.77) | 5.35(0.89) | 40.66(15.23) | Ejection fraction decreased | 13 | 10.27(5.96,17.72) | 10.24(108.20) | 3.35(1.85) | 10.22(5.93) |
| Left ventricular dysfunction | 4 | 30.85(11.56,82.37) | 30.77(115.06) | 4.94(0.85) | 30.73(11.51) | Product prescribing error | 12 | 4.45(2.52,7.84) | 4.44(31.93) | 2.15(1.01) | 4.43(2.51) |
| Blood albumin decreased | 3 | 17.46(5.62,54.22) | 17.43(46.42) | 4.12(0.33) | 17.41(5.61) | Electrolyte imbalance | 11 | 14.19(7.84,25.66) | 14.15(134.07) | 3.82(1.92) | 14.11(7.80) |
| **Finerenone** | | | | | | | | | | | |
| Glomerular filtration rate decreased | 53 | 417.74(316.06,552.13) | 393.47(20515.8) | 8.60(5.16) | 389.02(294.33) | Blood creatinine increased | 77 | 43.32(34.38,54.58) | 40.60(2970.75) | 5.34(4.41) | 40.49(32.13) |
| Hyperkalaemia | 51 | 157.54(118.70,209.09) | 148.77(7456.07) | 7.21(4.86) | 148.13(111.61) | Glomerular filtration rate decreased | 76 | 309.31(244.70,390.99) | 289.74(21476.4) | 8.15(5.58) | 284.50(225.07) |
| Death | 33 | 3.85(2.72,5.45) | 3.75(67.18) | 1.91(1.29) | 3.75(2.65) | Hyperkalaemia | 72 | 73.87(58.18,93.79) | 69.49(4842.91) | 6.11(4.81) | 69.19(54.49) |
| Blood potassium increased | 27 | 145.37(99.04,213.35) | 141.08(3740.81) | 7.13(4.00) | 140.51(95.73) | Death | 59 | 2.78(2.14,3.61) | 2.69(63.77) | 1.43(1.00) | 2.69(2.07) |
| Blood creatinine increased | 22 | 31.88(20.88,48.67) | 31.13(641.46) | 4.96(3.14) | 31.10(20.37) | Blood potassium increased | 48 | 114.08(85.39,152.42) | 109.55(5128.92) | 6.77(4.67) | 108.80(81.43) |
| Hypotension | 20 | 7.77(4.99,12.11) | 7.62(115.42) | 2.93(1.90) | 7.62(4.89) | Renal impairment | 41 | 19.61(14.36,26.78) | 18.97(698.53) | 4.24(3.28) | 18.95(13.88) |
| Renal impairment | 19 | 21.66(13.75,34.13) | 21.23(366.45) | 4.41(2.75) | 21.22(13.47) | Dizziness | 33 | 3.85(2.72,5.43) | 3.77(67.54) | 1.91(1.30) | 3.77(2.66) |
| Acute kidney injury | 15 | 7.45(4.47,12.41) | 7.34(82.32) | 2.88(1.67) | 7.34(4.41) | Acute kidney injury | 19 | 3.67(2.33,5.77) | 3.63(36.31) | 1.86(1.03) | 3.63(2.30) |
| Hyponatraemia | 14 | 17.86(10.53,30.29) | 17.60(219.31) | 4.14(2.31) | 17.59(10.38) | Hypotension | 16 | 3.36(2.05,5.51) | 3.33(26.19) | 1.74(0.84) | 3.33(2.03) |
| Urine albumin/creatinine ratio increased | 12 | 2937.19(1623.54,5313.76) | 2898.48(32049.5) | 11.38(2.86) | 2672.70(1477.34) | Urine albumin/creatinine ratio increased | 14 | 1821.08(1044.39,3175.39) | 1799.79(22578.3) | 10.66(3.11) | 1614.62(925.98) |
| Chronic kidney disease | 8 | 9.82(4.90,19.70) | 9.74(62.79) | 3.28(1.34) | 9.74(4.86) | Abdominal discomfort | 11 | 3.93(2.17,7.12) | 3.90(23.81) | 1.96(0.81) | 3.90(2.16) |
| Blood pressure decreased | 6 | 6.49(2.91,14.48) | 6.45(27.66) | 2.69(0.76) | 6.45(2.89) | Hyponatraemia | 9 | 7.75(4.02,14.93) | 7.70(52.47) | 2.94(1.29) | 7.69(3.99) |
| Cardiac failure | 6 | 6.43(2.88,14.35) | 6.40(27.33) | 2.68(0.76) | 6.39(2.86) | Blood creatine increased | 6 | 48.13(21.55,107.47) | 47.89(274.65) | 5.58(1.54) | 47.75(21.38) |
| Oedema | 5 | 6.26(2.60,15.07) | 6.23(21.96) | 2.64(0.55) | 6.23(2.59) | Product prescribing issue | 6 | 20.60(9.23,45.98) | 20.51(111.21) | 4.36(1.34) | 20.48(9.18) |
| Hypoglycaemia | 4 | 6.37(2.38,17.00) | 6.34(18.01) | 2.67(0.32) | 6.34(2.38) | Blood potassium decreased | 5 | 10.89(4.52,26.22) | 10.85(44.69) | 3.44(0.86) | 10.84(4.50) |
| Swollen tongue | 4 | 7.36(2.76,19.66) | 7.33(21.89) | 2.87(0.40) | 7.33(2.75) | Glomerular filtration rate increased | 5 | 284.29(117.18,689.67) | 283.10(1380.63) | 8.12(1.37) | 278.10(114.63) |
| Blood potassium abnormal | 3 | 77.68(24.97,241.60) | 77.42(225.81) | 6.27(0.50) | 77.25(24.84) | Blood pressure systolic increased | 4 | 10.79(4.04,28.80) | 10.76(35.38) | 3.43(0.57) | 10.75(4.03) |
| Chromaturia | 3 | 9.59(3.09,29.81) | 9.57(23.01) | 3.26(0.16) | 9.56(3.08) | Blood urea increased | 4 | 7.84(2.94,20.92) | 7.82(23.77) | 2.97(0.43) | 7.81(2.93) |
| Metabolic acidosis | 3 | 7.58(2.44,23.55) | 7.56(17.08) | 2.92(0.07) | 7.56(2.43) | Urine albumin/creatinine ratio decreased | 4 | 5722.36(1819.54,17996.6) | 5703.24(16723.7) | 12.03(0.84) | 4182.64(1329.95) |
| Thirst | 3 | 10.42(3.35,32.36) | 10.39(25.44) | 3.38(0.19) | 10.38(3.34) | Labelled drug-drug interaction medication error | 4 | 17.18(6.43,45.86) | 17.12(60.67) | 4.10(0.72) | 17.10(6.41) |
| Urine albumin/creatinine ratio decreased | 3 | 10323.6(2836.48,37573.7) | 10289.6(23740.6) | 12.95(0.36) | 7915.30(2174.78) | Albumin urine present | 3 | 318.71(101.48,1000.96) | 317.92(928.94) | 8.28(0.52) | 311.62(99.22) |
| Product prescribing issue | 3 | 15.33(4.93,47.62) | 15.28(40.02) | 3.93(0.29) | 15.27(4.91) | Blood pressure diastolic increased | 3 | 37.20(11.96,115.66) | 37.11(105.16) | 5.21(0.44) | 37.02(11.91) |
| Glomerular filtration rate decreased | 53 | 417.74(316.06,552.13) | 393.47(20515.8) | 8.60(5.16) | 389.02(294.33) | Blood sodium decreased | 3 | 7.72(2.49,23.98) | 7.70(17.50) | 2.95(0.08) | 7.70(2.48) |
|  |  |  |  |  |  | Proteinuria | 3 | 6.59(2.12,20.47) | 6.58(14.19) | 2.72(0.01) | 6.57(2.12) |
|  |  |  |  |  |  | Renal pain | 3 | 13.38(4.31,41.57) | 13.35(34.26) | 3.74(0.26) | 13.34(4.29) |

TableS13. Age-Stratified Subgroup Analysis of AEs Associated With MRAs in FAERS

| **<18y** | | **18-44y** | | **45-64y** | | **≥65y** | |
| --- | --- | --- | --- | --- | --- | --- | --- |
| **PT** | **N** | **PT** | **N** | **PT** | **N** | **PT** | **N** |
| **Spironolactone** | | | | | | | |
| Hyperkalaemia | 18 | Off label use | 87 | Hyperkalaemia | 219 | Hyperkalaemia | 951 |
| Drug interaction | 13 | Hyperkalaemia | 23 | Acute kidney injury | 193 | Acute kidney injury | 818 |
| Contraindicated product administered | 10 | Acute kidney injury | 21 | Drug interaction | 108 | Drug interaction | 481 |
| Hyponatraemia | 9 | Product substitution issue | 17 | Gynaecomastia | 96 | Hyponatraemia | 362 |
| Hypokalaemia | 7 | Gynaecomastia | 15 | Hypotension | 81 | Dehydration | 295 |
| Low birth weight baby | 4 | Hyponatraemia | 13 | Drug hypersensitivity | 76 | Hypotension | 288 |
| Drug reaction with eosinophilia and systemic symptoms | 4 | Hypokalaemia | 11 | Hyponatraemia | 69 | Bradycardia | 160 |
| Cardiomegaly | 3 | Drug ineffective for unapproved indication | 9 | Condition aggravated | 67 | Drug hypersensitivity | 132 |
| Pathological fracture | 3 | Product odour abnormal | 9 | Dehydration | 63 | Blood creatinine increased | 124 |
| Osteopenia | 3 | Liver injury | 8 | Hypokalaemia | 45 | Renal failure | 123 |
| Neonatal hyponatraemia | 3 | Hair growth abnormal | 7 | Breast pain | 41 | Renal impairment | 116 |
| Dysmorphism | 3 | Pollakiuria | 7 | Blood creatinine increased | 39 | General physical health deterioration | 113 |
| Hypertransaminasaemia | 3 | Ventricular tachycardia | 7 | Hepatic encephalopathy | 38 | Gynaecomastia | 107 |
| Maternal exposure during pregnancy | 3 | Ascites | 6 | Renal impairment | 34 | Hypokalaemia | 96 |
| Hypocalvaria | 3 | Breast tenderness | 6 | Blood potassium increased | 33 | Blood potassium increased | 87 |
|  |  | Torsade de pointes | 6 | Syncope | 32 | Orthostatic hypotension | 76 |
|  |  | Toxic epidermal necrolysis | 6 | Cardiac failure | 27 | Pemphigoid | 66 |
|  |  | Breast enlargement | 5 | Bradycardia | 25 | Metabolic acidosis | 59 |
|  |  | Breast swelling | 5 | Metabolic acidosis | 25 | Chronic kidney disease | 57 |
|  |  | Hepatic encephalopathy | 5 | Orthostatic hypotension | 25 | Hepatic encephalopathy | 51 |
|  |  | Intracranial pressure increased | 5 | Lactic acidosis | 21 | Inappropriate antidiuretic hormone secretion | 46 |
|  |  | Oral pain | 5 | Blood potassium decreased | 19 | Breast pain | 44 |
|  |  | Tubulointerstitial nephritis | 5 | Product substitution issue | 18 | Electrolyte imbalance | 41 |
|  |  | Haemodynamic instability | 5 | Breast mass | 16 | Blood urea increased | 40 |
|  |  | Invasive ductal breast carcinoma | 5 | Erectile dysfunction | 16 | Atrioventricular block complete | 35 |
|  |  | Blepharospasm | 4 | Breast tenderness | 15 | Atrioventricular block | 32 |
|  |  | Blood potassium increased | 4 | Electrolyte imbalance | 15 | Glomerular filtration rate decreased | 31 |
|  |  | Coeliac disease | 4 | Nipple pain | 15 | Rash maculo-papular | 31 |
|  |  | Extrasystoles | 4 | Arrhythmia | 14 | Breast tenderness | 27 |
|  |  | Flank pain | 4 | Breast enlargement | 13 | Blood sodium decreased | 25 |
| **Eplerenone** | | | | | | | |
|  |  | Cardiac failure | 7 | Acute kidney injury | 41 | Acute kidney injury | 189 |
|  |  | Acute kidney injury | 7 | Hyperkalaemia | 24 | Hyperkalaemia | 115 |
|  |  | Renal failure | 5 | Cardiac failure | 20 | Hypotension | 66 |
|  |  | Anaemia | 4 | Drug interaction | 20 | Cardiac failure | 54 |
|  |  | Hyponatraemia | 4 | Hypotension | 20 | Hyponatraemia | 54 |
|  |  | Hypotension | 4 | Syncope | 17 | Drug interaction | 51 |
|  |  | Ejection fraction decreased | 4 | Blood creatinine increased | 11 | Dehydration | 36 |
|  |  | Drug ineffective for unapproved indication | 4 | General physical health deterioration | 11 | Renal failure | 33 |
|  |  | Altered state of consciousness | 3 | Vertigo | 10 | Oedema peripheral | 31 |
|  |  | Blood pressure decreased | 3 | Blood potassium increased | 9 | Renal impairment | 31 |
|  |  | Circulatory collapse | 3 | Cardiac disorder | 9 | Bradycardia | 29 |
|  |  | Coma | 3 | Cardiac failure congestive | 8 | Blood potassium increased | 26 |
|  |  | Gestational diabetes | 3 | Hypokalaemia | 8 | Blood creatinine increased | 24 |
|  |  | Renal impairment | 3 | Atrial fibrillation | 7 | Glomerular filtration rate decreased | 22 |
|  |  |  |  | Blood creatine phosphokinase increased | 7 | Orthostatic hypotension | 17 |
|  |  |  |  | Renal impairment | 7 | Arrhythmia | 14 |
|  |  |  |  | Arrhythmia | 6 | Cardiac failure chronic | 13 |
|  |  |  |  | Electrolyte imbalance | 6 | Ventricular tachycardia | 12 |
|  |  |  |  | Gynaecomastia | 6 | Circulatory collapse | 10 |
|  |  |  |  | Type 2 diabetes mellitus | 6 | Gynaecomastia | 10 |
|  |  |  |  | Product prescribing error | 6 | Lower respiratory tract infection | 9 |
|  |  |  |  | Cardiac failure chronic | 5 | Polyuria | 9 |
|  |  |  |  | Cold sweat | 5 | Eosinophilia | 8 |
|  |  |  |  | Dyspnoea exertional | 5 | Pemphigoid | 8 |
|  |  |  |  | Glomerular filtration rate decreased | 5 | Labelled drug-drug interaction medication error | 8 |
|  |  |  |  | Hyponatraemia | 5 | Fracture | 7 |
|  |  |  |  | Ascites | 4 | Rales | 7 |
|  |  |  |  | Gout | 4 | Left ventricular dysfunction | 7 |
|  |  |  |  | Hyperthyroidism | 4 | Contraindicated product administered | 7 |
|  |  |  |  | Presyncope | 4 | Atrial flutter | 6 |
| **Finerenone** | | | | | | | |
|  |  | Off label use | 4 | Glomerular filtration rate decreased | 19 | Death | 71 |
|  |  | Glomerular filtration rate decreased | 3 | Blood creatinine increased | 17 | Hyperkalaemia | 58 |
|  |  |  |  | Blood potassium increased | 15 | Blood creatinine increased | 52 |
|  |  |  |  | Renal impairment | 14 | Glomerular filtration rate decreased | 48 |
|  |  |  |  | Hyperkalaemia | 13 | Renal impairment | 33 |
|  |  |  |  | Chronic kidney disease | 4 | Dizziness | 31 |
|  |  |  |  | Oedema | 3 | Blood potassium increased | 28 |
|  |  |  |  | Urine albumin/creatinine ratio increased | 3 | Hypotension | 20 |
|  |  |  |  |  |  | Urine albumin/creatinine ratio increased | 9 |
|  |  |  |  |  |  | Blood creatine increased | 5 |
|  |  |  |  |  |  | Blood potassium decreased | 5 |
|  |  |  |  |  |  | Blood pressure systolic increased | 4 |
|  |  |  |  |  |  | Albuminuria | 3 |
|  |  |  |  |  |  | Albumin urine present | 3 |
|  |  |  |  |  |  | Glomerular filtration rate increased | 3 |
|  |  |  |  |  |  | Product prescribing issue | 3 |
